# ChIP-mini: a low-input ChIP-exo protocol for elucidating DNA-binding protein dynamics in intracellular pathogens

**DOI:** 10.1093/nar/gkaf009

**Published:** 2025-01-27

**Authors:** Joon Young Park, Minchang Jang, Eunna Choi, Sang-Mok Lee, Ina Bang, Jihoon Woo, Seonggyu Kim, Eun-Jin Lee, Donghyuk Kim

**Affiliations:** School of Energy and Chemical Engineering, Ulsan National Institute of Science and Technology (UNIST), Ulsan, 44919, Republic of Korea; School of Energy and Chemical Engineering, Ulsan National Institute of Science and Technology (UNIST), Ulsan, 44919, Republic of Korea; Department of Life Sciences, College of Life Sciences and Biotechnology, Korea University, Seoul, 02841, Republic of Korea; School of Energy and Chemical Engineering, Ulsan National Institute of Science and Technology (UNIST), Ulsan, 44919, Republic of Korea; School of Energy and Chemical Engineering, Ulsan National Institute of Science and Technology (UNIST), Ulsan, 44919, Republic of Korea; School of Energy and Chemical Engineering, Ulsan National Institute of Science and Technology (UNIST), Ulsan, 44919, Republic of Korea; Department of Life Sciences, College of Life Sciences and Biotechnology, Korea University, Seoul, 02841, Republic of Korea; Department of Life Sciences, College of Life Sciences and Biotechnology, Korea University, Seoul, 02841, Republic of Korea; School of Energy and Chemical Engineering, Ulsan National Institute of Science and Technology (UNIST), Ulsan, 44919, Republic of Korea

## Abstract

Genome-wide identification of binding profiles for DNA-binding proteins from the limited number of intracellular pathogens in infection studies is crucial for understanding virulence and cellular processes but remains challenging, as the current ChIP-exo is designed for high-input bacterial cells (>10^10^). Here, we developed an optimized ChIP-mini method, a low-input ChIP-exo utilizing a 5,000-fold reduced number of initial bacterial cells and an analysis pipeline, to identify genome-wide binding dynamics of DNA-binding proteins in host-infected pathogens. Applying ChIP-mini to intracellular *Salmonella* Typhimurium, we identified 642 and 1,837 binding sites of H-NS and RpoD, respectively, elucidating changes in their binding position and binding intensity during infection. Post-infection, we observed 21 significant reductions in H-NS binding at intergenic regions, exposing the promoter region of virulence genes, such as those in *Salmonella* pathogenicity islands-2, 3 and effectors. Furthermore, we revealed the crucial phenomenon that novel and significantly increased RpoD bindings were found within regions exhibiting diminished H-NS binding, thereby facilitating substantial upregulation of virulence genes. These findings markedly enhance our understanding of how H-NS and RpoD simultaneously coordinate the transcription initiation of virulence genes within macrophages. Collectively, this work demonstrates a broadly adaptable tool that will enable the elucidation of DNA-binding protein dynamics in diverse intracellular pathogens during infection.

## Graphical Abstract

**Graphical Abstract:**
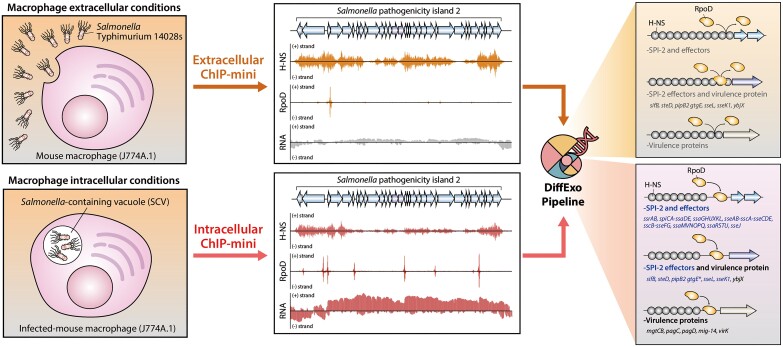

## Introduction

Over the past two decades, Chromatin immunoprecipitation (ChIP) methodology has been combined with whole-genome DNA microarray (ChIP-chip) or deep sequencing (ChIP-seq) to elucidate the genome-wide binding profiles of DNA-binding proteins (1,2). However, these methods suffer from low resolution due to the size heterogeneity of randomly sheared immunoprecipitated-DNA (IP-DNA) required for alignment to the genome, hindering the identification of complete and high-resolution binding sites (3). To address this limitation, chromatin immunoprecipitation with exonuclease treatment (ChIP-exo) was developed. This technique uses lambda exonuclease to digest the 5’ end of IP-DNA up to the protein-DNA crosslinking point, achieving near-base pair resolution (3,4).

ChIP-exo has been further modified for bacterial use, enabling the identification of *in vivo* binding profiles of transcription factors (TFs) on the bacterial genome (5–9). To minimize polymerase chain reaction (PCR) bias in the sequencing library of bacteria, amplification cycles were set to 15–24 cycles, as used in the eukaryotic ChIP-exo method (3,4). As a result, the ChIP-exo method for bacteria has come to require a large number of bacterial cells (in the range of 10^10^–10^11^) due to the complexity of the experimental process and the low concentration of IP-DNA. To address this limitation, ChIP-exo 5.0 was recently developed to effectively reduce enzymatic steps and increase library yield, resulting in the generation of high-quality libraries using low-input material (10). This advanced method allowed around 10^9^ bacterial cells for identification of RpoD binding profiles in *Escherichia coli* (11), demonstrating the lowest input used in ChIP-exo studies for bacteria. Despite these efforts, ChIP-exo using fewer than 10^8^ bacterial cells has not yet been tested, which is essential for scientific investigations where bacterial cell availability is limited.

Specifically, procedures designed for high-input bacterial cells render it impractical for infection studies involving extremely low numbers of intracellular bacteria. For example, a low concentration of IP-DNA before library amplification can result in low library complexity and insufficient resolution for the identification of genome-wide binding profiles (12). Moreover, excessive use of antibodies relative to the number of bacterial cells can cause non-specific binding to abundant host DNA, resulting in serious host DNA contamination in the sequencing library. Therefore, an optimized method was necessary that could utilize a low number of initial bacterial cells for studying the transcriptional regulatory networks (TRNs) of intracellular pathogen within host.

*Salmonella enterica* serovar Typhimurium is a non-typhoidal Gram-negative pathogen responsible for worldwide foodborne illnesses, primarily causing gastroenteritis, and is capable of infecting a wide range of host cell types (13). Notably, macrophages, which serve as the primary defense against invading bacteria, are also crucial colonization niches of *S*. Typhimurium (14). The ability of a pathogen to survive and replicate within macrophages facilitates its establishment of systemic disease in susceptible hosts (14–16). Understanding this ability requires elucidating the TRNs of *S*. Typhimurium within macrophages, a complex challenge that involves comprehending host–microbe interactions from a transcriptional regulation standpoint. To reveal these intricate networks involving various virulence mechanisms orchestrated by pathogens during infection, high-resolution binding profiles of DNA-binding proteins for integration with transcriptomic data are essential.

To gain insight into the transcriptional regulatory mechanisms of *S*. Typhimurium virulence genes during infection, it is necessary to elucidate the genome-wide binding dynamics of two DNA-binding proteins, H-NS and RpoD. H-NS, also known as histone-like nucleoid structuring protein, acts as a global transcription silencer in Gram-negative bacteria (17–19). H-NS binds to AT-rich DNA associated with horizontally acquired genes, including *Salmonella* pathogenicity islands (SPIs) and various virulence genes, playing a pivotal role in alleviating fitness costs by suppressing those genes under normal conditions (17,20). Additionally, RpoD, the housekeeping sigma factor, has been extensively studied for recognizing the majority of promoters in *S*. Typhimurium and *E. coli* in previous research (8,9,21–23). The predominant binding sites of RpoD suggest its significant role in governing the transcription initiation of various virulence genes. Despite their importance, detailed genome-wide binding maps and binding dynamics of these DNA-binding proteins are still largely unknown within host cells.

Here, we present the ChIP-mini, a method suitable for working with as few as 4.8×10^6^ bacterial cells, which faithfully maintains relevant binding profiles generated by the traditional ChIP-exo method. This method was successfully applied during the traditional infection process, identifying genome-wide binding maps of H-NS and RpoD with near-base pair resolution in both macrophage extracellular and intracellular bacteria (extra- and intracellular ChIP-mini). For further validation, we constructed the DiffExo pipeline (ChIP-exo peak normalization pipeline) to identify binding profiles with statistically different intensities between macrophages extracellular and intracellular conditions. Utilizing this pipeline, we are able to identify detailed alterations in the binding intensity of both DNA-binding proteins during infection, offering insights into potential relationships for transcription initiation between H-NS and RpoD binding upstream of various virulence genes.

Ultimately, using the ChIP-mini workflow, we reveal for the first time comprehensive genome-wide dynamics of H-NS and RpoD in *S*. Typhimurium during the transition from macrophage extracellular to intracellular environments. Thus, this study represents a significant advancement in elucidating the TRNs of this infectious intracellular pathogen and provides a robust workflow for future studies aiming to decode the comprehensive genome-wide regulatory roles of various DNA-binding proteins during infection.

## Materials and methods

### Bacterial strains and growth conditions

All strains used in this study are *E. coli* K-12 MG1655, *S*. Typhimurium 14028s, and their derivatives. For ChIP experiments, *S*. Typhimurium harboring *hns*-8myc and *E. coli* harboring *rpoH*-8myc were generated through a λ red-mediated site-specific recombination system as described previously (24). For ChIP-exo and ChIP-mini experiments, glycerol stock of *E. coli* and *S*. Typhimurium strains were inoculated into M9 minimal media with 0.2% (w/v) glucose. M9 minimal media was also supplemented with 1 ml trace element solution (100x) containing 1 g EDTA, 29 mg ZnSO_4_·7H_2_O, 198 mg MnCl_2_·4H_2_O, 254 mg CoCl_2_·6H_2_O, 13.4 mg CuCl_2_ and 147 mg CaCl_2_. The inoculated culture was incubated at 37°C overnight with constant agitation. Culture was then diluted into 100 ml of fresh minimal media (1/200 dilution) and cultured at 37°C with agitation in a water bath to the mid-exponential phase (OD_600_ ≈ 0.6–0.7). For extracellular and intracellular ChIP-mini experiments, glycerol stocks of *S*. Typhimurium wild-type and *hns*-8myc strains were inoculated into Luria-Bertani (LB) media. Cultures were incubated at 37°C overnight with agitation, then used to inoculate the 2 ml of fresh LB media. The fresh culture was incubated at 37°C overnight with agitation in a shaking incubator. For fluorescent image analysis measuring *Salmonella* infection efficiency, glycerol stock of *S*. Typhimurium harboring pFPV25.1 vector was inoculated to LB media with 100 μM ampicillin and incubated at 37°C overnight with constant agitation. The culture was then diluted into 2 ml of fresh LB media with 100 μM ampicillin and cultured at 37°C overnight with agitation in a shaking incubator.

### Cell culture

Mouse macrophage-like cells (J774A.1, RAW 264.7) and human epithelial cells (HeLa, INT 407) employed in this study were cultured in Dulbecco's modified Eagle's medium (DMEM) supplemented with 10% (v/v) fetal bovine serum (FBS) and antibiotic antimycotic at 37°C with 5% CO_2_ in a humidified incubator. Cells were seeded in six-well plates at a plating density of 5×10^5^ per well for fluorescent image analysis. For ChIP-mini applications, cells were seeded in 75T flasks at a plating density of 6×10^6^ per flask.

### Fluorescent image analysis of *Salmonella* infection

Four types of host cells (J774A.1, RAW 264.7, HeLa, and INT 407) were seeded in six-well plates at a plating density of 5×10^5^ per well under the culture conditions described above. Overnight-grown *S*. Typhimurium harboring pFPV25.1 vector in LB media with 100 μM ampicillin were added to the macrophages (J774A.1, RAW 264.7) at a multiplicity of infection (MOI) of 10 (25,26). Additionally, overnight-grown bacteria were diluted in fresh LB media (1/20 dilution) and cultured at 37°C with agitation in shaking incubator for 3 h before epithelial cells infection (27). Cultures were added to the epithelial cells (HeLa, INT 407) at an MOI of 100. The plates were centrifuged at 500 xg for 5 min at room temperature and incubated for an additional 30 min. To remove extracellular bacteria, two rounds of phosphate-buffered saline (PBS) washing were conducted, followed by DMEM supplemented with 10% (v/v) FBS and 150 μg ml^−1^ gentamicin. After 1 h, the DMEM was replaced to fresh DMEM containing 15 μg ml^−1^ gentamicin, and the plates were then incubated for 6 h at 37°C with 5% CO_2_ in a humidified incubator. Fluorescent images were obtained using EVOS FL Auto imaging system.

### Quantification of extra- and intracellular *S*. Typhimurium

Intramacrophage survival assays were conducted using the macrophage-like cell line (J774A.1) (26). Macrophages (3 × 10^5^) in DMEM supplemented with 10% FBS were seeded in 24-well plates and cultured at 37°C. Overnight-grown bacteria were added to the macrophages at an MOI of 10. The plates were centrifuged at 500 xg for 5 min at room temperature and then incubated for 30 min. Following infection, the supernatant containing extracellular bacteria was collected and plated on LB plates to quantify the Colony-forming unit (CFU) of extracellular bacteria. Then, remaining extracellular bacteria were washed twice with PBS and killed by incubation in DMEM supplemented with 10% FBS and 150 μg ml^−1^ gentamycin for 1 h. To measure bacterial counts at 1 h, infected macrophages were lysed with PBS containing 0.1% Triton X-100, and the lysates were plated on LB plates with appropriate dilutions. For measuring the number of bacteria at 6 h, the DMEM was replaced after 1 h with fresh DMEM containing 15 μg ml^−1^ gentamycin, and the incubation was continued at 37°C. After 6 h, cells were lysed with PBS containing 0.1% Triton X-100 and plated on LB plates. Infection efficiency was calculated by dividing the number of bacteria recovered at 1 h by the number of macrophages (# of recovered bacteria / # of macrophages × 100%). Fold change of intracellular replication was calculated by dividing the number of bacteria recovered at 6 h by the number of bacteria present at 1 h. All experiments were performed in duplicates, and the results are representative of at least three independent experiments.

### ChIP-exo experiment

ChIP-exo for bacteria was performed following the procedures described in previous studies (5–7). To identify RpoD and RpoH binding profiles in *E. coli*, 2.4 × 10^10^ initial cells of *E. coli* wild-type and *rpoH*-8myc tagged cells (50 ml of cultured media, OD_600_ ≈ 0.6–0.7) in M9 minimal media at mid-exponential phase were used to crosslink DNA and RpoD. Additionally, 2.4 × 10^10^ of initial *S*. Typhimurium *hns*-8myc tagged cells in M9 minimal media at mid-exponential phase were harvested to identify H-NS binding profiles. We added 1.4 ml of formaldehyde (37% w/w) to 50 ml of the initial cultures at final to achieve a final formaldehyde concentration (w/w) of 1% and incubated them for 25 min at room temperature on a rocker. The crosslinked cultures were then centrifuged at 3,500 xg at 4°C for 15 min, followed by three washes with 50 ml of ice-cold TBS. Crosslinked cells were resuspended in 270.5 μl of lysis buffer (10 mM Tris-HCl, pH 7.5, 100 mM NaCl and 1 mM EDTA) including protease inhibitor cocktail (Sigma-Aldrich) and lysozyme (Qiagen) and diluted in 275 μl of IP buffer (100 mM Tris-HCl, pH 7.5, 200 mM NaCl, 1 mM EDTA and 2% Triton X-100 (w/v)) to lyse cells and fragment DNA using sonication (25 min with 50 s on and 10 s off intervals and amplitude 50%). Subsequently, we isolated the DNA bound to RpoD and RpoH from the crosslinked *E. coli* and H-NS from the crosslinked *S*. Typhimurium lysates using ChIP. ChIP was performed with 6 μl of RpoD antibody (2G10, Biolegend) or 15 μl of c-Myc antibody (9E10, Biolegend). Following this, 60 μl of Dynabeads Pan Mouse IgG magnetic beads (Invitrogen) was added, followed by stringent washings (21).

The sheared DNA on Dynabeads was repaired using 100 μl of the NEBNext End Repair Module (New England Biolabs). Around 50 μl of the dA-Tailing Module (New England Biolabs) added a single dA overhang, followed by ligation of the first adaptor (5′-phosphorylated) using 50 μl of the NEBNext Quick Ligation Module (New England Biolabs). Nick repair was performed using 50 μl of PreCR Repair Mix (New England Biolabs). DNA was treated with 50 μl of Lambda exonuclease Mix (New England Biolabs) and RecJf exonuclease Mix (New England Biolabs) to digest the 5’ ends of DNA. Exonuclease-treated chromatin was eluted from the beads using 200 μl of elution buffer (50 mM Tris-HCl, pH 8.0, 1 mM EDTA and 1% SDS (w/v)) by overnight incubation at 65°C, and 200 μl of TE buffer was added to make up the proper volume for Phenol:Chloroform:Isoamyl Alcohol (PCIA) purification and ethanol precipitation. To deplete RNA and reverse the protein–DNA crosslink, RNase A and Proteinase K were used, respectively. RNAs and proteins-removed samples purified by PCIA/ethanol precipitation were used to perform primer extension and second-adapter ligation with the following modifications. The DNA samples were incubated for primer extension of second-strand synthesis as described previously (5), followed by dA tailing using 8 μl of the dA-Tailing Module (New England Biolabs) and second-adaptor ligation using 27 μl of the NEBNext Quick Ligation Module (New England Biolabs). To remove 3’ overhangs, 30 μl of T4 DNA polymerase Mix (New England Biolabs) was used. The DNA samples from each step in Stage 2 were purified by QIAquick or MinElute Spin Columns (Qiagen).

The DNA samples purified by GeneRead Size Selection Kit (Qiagen) were enriched by PCR using KAPA HiFi HotStart ReadyMix (New England Biolabs). The amplified DNA samples were purified again by GeneRead Size Selection Kit (Qiagen) and quantified using Qubit dsDNA HS Assay Kit (Life Technologies). Traditional ChIP-exo experiments were performed in biological duplicates.

### ChIP-seq experiment

For comparison of resolution in traditional ChIP-exo and ChIP-mini with ChIP-seq, 2.4×10^10^ of initial *S*. Typhimurium *hns*-8myc tagged cells at mid-exponential phase in M9 minimal media were crosslinked DNA and H-NS by adding 1.4 ml of formaldehyde (37% w/w) to achieve a final formaldehyde concentration (w/w) of 1%, followed by incubation for 25 min at room temperature on a rocker. The crosslinked cultures were centrifuged at 3,500 xg at 4°C for 15 min, followed by three washes with 50 ml of ice-cold TBS. Crosslinked cells were resuspended in 270.5 μl of lysis buffer including protease inhibitor cocktail (Sigma-Aldrich) and lysozyme (Qiagen) and diluted in 275 μl of IP buffer to lyse cells and fragment DNA using sonication (25 min with 50 s on and 10 s off intervals and amplitude 50%). Next, we isolated the DNA bound to H-NS from the crosslinked *S*. Typhimurium lysate by ChIP with the specific antibody recognizing c-Myc (9E10, Biolegend) and Dynabeads Pan Mouse IgG magnetic beads (Invitrogen). This process was followed by stringent washings (21). The sheared DNA on Dynabeads was eluted from the beads using 200 μl of elution buffer by overnight incubation at 65°C, and 200 μl of TE buffer was added to make up the proper volume for PCIA and ethanol precipitation. RNase A and Proteinase K were used to deplete RNA and reverse the protein–DNA crosslink, respectively. RNA and protein-removed samples purified by PCIA/ethanol precipitation were used to sequencing library construction. Around 50 μl of the dA-Tailing Module (New England Biolabs) added a single dA overhang, followed by ligation of the adaptor using 50 μl of the NEBNext Quick Ligation Module (New England Biolabs). The DNA samples from each step were purified by QIAquick or MinElute Spin Columns (Qiagen). Before sequencing library amplification, the DNA sample was purified by GeneRead Size Selection Kit (Qiagen) and enriched by PCR using KAPA HiFi HotStart ReadyMix (New England Biolabs). The amplified DNA samples were purified again by GeneRead Size Selection Kit (Qiagen) and quantified using Qubit dsDNA HS Assay Kit (Life Technologies). ChIP-seq experiments were performed in biological duplicates.

### ChIP-mini experiment

A detailed, ChIP-mini method depending on number of initial bacterial cells is presented in supplementary methods. The minimalized ChIP-mini method consists of four major parts as follows: (1) DNA fragmentation, (2) exonuclease digestion of antibody-TF complex (Stage 1), (3) reverse crosslinking and (4) construction of sequencing library (Stage 2). Crosslinked cells were resuspended in 108.2 μl of lysis buffer mix, protease inhibitor cocktail and lysozyme. Around 110 μl of the IP buffer was added to lyse cells. DNA for RpoD, RpoH and H-NS samples were fragmented using sonication (40 min with 50 s on and 10 s off intervals and amplitude 50%). ChIP was proceeded using 1.5 μl of RpoD antibody (2G10, Biolegend) or 3.75 μl of c-Myc antibody (9E10, Biolegend). Next, 15 μl of Dynabeads Pan Mouse IgG magnetic beads (Invitrogen) was used, followed by stringent washings.

In Stage 1, the sheared DNA on Dynabeads was repaired using 10 μl of the NEBNext End Repair Module (New England Biolabs), followed by 5 μl of the dA tailing Module (New England Biolabs), first-adaptor ligation using NEBNext Quick Ligation Module (New England Biolabs), Nick repair Mix (New England Biolabs), Lambda exonuclease Mix (New England Biolabs) and RecJf exonuclease Mix (New England Biolabs). Exonuclease-treated chromatin was eluted from the beads using a 20 μl elution buffer by overnight incubation at 65°C. RNase A and Proteinase K were used to remove RNAs and to reverse-crosslink chromatin, respectively. To reduce IP-DNA loss and provide IP-DNA size selectivity (>100 bp), these RNA- and protein-removed samples were purified using 2.5x HiAccuBead.

In Stage 2, to retain more IP-DNA and optimize the experimental materials for constructing the sequencing library, we developed a one-tube procedure for DNA purification to minimize the loss of DNA samples. This procedure allows the use of an enzyme mixture from each step as a bead-elution buffer, introducing the beads only once and adjusting the concentration of polyethylene glycol (PEG) solution (20% PEG 8000/2.5 M NaCl) to purify DNA of the appropriate size generated at each step. By adopting a one-tube procedure, ChIP-mini enables an average reduction of 52% in the volume of reagents required compared to traditional ChIP-exo and minimizes IP-DNA loss to multiple column-based purifications. The DNA samples were incubated for primer extension of second-strand synthesis as described previously (5). DNA with the second-strand synthesized was purified by 2.5x HiAccuBead (AccuGene) and eluted in 10 μl elution buffer consisting of 1.6 μl dA-Tailing Module (New England Biolabs) and 8.4 μl-filtered DW. After dA-tailing, the enzyme was deactivated at 60°C for 30 min, followed by the addition of 15 μl of the NEBNext Quick Ligation Module (New England Biolabs) for the second-adapter ligation. This adapter-ligated DNA with beads was purified by adding 25 μl PEG. Subsequently, the 3’ overhang was removed using 10 μl of the T4 DNA polymerase Mix (New England Biolabs) as the elution buffer. This DNA sample with beads underwent purification by adding 10 μl of PEG, followed by elution with 20 μl of filtered DW.

Finally, the DNA sample was enriched by PCR using KAPA HiFi HotStart ReadyMix (New England Biolabs). The amplified DNA samples were purified again by 1.0x HiAccuBead (AccuGene) and quantified using Qubit dsDNA HS Assay Kit (Life Technologies). ChIP-mini experiments were also performed in biological duplicates.

### Extracellular and intracellular ChIP-mini experiments

Both ChIP-mini applications were conducted using J774A.1 cells with *S*. Typhimurium wild-type or *hns*-8myc tagged strains. In addition, validation of ChIP-mini applications was performed using RAW 264.7, HeLa, and INT 407 with *S*. Typhimurium *hns*-8myc tagged strain. Four types of cells were seeded in 75T flasks at a plating density of 6×10^6^ per flask under the culture conditions described above. Overnight-grown bacteria were added to the macrophages (J774A.1, RAW 264.7) at a MOI of 10. Additionally, overnight-grown bacteria were diluted in fresh LB media (1/20 dilution) and cultured at 37°C with agitation in shaking incubator for 3 h before epithelial cells infection (27). Cultures were added to the epithelial cells (HeLa, INT 407) at a MOI of 100. The 75T flasks were centrifuged at 500 xg for 5  min at room temperature and incubated for an additional 30 min (25,26).

Following infection, 10 ml of DMEM containing extracellular bacteria was transferred to a centrifuge tube and crosslinked with formaldehyde (37% w/w) for 25 min at room temperature to achieve a final formaldehyde concentration (w/w) of 1%. Next, the crosslinked cells were centrifuged at 4,000 xg for 15 min at 4°C and washed three times with ice-cold TBS. The cell pellet containing extracellular bacteria was used for extracellular ChIP-mini. The remaining extracellular bacteria were washed three times with PBS and killed by 1 h of incubation with DMEM supplemented with 10% FBS and 150 μg ml^−1^ gentamycin. For post-infection, the DMEM was replaced to fresh DMEM containing 10% FBS with 15 μg mL^−1^ gentamicin, and the flasks were incubated at 37°C.

For *S*. Typhimurium-infected J774A.1 cells, co-crosslinking was performed at 2, 4, 6, 9 and 21 h post-infection. For *S*. Typhimurium-infected RAW 264.7 cells and epithelial cells (HeLa, INT 407), co-crosslinking was conducted at 6 hours post-infection. In each case, co-crosslinking was performed with formaldehyde (37% w/w) to reach a final concentration of 1% (w/w) formaldehyde for 25 min at room temperature. Co-crosslinked cells were centrifuged at 500 xg for 5 min at 4°C and washed three times with ice-cold TBS. Next, co-crosslinked cells were transferred to 1.75 ml tube and lysed with 1% Triton X-100. Lysed cells were centrifuged at 20,000 xg for 5 min at 4°C. The cell pellet containing intracellular bacteria was utilized for intracellular ChIP-mini. A detailed, ChIP-mini application experiments is presented in supplementary methods.

### Extracellular and intracellular RNA-seq experiments

Extracellular and intracellular *S*. Typhimurium wild-type cells from macrophages were cultured under identical growth conditions as described for the ChIP-mini applications. For total RNA isolation of extracellular bacteria, 10 ml DMEM containing extracellular *S*. Typhimurium after infection were mixed with 30 ml RNAprotect Bacteria Reagent (Qiagen). Samples were immediately mixed by vortexing for 5 s, incubated at room temperature for 5 min, and then centrifuged at 500 xg for 10 min. The supernatant was decanted, and any residual supernatant was removed by inverting the tube once onto a paper towel. For total RNA isolation of intracellular bacteria, *S*. Typhimurium-infected macrophages were mixed with 30 ml of RNAprotect Bacteria Reagent (Qiagen) and incubated at room temperature for 5 min. After decanting the supernatant, the infected macrophages were transferred to 1.75 mL microtube and subsequently centrifuged at 20,000 xg for 5 min. Total RNA samples were then isolated using the RNeasy Plus Mini kit (Qiagen) according to the manufacturer's instructions. The samples were quantified using a NanoDrop 1000 spectrophotometer (Thermo Scientific), and the quality of the isolated RNA was assessed with an RNA 6000 Pico Kit (Agilent) on an Agilent 2100 Bioanalyzer. Ribosomal RNA depletion was subsequently carried out with the Ribo-Zero Plus rRNA Depletion Kit (Illumina). Following rRNA depletion, a paired-end, strand-specific RNA-seq library was constructed using the KAPA Stranded RNA-seq Library Preparation Kit (New England Biolabs) according to the manufacturer's instructions. All RNA-seq experiments were performed in biological duplicates.

### Sequencing and data analysis

The quality of the DNA samples was checked by running a High Sensitivity DNA Kit (Agilent) using Agilent 2100 Bioanalyzer before sequencing. Paired-end sequencing (41 bp reads) was performed on the NextSeq550 (Illumina) using Illumina TruSeq Read_1 and Read_2 primers. For ChIP-mini and traditional ChIP-exo data analysis, sequence reads were mapped to the reference genomes of *E. coli* K-12 MG1655 (NC_000913.3) or *S*. Typhimurium 14028s (NC_016855.1 and NC_016856.1) by bowtie with default options to generate SAM output files using ChEAP (ChIP-exo analysis pipeline), these output files were converted to BAM and GFF files for further analysis (28,29). DEOCSU (DEep-learning Optimized ChIP-exo peak calling Suite, https://github.com/SBML-Kimlab/DEOCSU) (30), a novel machine learning-based ChIP-exo peak-calling suite pipeline, was used to define binding peak candidates from the biological duplicates. To reduce false-positive peaks, those with a signal-to-noise ratio (S/N) less than 1.0 were removed. The noise level was defined as the top 5% of signals at genomic positions, as this percentage establishes a plateau in background levels and correlates well with the total read count across ChIP-exo duplicates (5–8). DeepTools2 was used to compute the correlation of sequencing libraries with the option of BAM coverage calculation to consecutive bins of equal size (10 bp) (31). Genome-scale data were visualized using GFF files via MetaScope (https://github.com/SBML-Kimlab/MetaScope) (30). For RNA-seq analysis, sequence reads were mapped onto the reference genome (NC_016855.1 and NC_016856.1) using bowtie (32) with the maximum insert size of 1,000 bp, and two maximum mismatches after trimming 3 bp at 3’ ends to generate SAM files. These output files were also converted to BAM and GFF files for further analysis. To compare overall gene expression changes during *Salmonella* infection into macrophages, featureCount and DESeq2 was used to calculate transcripts per million (TPM) value and differential expression (33). From DESeq2 output, genes with differential expression with absolute value of log_2_ fold change ≥ 1.0 and false discovery rate < 0.05 were considered differentially expressed genes (DEGs).

### DiffExo: ChIP-exo peak normalization pipeline

The DiffExo pipeline, comprising four Python and one R scripts, was developed to perform the analyses outlined in this study (Supplementary Figure S5) (https://github.com/SBML-Kimlab/DiffExo) (33,34). The Reads Per Peak per Million (RPPM) value was conceived to normalize ChIP-exo and ChIP-mini sequencing datasets. The calculation of RPPM for peak $i$ uses the following formula:


\begin{eqnarray*}RPP{M}_i\ \left( {Reads\ Per\ Peak\ per\ Million} \right) &=& \frac{{{q}_i}}{{{l}_i*\ \frac{{\mathop \sum \nolimits_j {q}_j}}{{{{10}}^6}}}} \nonumber\\ &=& \frac{{{q}_i}}{{{l}_i*\ \mathop \sum \nolimits_j {q}_j}}*\ {10}^6\end{eqnarray*}

where ${q}_i$ are raw sequencing read counts in peak $i$, ${l}_i$ is length of ChIP-exo or ChIP-mini binding peak, and $\mathop \sum \limits_j {q}_j$ corresponds to the total number of mapping reads on the reference genome. Statistically, significant differentially binding sites were calculated using DESeq2, which adopted a negative binomial distribution-based model (33). From the DESeq2 output, peaks with absolute value of log_2_ fold change ≥ 1.0 and false discovery rate < 0.05 were considered as differentially binding sites. In the RpoD datasets, differentially binding sites were labeled as differentially binding peaks (DBPs). Due to the extended length of the binding sites, these were labeled as differentially binding regions (DBRs) in the H-NS datasets.

Before applying the extra- and intracellular ChIP-mini datasets, we validated the DiffExo pipeline using less complex datasets previously generated by traditional ChIP-exo (2.4×10^10^ cells). First, changes in RpoD binding intensity in *E. coli* due to a nitrogen source shift from ammonia to unfavorable molecules (cytidine or cytosine) (8) were investigated. Among the 2,027 overlapping binding sites between ammonia and cytidine conditions, 285 positive and 214 negative DBPs were identified. Similarly, among the 2,035 overlapping binding sites between ammonia and cytosine conditions, 257 positive and 151 negative DBPs were found. These positive and negative DBPs from unfavorable nitrogen sources also exhibited significant changes in binding intensity compared to ammonia conditions (Supplementary Figure S6A) (t-test *P* < 0.05). Next, to assess whether DBPs impact transcript expression levels, we identified the target gene of each DBP and compared changes in binding intensity with corresponding changes in target gene expression levels (Supplementary Figure S6B). The changes in binding intensity and target gene expression levels displayed similar patterns, consistent with the primary function of RpoD as a positive effector. Furthermore, we also applied this pipeline to Cra of *E. coli* (6), identifying eight positive DBPs and seven negative DBPs in response to the carbon source shift from glucose to acetate. Interestingly, Cra binding of three transcription units (TUs) in their regulons (*gapA*, *nuoABCEFGHIJKLMN*, *aceBA*) reported to play an important role under acetate conditions in Kim *et al.*, were found in the positive DBPs (Supplementary Figure S6C) (6). Among these three TUs, Cra is known to directly repress the expression of *gapA*, while activating the expression of *nuoABCEFGHIJKLMN* and *aceBA*. Consistent with Cra's dual-role in transcriptional regulation, increased binding intensity was associated with significant downregulation or upregulation of target gene expression (Supplementary Figure S6C).

### Motif analysis from the ChIP-exo and ChIP-mini binding peaks

Binding motif analyses were conducted using MEME tools (parameters: -dna -revcomp -w 40 -nmotifs 2 -minsites [Number of RpoD binding sites] x 0.9 or -dna -revcomp -w 30 -nmotifs 2 -minsites [Number of RpoH binding sites]) from the MEME software suite (35). Sequences were extended by 20 bp from target genes, because only -10 box, gntAtaaT, was found without that extension. This observation conflicts with the knowledge of RpoD, because RpoD is known to specifically recognize -10 and -35 box sequences, which are expected to be covered and protected from exonuclease activity. RpoD binding peaks align well with RpoB binding peaks, and transcription start sites (TSSs) were dominantly located at the center of binding regions; thus ChIP-exo might be capturing RpoB bindings that were associated with RpoD (8) (Supplementary Figure S7).

### COG functional enrichment

H-NS DBR target genes under INT 407 intracellular conditions were categorized based on their annotated clusters of orthologous groups (COG) categories using BPGA, an ultra-fast pan-genome analysis pipeline (36). Functional enrichment of COG categories among target genes was assessed through a hypergeometric test, with a *P* < 0.05 considered statistically significant.

## Results

### Optimized ChIP-mini method generates reproducible sequencing libraries using low-input bacterial cells

To ensure compatibility with low-input bacterial cells, the traditional ChIP-exo procedure was optimized to be easily adjustable based on the initial bacterial cell numbers, a method called ChIP-mini. A comparison of our ChIP-mini method with the traditional ChIP-exo protocol, highlighting optimized steps to prevent IP-DNA loss and non-specific binding of antibodies, is presented in Supplementary Figure S1A. To mitigate IP-DNA loss, we have developed an improved strategy for the purification of IP-DNA that spans the entire process from reverse-crosslinking to sequencing library construction. The traditional ChIP-exo protocol employs PCIA and ethanol precipitation methods for IP-DNA purification after the reverse crosslinking, which is known to be one of the key steps resulting in sample loss (37). As an alternative, magnetic bead-based purification was employed to minimize IP-DNA loss and efficiently isolate only the appropriate size of IP-DNA for use in library construction. Furthermore, we devised a one-tube procedure after the second strand synthesis step, utilizing bead-based IP-DNA purification. This procedure minimizes IP-DNA sample loss and contamination by introducing the beads only once in a single tube and adjusting the PEG concentration to purify DNA of the appropriate size generated at each step. Additionally, ChIP-mini adopted optimal concentrations of antibodies and subsequent reagents based on initial bacterial cells, thereby enabling the minimization of undesirable reads in sequencing libraries. Details of these optimizations of ChIP-mini are presented in Supplementary Text S1.

**Figure 1.**
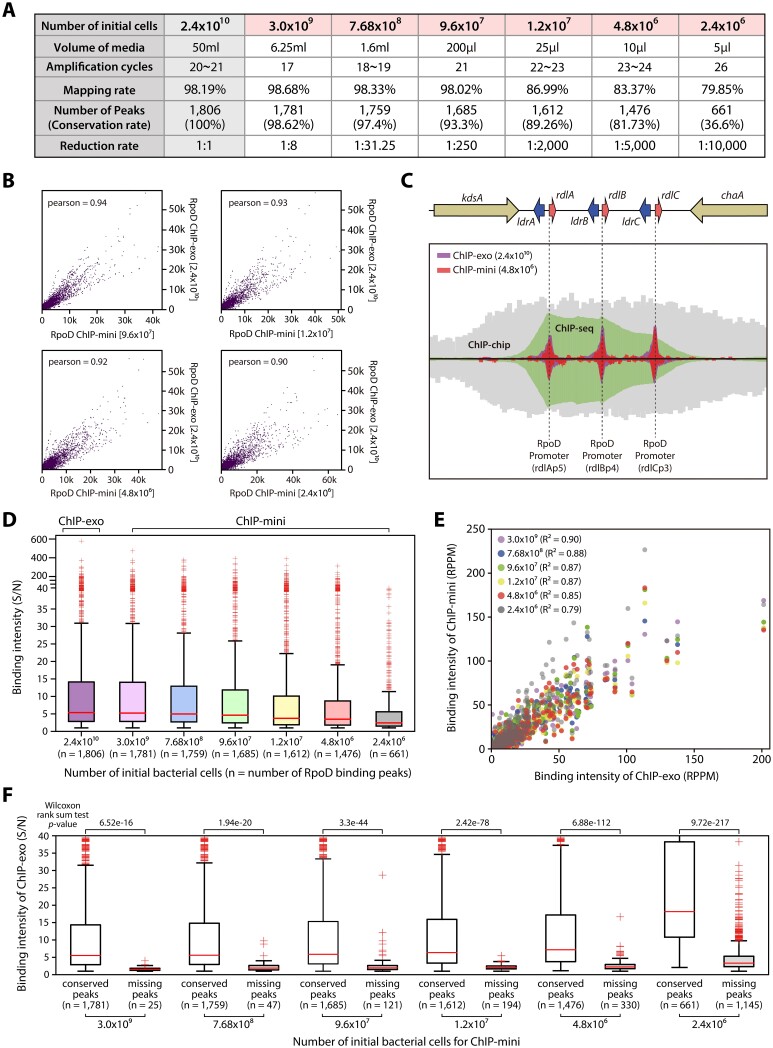
Comparative analysis of traditional ChIP-exo and enhanced ChIP-mini, preserving the advantages of ChIP-exo with a minimum input. (**A**) Assessment of mapping and conservation efficacy in ChIP-mini libraries in relation to decrease in initial bacterial cell count. (**B**) Scatter plot depicting the comparative analysis of the sequence alignment BAM files from traditional ChIP-exo and four ChIP-mini results. The read counts from each library were segmented into 10-bp bins across the *E. coli* K-12 MG1655 genome, with each dot in the plot symbolizing a specific genomic region. Abbreviation: Pearson's correlation coefficient: pearson (**C**) Evaluation ChIP-mini against three other ChIP methods for delineating TF binding profiles. ChIP-mini achieves near single base-pair resolution, even with a minimal initial quantity of bacterial cells (4.8 × 10^6^). (**D**) A boxplot illustrating the binding intensity of RpoD (S/N) across ChIP-mini and traditional ChIP-exo datasets. (**E**) A scatter plot displaying the normalized binding intensity (RPPM) of the overlapping peaks identified in both traditional ChIP-exo and each ChIP-mini dataset. (**F**) Analysis of the binding intensities of both conserved and missing peaks. In the RpoD ChIP-exo dataset, peaks missing in ChIP-mini exhibited weaker intensities (rank-sum test *P* < 0.05).

To assess the reproducibility of the ChIP-mini method, we constructed sequencing libraries for RpoD binding profiling of *E. coli* K-12 MG1655 from six samples with progressively reduced numbers of initial bacterial cells. Notably, all libraries showed comparable amplification cycles to those of traditional ChIP-exo, indicating that ChIP-mini effectively minimized the loss of IP-DNA and maintained amplification cycles consistent with traditional ChIP-exo, even with a low number of initial bacterial cells (Figure 1A). Furthermore, these ChIP-mini libraries were verified for their consistent fragment distribution (Supplementary Figure S1D), with no abnormalities observed compared to the traditional ChIP-exo library.

Amplified libraries were sequenced to a depth of 8.40 ± 1.88 million reads per ChIP-mini sample, and the nucleotide frequency at the 5’ end of the sequencing tags was compared with the traditional ChIP-exo library. Paired-end sequencing on the Illumina platform generates two types of sequencing files (Read_1.fastq and Read_2.fastq), corresponding to the positions of the sequencing primers (Supplementary Figure S2A). In the case of ChIP-seq, the DNA downstream of both sequencing primers (Read_1 and Read_2) consists of random genomic sequences due to sonication, resulting in relatively consistent nucleotide frequencies at the 5' ends of the sequencing tags. Conversely, ChIP-exo and ChIP-mini involve lambda exonuclease treatment, which digests the 5' ends of the DNA until the DNA-protein crosslink point is reached. As a result, ChIP-exo and ChIP-mini exhibit exonuclease-digested 5' ends, leading to nucleotide frequency bias in only the Read_1 file. Thus, ChIP-seq only has 5’ ends resulting from adapter ligation after sonication in both the Read_1 and Read_2 sequencing files, while ChIP-exo features an exonuclease-digested 5’ end in the Read_1 file (10) (Supplementary Figure S2B). A comparison of ChIP-exo with ChIP-mini data showed that ChIP-mini effectively maintained this ChIP-exo trait (Supplementary Figure S2C).

To determine whether ChIP-mini can maintain ChIP-exo traits at visualization levels, sequencing data of ChIP-mini was aligned to the *E. coli* genome. Pearson correlation coefficients of aligned sequencing files (10 bp-bins) between traditional ChIP-exo and each ChIP-mini consistently exceeded 0.9, even in cases with less than 9.6 × 10^7^ datasets (Figure 1B) (31). Furthermore, a comparison of ChIP-exo, ChIP-mini, and other ChIP methods demonstrated that the ChIP-mini achieves near-base pair resolution for RpoD binding sites, which is an important advantage of ChIP-exo (38) (Figure 1C). These results suggest that the optimized ChIP-mini protocol successfully minimizes IP-DNA loss, thereby constructing homogeneous libraries using a significantly smaller number of bacterial cells, up to 5,000-fold less than traditional ChIP-exo.

### Correlation between ChIP-mini and traditional ChIP-exo libraries

Visual inspection of ChIP-mini profiles at genome-wide levels demonstrated similar enrichment in libraries generated from 3.0 × 10^9^ to 2.4 × 10^6^ bacterial cells. To further evaluate the conservation of binding profiles in ChIP-mini libraries, RpoD binding peaks were identified for traditional ChIP-exo and six ChIP-mini libraries using a deep-learning based peak calling pipeline (DEOCSU) (30). While the mapping rate remained relatively consistent across all ChIP-mini cases, the peak conservation rate exhibited a significant decline in the 2.4 × 10^6^ dataset (Figure 1A). Previous studies have demonstrated that PCR amplification from limited IP-DNA results in a reduction of mappable reads and the generation of undesirable reads, such as background-mapped and unmapped reads (12,37). To assess this issue, the binding intensity of RpoD binding peaks for each ChIP-mini dataset was calculated using the S/N. As predicted, the S/N value showed a gradual decrease in the ChIP-mini datasets (Figure 1D), with a notable decline observed in datasets below the threshold of 4.8 × 10^6^. This decline was attributed to an increased noise level relative to the total number of reads (Supplementary Figure S2D). Therefore, we established 4.8 × 10^6^ as the threshold for the initial bacterial cell numbers in the ChIP-mini method. In addition, to validate the pattern of RpoD binding intensities between ChIP-exo and each ChIP-mini dataset, the binding intensity of overlapping binding peaks was normalized using RPPM. Interestingly, Pearson correlation coefficients of these normalized values revealed a strong correlation in the trend of binding intensities between each ChIP-mini dataset and the traditional ChIP-exo dataset (*R^2^* > 0.79) (Figure 1E).

Moreover, to determine whether the reduction in the initial bacterial cell counts in ChIP-mini affects the major peaks with strong intensity, the binding intensity of missing peaks in each ChIP-mini dataset was calculated with the traditional ChIP-exo dataset. Contrary to the conserved peaks, missing peaks from each ChIP-mini dataset exhibited significantly weaker binding intensities in ChIP-exo data (Figure 1F, rank-sum test *P* < 0.05), suggesting ChIP-mini can reproduce the majority of binding profiles of the traditional ChIP-exo method. Additionally, the RpoD binding site sequence motifs of ChIP-exo and ChIP-mini datasets were identified as ttgnca-15bp-gntAtaaT (Supplementary Figure S2E, lower-case characters indicate an information content <1 bit). These motifs were identical to those found in previous studies (8,21,39).

We next validated the efficiency of ChIP-mini by comparison with a ChIP-exo library constructed using 4.8 × 10^6^ bacterial cells. Due to the low concentration of recovered IP-DNA, traditional ChIP-exo required a large number of library amplification cycles (29 cycles), leading to the generation of an inappropriate library with a high concentration of adapter dimers (Supplementary Figure S3A). Furthermore, ChIP-exo libraries showed an increased proportion of unaligned read (36.3–36.7%) compared with ChIP-mini libraries (15.7–17.3%), indicating lower complexity in the sequencing libraries (Supplementary Figure S3B). Importantly, traditional ChIP-exo data did not yield sufficient coverage for genome-wide identification of binding profiles with high resolution (Supplementary Figure S3C).

In addition, we further compared our ChIP-mini method with the simplified ChIP-exo method (ChIP-exo 5.0) (10). In John *et al.*, 10–15 ml (6.4 × 10^9^–9.6 × 10^9^ cells) of *E. coli* culture in LB media (OD_600_ ≈ 0.8) under both control (30°C) and heat shock (42°C) conditions were used for ChIP-exo 5.0 (11). The alignment of these four datasets showed a relatively lower mapping rate than our ChIP-mini dataset with a similar number of initial bacterial cells (Supplementary Figure S4A and Figure 1A). Additionally, a total of 1,568 and 1,487 RpoD binding peaks were identified in both control and heat shock conditions. These number of binding peaks are similar to that observed in our ChIP-mini datasets using 9.6 × 10^6^ to 1.2 × 10^7^ initial bacterial cells (Supplementary Figure S4B). These results suggest that ChIP-mini offers an advantage when working with low bacterial cell numbers but do not necessarily indicate that it is superior to ChIP-exo 5.0.

Collectively, these results indicate that ChIP-mini surpasses traditional ChIP-exo in its capacity to efficiently generate reproducible genome-wide binding profiles from a minimal number of bacterial cells. This methodological advantage highlights the considerable potential of ChIP-mini for TRN reconstruction studies constrained by limited bacterial cell availability.

### Applications of ChIP-mini for intracellular *S*. Typhimurium infection inside macrophages

**Figure 2.**
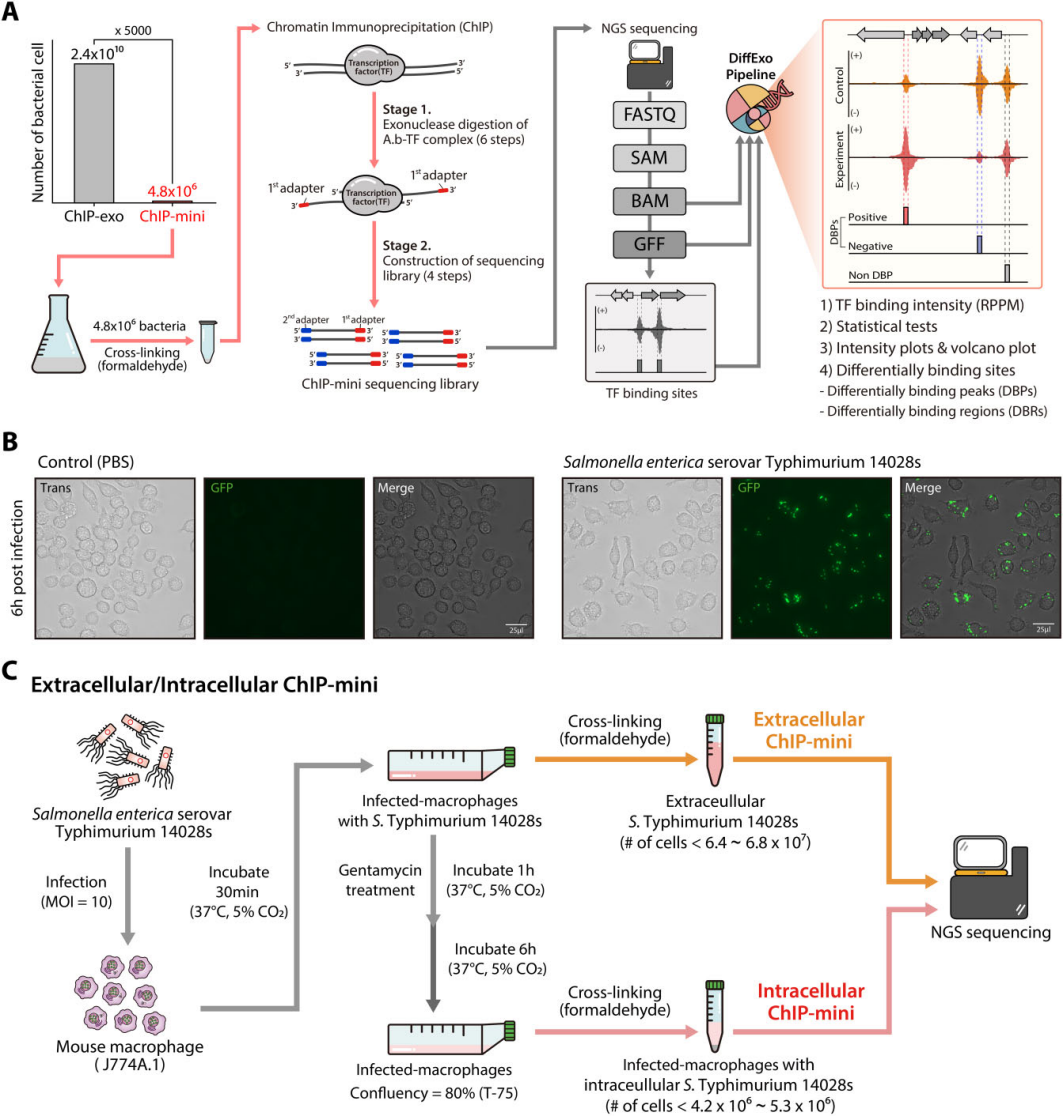
Optimized ChIP-mini method to identify binding sites of DNA-binding proteins in *S*. Typhimurium 14028s under macrophage extracellular and intracellular conditions. (**A**) Schematic diagram of the ChIP-mini workflow: this method has been optimized for use with minimal initial bacterial cells. The DiffExo analysis pipeline was utilized to normalize binding intensity and identify differential binding sites between the control and the experimental ChIP-mini datasets. (**B**) Macrophage-like cells (J774A.1) underwent infection with *S*. Typhimurium carrying pFPV25.1, which constitutively expresses GFP. PBS was used as a control. At 6 h post-infection (hpi), the infected samples were fixed, and the presence of *S*. Typhimurium were visualized through the GFP fluorescence (green). (**C**) Schematic diagram illustrating the extra- and intracellular ChIP-mini experiments with macrophage extracellular and intracellular *S*. Typhimurium.

As low-input methods are particularly useful for infection studies involving limited numbers of pathogens within host cells, we tested our method on macrophage intracellular *S*. Typhimurium. To determine whether ChIP-mini can identify changes in the binding patterns of bacterial DNA-binding regulatory proteins during host infection, macrophage-like cell line (J774A.1) and *S*. Typhimurium were employed in the infection model. Additionally, in order to statistically discern changes in binding intensity during host cell invasion, we constructed a ChIP-mini workflow incorporating the DiffExo pipeline (Figure 2A). The DiffExo pipeline was designed to perform the following tasks: (1) identification of differentially binding sites between two or more sample groups using DEseq2, (2) normalization of binding intensity, (3) statistics analysis and (4) generation of plots (Supplementary Figure S5) (33,34).

To assess infection efficiency and estimate the number of intracellular bacteria, we conducted a macrophage survival assay at 1 and 6 h post-infection (MOI of 10). Following infection, *S*. Typhimurium demonstrated an approximate infection efficiency of 38% after 1 h of infection. Additionally, *S*. Typhimurium replicated 1.38-fold within macrophages after 6 h, indicating that the CFU of intracellular *S*. Typhimurium ranges from 4.2 × 10^6^ to 5.26 × 10^6^ when converted to a 75T flask scale. This result was further validated using *S*. Typhimurium harboring the pFPV25.1 vector, which constitutively expresses GFP. This provided for direct visualization of intracellular localization of *S*. Typhimurium (Figure 2B). Moreover, the number of extracellular *S*. Typhimurium harvested after infection was identified to range from 6.41 × 10^7^ to 6.75 × 10^7^ CFU when scaled to a T75 flask. Thus, quantification of extra- and intracellular *S*. Typhimurium indicates that the number of bacterial cells under both conditions can exceed the ChIP-mini threshold (4.8 × 10^6^), allowing for the harvesting of a sufficient number of bacterial cells to perform ChIP-mini during macrophage infection experiments.

Previous studies have demonstrated that while H-NS amounts remain constant under laboratory growth conditions (40), *S*. Typhimurium decreases H-NS amounts via Lon and PhoP to activate the transcription of numerous virulence-related horizontally acquired genes during infection, facilitating successful survival within macrophages (17,41). However, genome-wide changes in H-NS binding coupled with RpoD binding profiles for elucidating transcriptional initiation mechanisms inside macrophages are still unknown. Therefore, to identify genome-wide dynamic changes in H-NS and RpoD binding profiles during macrophage invasion, we designed two types of ChIP-mini applications: extra- and intracellular ChIP-mini (Figure 2C). For these applications, *S*. Typhimurium *hns*-8myc tagged strain for H-NS and the wild-type strain for RpoD were used to infect macrophages, respectively. After infection, extracellular bacteria in DMEM were crosslinked and isolated to identify macrophage extracellular H-NS and RpoD binding profiles (extracellular ChIP-mini). At 6 h post-infection (hpi), the *S*. Typhimurium-infected macrophages were co-crosslinked. The co-crosslinked intracellular bacteria were then utilized to identify macrophage intracellular H-NS and RpoD binding profiles (intracellular ChIP-mini).

Following both ChIP-mini experiments, libraries for H-NS and RpoD under both conditions were constructed. Sequentially, these libraries were aligned to *S*. Typhimurium genome and the host genome (*Mus musculus*) to check for host nucleic acid contamination (Supplementary Figure S8A). In the extracellular libraries, the majority of sequencing reads aligned to the *S*. Typhimurium genome. However, in the intracellular libraries, host DNA contamination due to the co-crosslinking method had a more pronounced impact, resulting in a lower mapping rate compared to the extracellular libraries. Despite this lower mapping rate, approximately 2 million reads were obtained for the intracellular RpoD libraries, which is sufficient for subsequent analysis, as demonstrated in previous bacterial studies (5,6,42). To investigate whether a lower number of initial *S*. Typhimurium leads to lower library complexity, we assessed library complexity by calculating the number of uniquely mapped reads (Supplementary Figure S8B). Uniquely mapped reads represented over 87% across all aligned libraries, suggesting that high library complexity is maintained even with a limited number of initial bacterial cells. Additionally, Pearson correlation coefficients of H-NS and RpoD aligned sequencing files (10 bp-bins) between biological duplicates were consistently above 0.99 in all libraries, indicating a high level of reproducibility (Supplementary Figure S8C). Thus, the ChIP-mini applications successfully produced eight sequencing libraries for identifying genome-wide binding profiles of H-NS and RpoD in *S*. Typhimurium under both macrophage extracellular and intracellular conditions.

### Genome-wide binding profiles of H-NS under macrophage extracellular and intracellular conditions

Previously, several H-NS binding regions in *Salmonella* have been characterized through *in vitro* DNA-binding experiments and ChIP methods, such as ChIP-chip and ChIP-seq (17,43–46). However, despite the importance of H-NS in regulating the transcription of virulence genes, detailed information about the change in H-NS binding patterns and intensity during macrophage infection remains elusive. Additionally, the low sensitivity due to the broad binding regions of H-NS oligomers further complicates the identification of precise fluctuating regions during *Salmonella* infection into macrophages. To evaluate whether ChIP-exo provides a more detailed dissection of H-NS binding profiles compared to ChIP-seq, we generated H-NS ChIP-seq and ChIP-exo datasets for *S*. Typhimurium grown in M9 minimal media. A total of 538 and 674 H-NS binding regions were identified in the ChIP-seq and ChIP-exo datasets, respectively. The increased number of binding regions in ChIP-exo suggests that it offers higher resolution and sensitivity for dissecting binding profiles (Supplementary Figure S9A).

**Figure 3.**
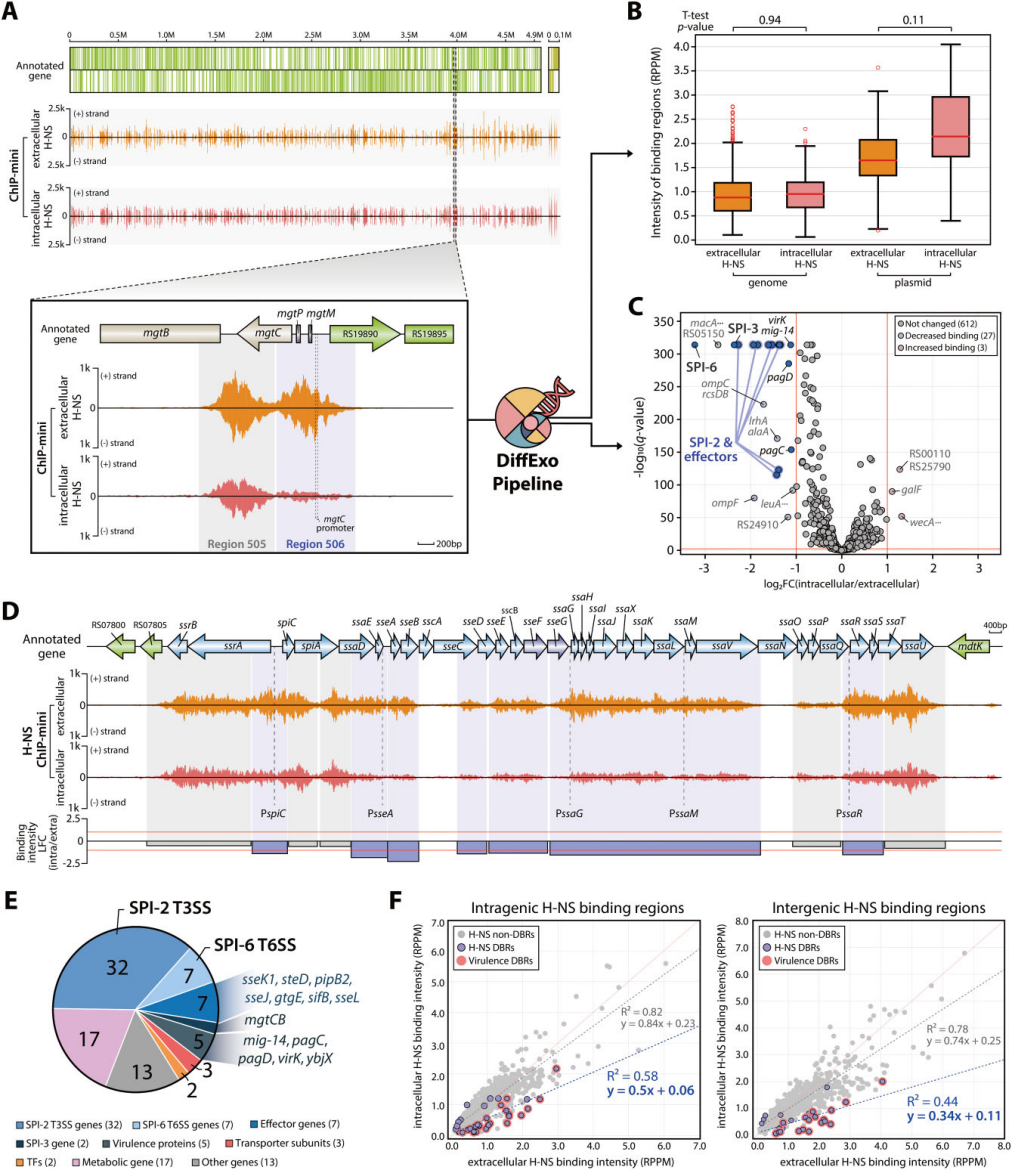
Genome-wide identification of changes in H-NS binding intensity when *S*. Typhimurium during *Salmonella* infection into macrophages. (**A**) A comprehensive landscape of H-NS binding patterns throughout the *S*. Typhimurium 14028s genome and its plasmid under macrophage extracellular to intracellular conditions. A detailed inset emphasizes a specific example of variations in H-NS binding intensity due to environmental changes. (**B**) Normalized binding intensities for H-NS binding regions under extracellular and intracellular conditions were calculated (t-test *P* > 0.05). (**C**) Volcano plot displaying H-NS differentially bound regions (DBRs) as a response to environmental changes (log_2_ fold change ≤ -1 or ≥ 1, and false discovery rate < 0.05). Blue lines indicate DBRs associated with SPI-2 and effector genes, while red font emphasizes genes related to virulence. (**D**) Variations in H-NS binding intensity within the SPI-2 region of *S*. Typhimurium. Red lines represent the threshold for DBRs, marked at -1 and 1 in binding intensity LFC. Blue boxes highlight negative DBRs, and gray boxes represent areas with no significant changes in binding (non-DBRs). In the ChIP-mini data, ‘(+)’ and ‘(−)’ indicate reads mapped onto forward and reverse strands, respectively. (**E**) A pie chart illustrating the proportion of target genes linked to both positive and negative H-NS DBRs. (**F**) The H-NS binding regions were categorized into intragenic and intergenic regions to assess the extent of impact by macrophage intracellular conditions. Pearson correlation coefficients and trend lines of H-NS binding intensities were derived from the comparative analysis of extra- and intracellular ChIP-mini datasets.

To identify H-NS binding with high resolution during infection, we employed ChIP-mini applications, which also provide improved resolution in dissecting binding regions (Supplementary Figure S9A). This allowed us to determine genome-wide binding profiles of H-NS in *S*. Typhimurium under macrophage extracellular and intracellular conditions with high resolution. From the genome-wide binding profile of H-NS ChIP-mini datasets, a total of 642 H-NS binding regions were identified under both conditions (Figure 3A and Supplementary Table S1). Consistent with previous research, these binding regions occupied 16.6% of the genome in terms of base pairs, exhibiting an average GC content of 43.7%, which is lower than that of the entire *S*. Typhimurium 14028s genome (52.1%) (17,47).

To gain further insight into the target genes associated with H-NS, the integration of H-NS binding region information with TUs annotation became necessary. Thus, the TUs with H-NS binding sites in their upstream regulatory region were chosen from the reported TU annotation (21,48). In addition, the intragenic H-NS binding region was considered to influence only that gene in which they are located. Currently, a total of 745 genes have been characterized with strong evidence as H-NS-associated genes by ChIP-chip (17). Additionally, our ChIP-seq and ChIP-exo datasets in M9 minimal media significantly expanded the number of H-NS-associated genes to 1,079 genes in 683 TUs and 1,251 genes in 799 TUs, respectively. Consistent with this result, we also identified 1,242 H-NS-associated genes in 787 TUs using the extra- and intracellular ChIP-mini datasets (Supplementary Table S1). Moreover, H-NS bindings were identified in six SPIs. In SPI-1 and SPI-2, the most representative SPIs, H-NS binding regions were divided into 16 and 12, respectively (Supplementary Figure S10). Additionally, a total of six, nine, two, and eight binding regions were identified in SPI-3, SPI-4, SPI-5 and SPI-6, respectively (Supplementary Figure S11). Furthermore, H-NS was found to bind 32 genes associated with the type III secretion system (T3SS:29) and type VI secretion system (T6SS:3) effectors (Supplementary Figure S12).

To facilitate the comparison of H-NS binding intensity between macrophages extracellular and intracellular conditions, it was imperative to confirm the homogeneity of the ChIP-mini datasets. Thus, the trend of noise level relative to the total number of reads was calculated using traditional ChIP-exo and both ChIP-mini datasets, revealing strong correlations across all datasets (*R^2^* > 0.98) (Supplementary Figure S9B). This robust correlation implies that H-NS ChIP-mini datasets were uniformly generated under both conditions, enabling reliable comparative analysis. Therefore, normalized binding intensities were calculated using the DiffExo pipeline. The overall distribution of H-NS binding intensities from both conditions was similar in both the genome and plasmid (t-test *P* > 0.05) (Figure 3B). Additionally, Pearson correlation coefficients of normalized binding intensity between both conditions showed high correlation (*R^2^* > 0.8), indicating substantial similarity in binding intensity between the two conditions (Supplementary Figure S9C). Consequently, these results demonstrate that ChIP-mini applications generated reproducible genome-wide H-NS binding regions of *S*. Typhimurium under both conditions. In addition, the high similarity between the extra- and intracellular ChIP-mini datasets raises the question of whether there are regions where the intensity of H-NS binding significantly differs.

### H-NS binding intensity significantly decreases in SPI-2 and effector genes after infection

To determine the DBRs of H-NS, we conducted a comparative analysis using the DiffExo pipeline. Overall, a total of 30 DBRs were identified within macrophages (Supplementary Table S2, absolute value of log_2_ intensity fold change ≥ 1.0 and false discovery rate < 0.05). Of these, 27 regions were classified as negative DBRs, demonstrating a significant decrease in binding intensity, while three DBRs were classified as positive DBRs, exhibiting increased binding intensity. Additionally, the changes in binding intensities were more significant in the negative DBRs (t-test *P* < 0.05), indicating that decreases in binding intensity were more pronounced than increases within macrophage (Supplementary Figure S9D). Furthermore, our analysis revealed that 21 out of 27 negative DBRs are associated with virulence genes, highlighting the importance of H-NS binding for the virulence of *S*. Typhimurium within macrophages (Figure 3C). Notably, a significant reduction in H-NS binding intensity was particularly evident in SPI-2 but not SPI-1, and these negative DBRs included all TSSs of SPI-2 that have been previously reported (49) (Figure 3D). Additionally, negative DBRs were also found, with one each located in the intergenic regions of SPI-3 and SPI-6 (Supplementary Figure S11).

The target genes associated with these 30 DBRs encompassed 88 target genes, with about 60.2% identified as virulence-related genes (Figure 3E and Supplementary Table S3). These target genes included the entirety of SPI-2 genes, a portion of SPI-3 and SPI-6 genes. In SPI-3, a negative DBR in SPI-3 was found upstream of *mgtCB* transcriptional unit (Supplementary Figure S11), genes that promote *Salmonella* survival within macrophages (25,26). Additionally, a negative DBR was located in the promoter region of *tagKJ*-*tssEFGA* and *tssH* transcriptional units in SPI-6. Moreover, seven effector genes (*sseK1*, *sseJ*, *pipB2*, *gtgE*, *steD*, *sseL* and *sifB*) were identified among the negative DBR target genes (Supplementary Figure S13). Additionally, a substantial decrease in H-NS binding intensity was also detected in three other important virulence genes (*mig-14*, *pagC* and *virK*) (50–52).

Furthermore, to identify which genomic regions exhibited a significant decrease in binding intensity, we divided H-NS binding regions into intragenic and intergenic categories and compared the changes in binding intensity (Figure 3F). This analysis revealed that the reduction in H-NS binding intensity was more pronounced in intergenic regions. Altogether, these findings suggest that a reduction in H-NS binding at the TSS regions may increase the accessibility of sigma factors or other TFs, facilitating the transcription initiation of virulence genes under macrophage intracellular conditions.

Additionally, we generated ChIP-mini datasets from overnight-grown LB cultures (3.0 × 10^9^ cells) before the infection experiments, to facilitate comparative analysis with extracellular datasets. From these datasets, a total of 664 H-NS binding regions were identified in the stationary phase under LB media conditions. A comparative analysis between LB stationary and extracellular conditions identified a substantial number of 251 DBRs (75 negative DBRs and 176 positive DBRs). Among these DBRs, 42 were associated with a total of 82 virulence-related genes. Notably, H-NS binding in SPI-1, SPI-2, SPI-4, *ybjX*, and *hns* showed a significant increase before infection (Supplementary Figure S14). In addition, a reduction in H-NS binding was observed across the majority of SPI effector genes, as well as in SPI-6 and the O-antigen biosynthesis cluster (Supplementary Figure S14). These results suggest that the increase in H-NS binding is particularly pronounced in its association with major SPI regions before macrophage entry, indicating that H-NS may also play an important regulatory role in preparing for infection.

### Dynamic changes in RpoD binding profiles during *Salmonella* infection into macrophages

To ascertain whether the reduced binding of H-NS during infection into macrophages induces transcription initiation, we identified the binding sites of RpoD under both conditions using ChIP-mini applications. These ChIP-mini datasets showed a high correlation in the trend of noise level relative to the total number of reads across all ChIP-mini and traditional ChIP-exo datasets (*R^2^* > 0.98), indicating the robustness of the data for further analysis (Supplementary Figure S15A).

**Figure 4.**
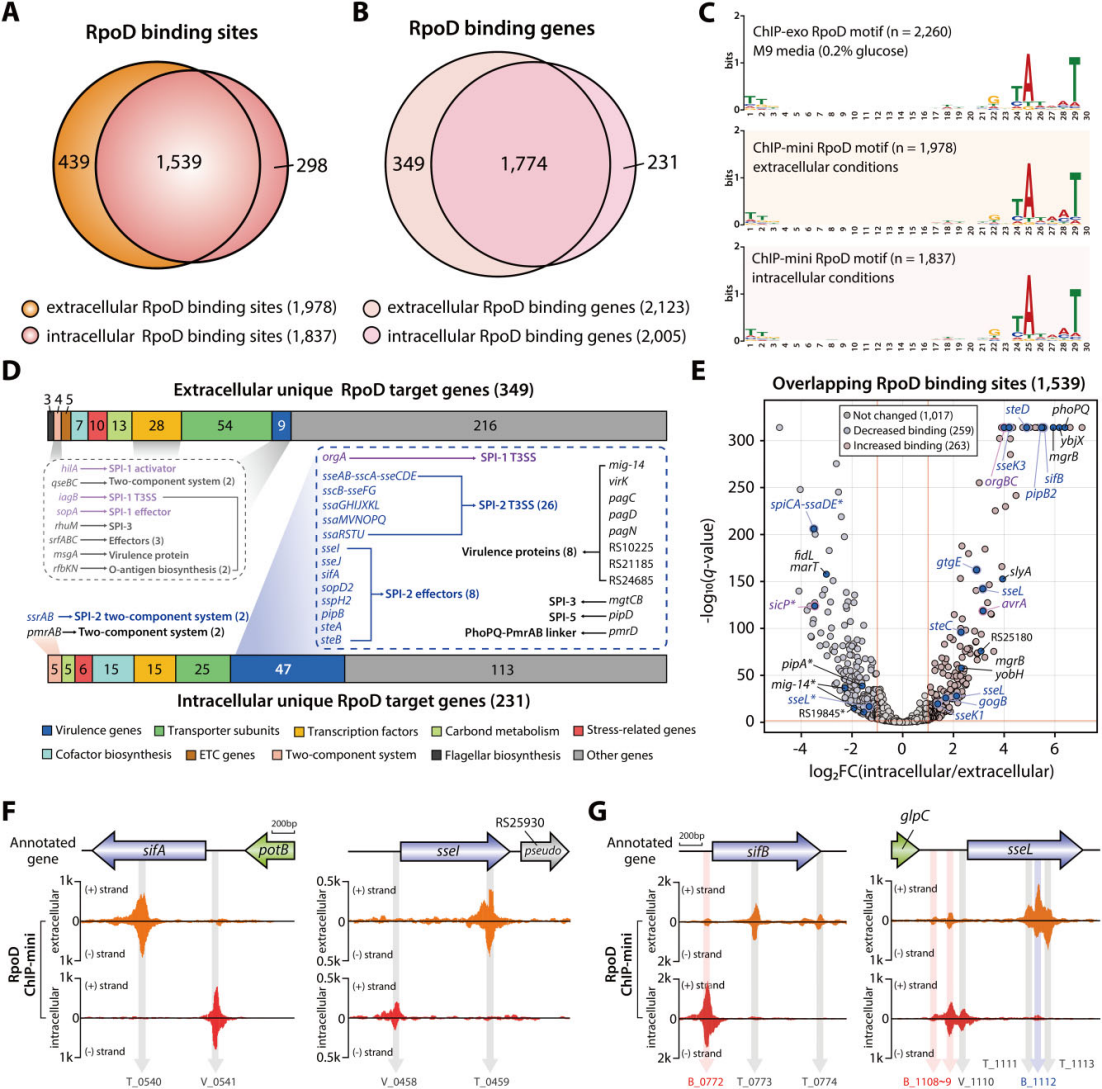
Genome-wide identification of changes in RpoD binding site and intensity during *Salmonella* infection into macrophages. (**A**) Analysis of the overlapping RpoD binding sites between macrophages extracellular to intracellular conditions. (**B**) Comparison of RpoD target genes identified by ChIP-mini under macrophage conditions, both conditions. (**C**) Analysis of Motifs in RpoD binding sites identified from both extra- and intracellular ChIP-mini datasets. A traditional ChIP-exo dataset generated under M9 minimal media condition served as a control. (**D**) Unique RpoD target genes specific to each condition (unique RpoD target genes refer to those identified exclusively in either extra- or intracellular conditions). Genes associated with SPI-1 are highlighted in purple font, while those linked to SPI-2 are indicated in blue font. (**E**) Volcano plot displaying RpoD differential binding peaks (DBPs) triggered by environmental changes (log_2_ fold change ≤ -1 or ≥ 1, and false discovery rate < 0.05). Notably, RpoD positive DBP genes include nine SPI-2 effectors (*sifB, sseK1*, *sseK3*, *sseL, steC*, *steD*, *gogB*, *gtgE* and *pipB2*), three SPI-1 genes (*orgBC* and *avrA*), two TCSs (*phoPQ*), one TF (*slyA*), and four virulence proteins (*mgrB*, *yobH*, *ybjX* and STM14_RS25180). Genes related to SPI-2 are marked in blue font, and those pertaining to SPI-1 in purple font. Asterisks highlight intragenic (non-regulatory) binding of RpoD, indicating binding occurring downstream of the regulatory region. (**F**) The transition of RpoD binding sites on effector genes from intragenic to intergenic region in response to environmental changes. (**G**) The change in RpoD binding intensity for effector genes. “V_peak number” signifies unique RpoD binding sites found in intracellular conditions, “T_peak number” refers to unique RpoD binding sites found in extracellular conditions, and “B_peak number” represents common RpoD binding sites across both conditions. Negative DBPs are denoted in blue font, and positive DBPs are indicated in red font.

A total of 1,978 and 1,837 RpoD binding sites were identified under macrophage extracellular and intracellular conditions, respectively (Figure 4A and Supplementary Table S1). The majority of these binding sites overlapped, and macrophage intracellular RpoD binding sites in the genome exhibited higher intensity compared to extracellular conditions (Supplementary Figure S15B, rank-sum test *P* < 0.05). Integrating TU information with RpoD binding sites in their upstream regulatory regions, we identified 1,404 TUs with 2,123 genes and 1,306 TUs with 2,005 genes in each ChIP-mini dataset (Figure 4B). The sequence motifs derived from RpoD binding sites were also found to be “ttgaca-15bp-gntAtaaT,” consistent with previous findings (Figure 4C, lower-case characters indicate an information content <1 bit) (9).

During *Salmonella* infection into macrophages, RpoD binding sites for 349 genes disappeared, of which 12 virulence-related genes were identified (Figure 4D and Supplementary Table S4). These genes included the SPI-1 invasion protein *iagB*, the SPI-1 key regulator *hilA*, the HilA-mediated SPI-1 effector *sopA*, and the SPI-3 virulence protein *rhuM* (53,54). Additionally, *qseBC* two-component system (TCS) genes that regulate SPI-1 and SPI-2 genes involved in bacterial invasion and survival in host cells were found only under extracellular conditions (55). The remaining six genes (*srfABC*, *msgA* and *rfbKN*) were associated with virulence effectors, virulence proteins, and O-antigen biosynthesis, respectively.

Under macrophage intracellular conditions, 231 genes with intracellular unique RpoD binding sites were identified, including 51 genes related to virulence (Figure 4D). Among these virulence-related genes were all SPI-2 and eight SPI-2 effector genes, except for *spiCA*-*ssaDE*, which exhibited RpoD binding sites under both conditions. These virulence-related genes also include four TCS genes encoding the master regulator of the SPI-2 regulon (*ssrAB*) and the PhoPQ-mediated TCS (*pmrAB*) (56–58). Additionally, four SPI genes (SPI-1:1, SPI-3:2, and SPI-5:1), eight other virulence-related genes, and PhoPQ-PmrAB linker protein gene (*pmrD*) were exclusively identified within macrophages.

To further explore change in RpoD binding intensity during infection into macrophages, the normalized binding intensities of overlapping peaks were compared, revealing a biphasic pattern, with one subset showing strong correlation and another displaying low correlation (Supplementary Figure S15C). This suggests that a subset of RpoD binding peaks undergoes changes within macrophages. Consequently, to identify RpoD binding peaks with statistically significant differences in intensity between extra- and intracellular conditions, a comparative analysis of DBPs was performed. A total of 263 positive DBPs and 259 negative DBPs were identified within the 1,539 overlapping binding sites (Supplementary Figure S15D). A significant increase in RpoD binding intensity was observed upstream of various virulence-related genes, including nine SPI-2 effectors, three SPI-1 genes, two TCSs, one TF and four virulence proteins (Figure 4E). Conversely, RpoD binding decreased in the intragenic regions of nine virulence-related genes and the intergenic regions of two SPI-3 genes (*fidL* and *marT*).

Another intriguing finding related to SPI-2 effectors is that RpoD binding peaks were found in the upstream region of 20 out of 29 SPI-2 effector genes under macrophage intracellular conditions (Table 1). Excluding *sseK2*, 19 genes were associated with intracellular unique RpoD binding and positive DBPs. According to Jennings *et al.* (59), these SPI-2 effector genes were categorized into various functions crucial under host intracellular conditions (Supplementary Figure S16). Interestingly, differential preferences were observed among effectors within the same category. In the actin cytoskeleton category, RpoD binding was observed upstream of *steC*, while no binding was detected in *spvB*. Additionally, five effectors related to innate immune signaling were associated with RpoD binding, including *sseK1*, *sseK2*, *sseK3*, *gogB* and *sspH2*. However, no binding was found in *spvCD*, *sspH1*, *gtgA*, *pipA* and *gogA*.

**Table 1.** RpoD binding sites associated with SPI-2 T3SS effectors

Effector	Binding conditions	LFC	Left end	Right end	Peak ID	Interaction Partner(s)
**SCV membrane dynamics**						
*sifA*	intracellular		1,269,069	1,269,118	Peak_0541	PLEKHM1, PLEKHM2, GDP-RhoA
*sopD2*	intracellular		1,012,313	1,012,361	Peak_0410	Rab7, Rab32*
*sseJ*	intracellular		1,731,215	1,731,266	Peak_0788	GTP-RhoA, Cholesterol
*gtgE*	both	2.91	1,101,621	1,101,670	Peak_0462	Rab29*, Rab32*, Rab38*
*steA*	intracellular		1,680,127	1,680,181	Peak_0759	PI(4)P
*pipB2*	both	5.47	2,948,185	2,948,234	Peak_1322	Kinesin-1
**Golgi network association**						
*sseF*	intracellular		1,493,233	1,493,287	Peak_0670	SseG, ACBD3
*sseG*	intracellular		1,493,233	1,493,287	Peak_0670	SseF, ACBD3
**Adaptive immunity**						
*sseI*	intracellular		1,098,254	1,098,303	Peak_0458	IQGAP1
*steD*	both	4.89	2,285,132	2,285,183	Peak_1037	mMHCII, MARCH8
**Actin cytoskeleton**						
*steC*	both	2.3	1,802,216	1,802,265	Peak_0833	MEK1*, HSP27*
*spvB*	ND		ND	ND	ND	G-actin*
**Innate immune signaling**						
*sseK1*	*both*	1.37	4,388,686	4,388,737	Peak_2007	FADD*, TRADD*
*sseK1*	intracellular		4,388,877	4,388,926	Peak_2008	FADD*, TRADD*
*sseK2*	intracellular		2,282,663	2,282,714	Peak_1031	ND
*sseK2*	both		2,282,772	2,282,823	Peak_1032	ND
*sseK2*	intracellular		2,283,038	2,283,089	Peak_1033	ND
*sseK3*	both	4.2	2,094,308	2,094,361	Peak_0990	TRIM32, TRADD*
*gogB*	both	1.7	2,782,050	2,782,099	Peak_1248	SKP1, FBXO22
*spvC*	ND		ND	ND	ND	p-ERK*, p-p38*, p-JNK*
*spvD*	ND		ND	ND	ND	XPO2
*sspH1*	ND		ND	ND	ND	PKN1*
*sspH2*	intracellular		2,394,972	2,395,021	Peak_1089	UbcH5-Ubiquitin, SGT1, NOD1*
*gtgA*	ND		ND	ND	ND	p65*, RelB*
*pipA*	ND		ND	ND	ND	p65*, RelB*
*gogA*	ND		ND	ND	ND	p65*, RelB*
**Unknown function**						
*sifB*	both	5.54	1,702,090	1,702,144	Peak_0772	ND
*steB*	intracellular		1,729,393	1,729,444	Peak_0785	ND
*sseL*	both	2.17	2,445,959	2,446,008	Peak_1108	OSBP, Ubiquitin
*sseL*	both	3.16	2,446,103	2,446,152	Peak_1109	OSBP, Ubiquitin
*pipB*	intracellular		1,135,870	1,135,919	Peak_0482	ND
*srfJ*	ND		ND	ND	ND	ND
*slrP*	ND		ND	ND	ND	ERdj3, TRX1*

Extra- and intracellular RpoD binding profiles upstream of SPI-2 effector genes, as classified by Jennings *et al.* (59). Binding condition refers to instances where RpoD binding is detected in the macrophage intracellular environment, the extracellular environment, or in both conditions (ND: Not determined). LFC indicates the log_2_ fold change in binding intensity between extracellular and intracellular RpoD binding, calculated using the DiffExo pipeline (false discovery rate < 0.05). Interaction partners refer to host proteins that interact with SPI-2 effectors of *S*. Typhimurium, with substrates denoted by asterisks.

Furthermore, fluctuations in RpoD binding, transitioning from intragenic to intergenic regions, were observed in SPI-2 effector genes. RpoD binding sites of *sifA* and *sseI* shifted from the intragenic region to the TSS after infection (Figure 4F). Additionally, the RpoD binding intensity at the TSS of *sifB* and *sseL* significantly increased, while intragenic RpoD binding either disappeared or decreased in intensity (Figure 4G). Thus, these fluctuations suggest that RpoD may be recruited to the appropriate location for transcription initiation by other TFs under macrophage intracellular conditions.

In addition, a total of 2,097 RpoD binding sites and 2,233 corresponding target genes were identified under LB stationary conditions (Supplementary Figure S17A). Of these, 76.6% (1,606/2,097) of the binding sites and 80.2% (1,791/2,233) of the target genes overlapped with those observed under extracellular conditions. Moreover, the emergence of new virulence-related genes was confirmed before macrophage entry, with five genes (*msgA*, *srfBC*, *spvA* and *srbB*) exclusively exhibiting RpoD binding under extracellular conditions (Supplementary Figure S17B). Concurrently, a relatively large reduction in RpoD binding sites associated with 22 virulence-related genes was also observed. These genes include those within SPI-1 (*hilC*), SPI-2 (*ssaGHIJXKL*), SPI-4 (*siiEF*), SPI-6 (*tssMF*-RS02025-RS02030), SPI-1 effectors (*sopF*, *sopE2*), SPI-2 effectors (*pipB*, *sspH1*, *sseK1*) and additional virulence proteins (*mgtC*, RS10225, *pmrD*) (Supplementary Figure S17B).

### The fluctuating binding profiles of H-NS and RpoD overlap at the TSS region of virulence genes under macrophage intracellular conditions

**Figure 5.**
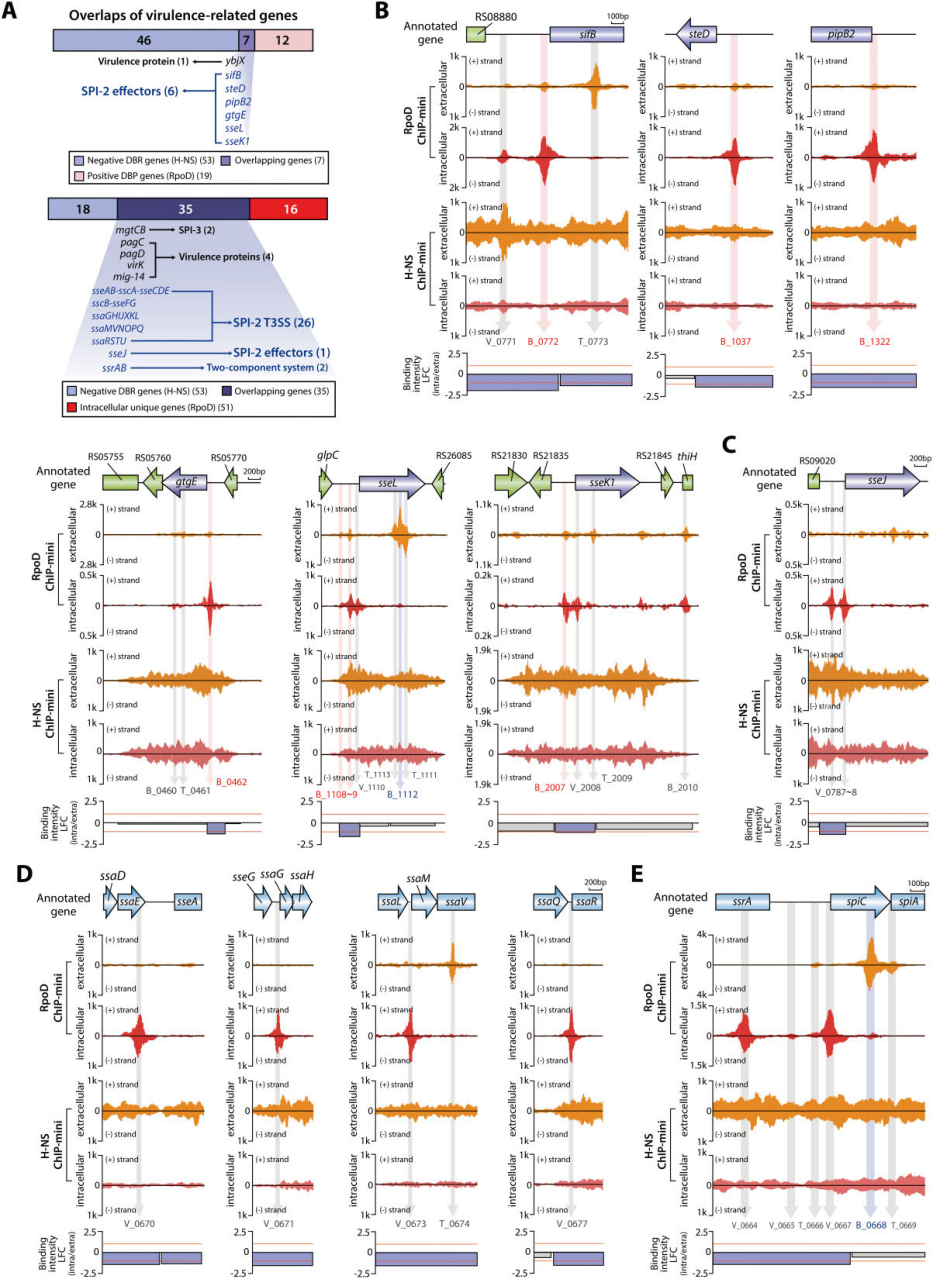
Fluctuations in RpoD binding sites on virulence-associated genes impacted by H-NS negative DBRs. (**A**) The intersection of virulence-related genes among RpoD target genes and H-NS negative DBR genes in macrophage intracellular conditions. The upper bar represents the overlap between H-NS negative DBR genes and RpoD positive DBP genes. The lower bar illustrates the overlap between H-NS negative DBR genes and intracellular unique RpoD target genes. (**B**) Identification of six effector genes as common elements between RpoD positive DBP genes and H-NS negative DBR genes. (**C**) The gene *sseJ* is recognized as a shared element of intracellular unique RpoD genes and H-NS negative DBR genes. (**D**) Discovery of four intracellular unique RpoD binding sites in the SPI-2 region influenced by H-NS negative DBRs. (**E**) Analysis of H-NS negative DBR with complex RpoD binding sites in SPI-2. The *spiCA-ssaDE* TU exhibits a single extracellular unique RpoD binding site (T_0666) and two intracellular unique RpoD binding sites at TSS (V_0665, V_0667).

As discussed above, we observed fluctuations in H-NS and RpoD binding profiles of *S*. Typhimurium within macrophages. This observation suggests that the reduction in H-NS binding during *Salmonella* infection into macrophages induces an increase in RpoD binding. To explore this premise, we integrated binding profiles of H-NS and RpoD to identify overlapping binding regions associated with virulence (Figure 5A). In regions where H-NS binding was diminished, a significant increase in RpoD binding intensity was observed at the TSS of six SPI-2 effectors (Figure 5B) and a single virulence protein (Supplementary Figure S18A).

Furthermore, intracellular unique RpoD binding sites upstream of virulence-related genes were identified in H-NS negative DBRs. These binding sites were associated with a single effector gene (*sseJ*) (Figure 5C) and 26 SPI genes (Figure 5D). Notably, four RpoD binding sites in SPI-2 were consistent with previously reported SPI-2 promoter sites (49). Moreover, intracellular unique binding sites also were detected at the TSS of SPI-3 TU (*mgtCB*) and four virulence protein genes (Supplementary Figure S18B). The intergenic region of *spiCA-ssaDE* and *ssrAB* showed complex RpoD binding patterns (Figure 5E). Within this complex binding pattern, weak intracellular unique binding for *ssrAB* (V_0665) and a shift of binding from the intragenic to the intergenic region for *spiCA-ssaDE* were observed, suggesting that RpoD predominantly binds to both TUs under macrophages intracellular conditions. Additionally, strong binding in the *ssrA* intragenic region (V_0664) is associated with *spiCA-ssaDE* (49).

**Figure 6.**
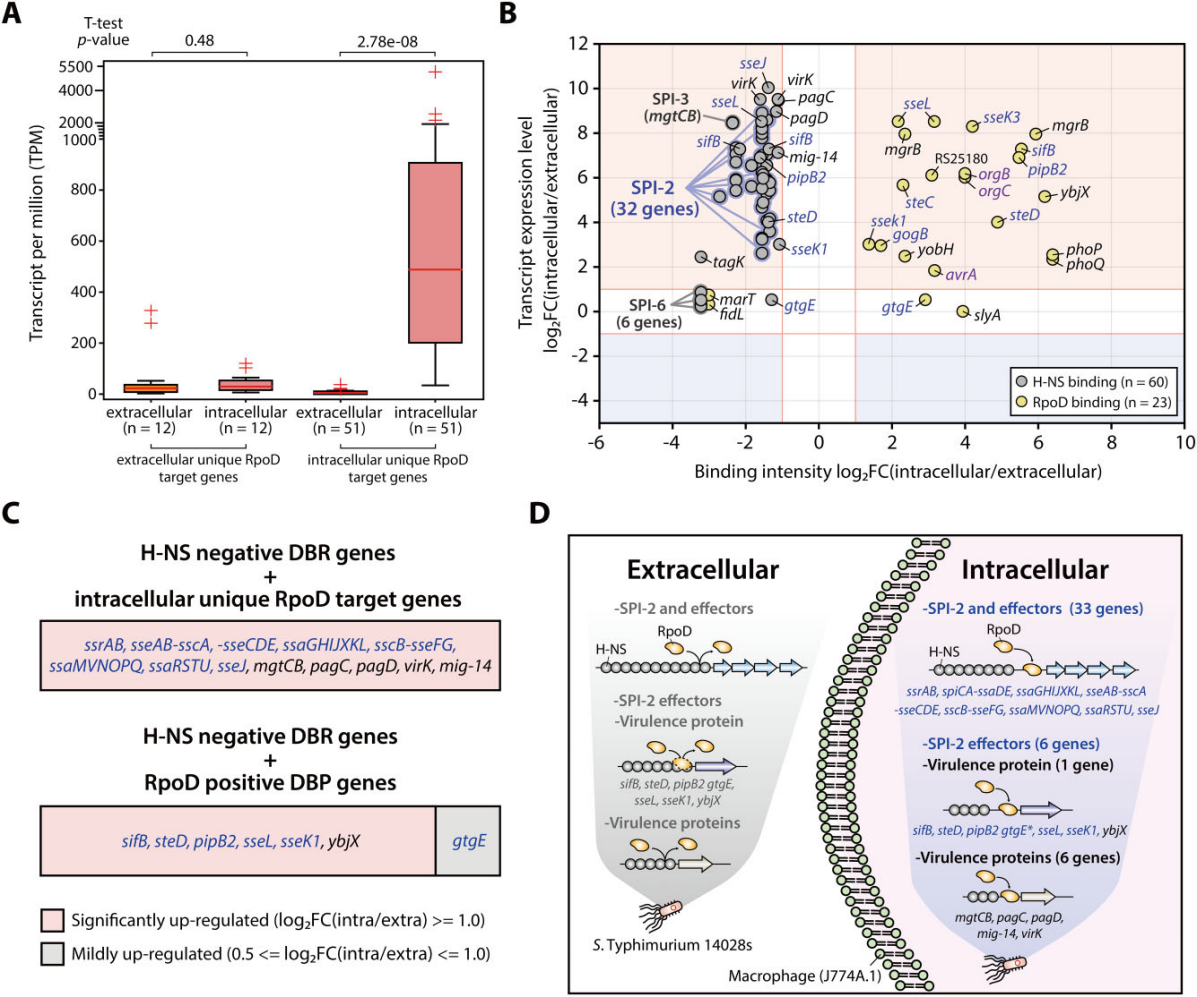
Comprehensive analysis of H-NS and RpoD interaction in regulating transcription initiation of virulence-related genes under macrophage intracellular conditions. (**A**) Box plots illustrate changes in the expression levels of virulence-related genes corresponding to RpoD binding unique to macrophage extracellular and intracellular conditions. The expression of RpoD target genes uniquely identified within macrophage was found to be significantly upregulated (t-test *P* < 0.05). (**B**) The scatter plot depicts the correlation between the change in binding intensities of H-NS and RpoD with corresponding virulence-related gene expression levels during *Salmonella* infection into macrophages. Genes related to SPI-2 are marked in blue, and those pertaining to SPI-1 in purple. (**C**) An increase in RpoD binding at H-NS negative DBRs induces transcription initiation of virulence-related genes, consequently leading to a significant up-regulation in their transcriptional expression. (**D**) Overview highlights how the binding dynamics of H-NS and RpoD on virulence-related genes in *S*. Typhimurium 14028s modulate transcription initiation in response to the environmental transition from macrophage extracellular to intracellular conditions.

Finally, to determine a causal relationship between the fluctuation of H-NS and RpoD binding profiles and the alteration in transcript levels, RNA-seq experiments were performed for macrophage extracellular and intracellular *S*. Typhimurium. A total of 1,960 DEGs (absolute value of log_2_ fold change ≥ 1.0 and a false discovery rate < 0.05) were observed by comparing the transcript expression levels of *S*. Typhimurium before and after infection, with 910 genes up-regulated and 1,050 genes down-regulated (Supplementary Figure S19A and Supplementary Table S5). Surprisingly, expression of virulence-related RpoD target genes exclusively found inside macrophages showed significant up-regulation (t-test *P* < 0.05) (Figure 6A). Furthermore, a decrease of H-NS binding and an increase of RpoD binding were found to lead to a notable up-regulation in the expression of their target genes (Figure 6B). Additionally, expression of SPI-2 effector genes related to novel or increase of RpoD binding was also found to have more prominent increases in expression levels than other effector genes (Supplementary Figure S19B). These findings also highlight our premise that a reduction in H-NS binding within macrophages induces RpoD binding, facilitating the transcription initiation of virulence genes (Figure 6C). Consequently, under macrophage extracellular conditions, we determined that H-NS binds tightly, restricting the access of RpoD for initiation of transcription in virulence-related genes. Conversely, macrophage intracellular conditions lead to a significant reduction in H-NS binding at the TSS region, thereby enhancing the accessibility of RpoD results in novel binding events and increased binding intensity to overcome the silencing effect for transcription initiation (Figure 6D).

### Extended applications of ChIP-mini at various post-infection times or in different host cells

**Figure 7.**
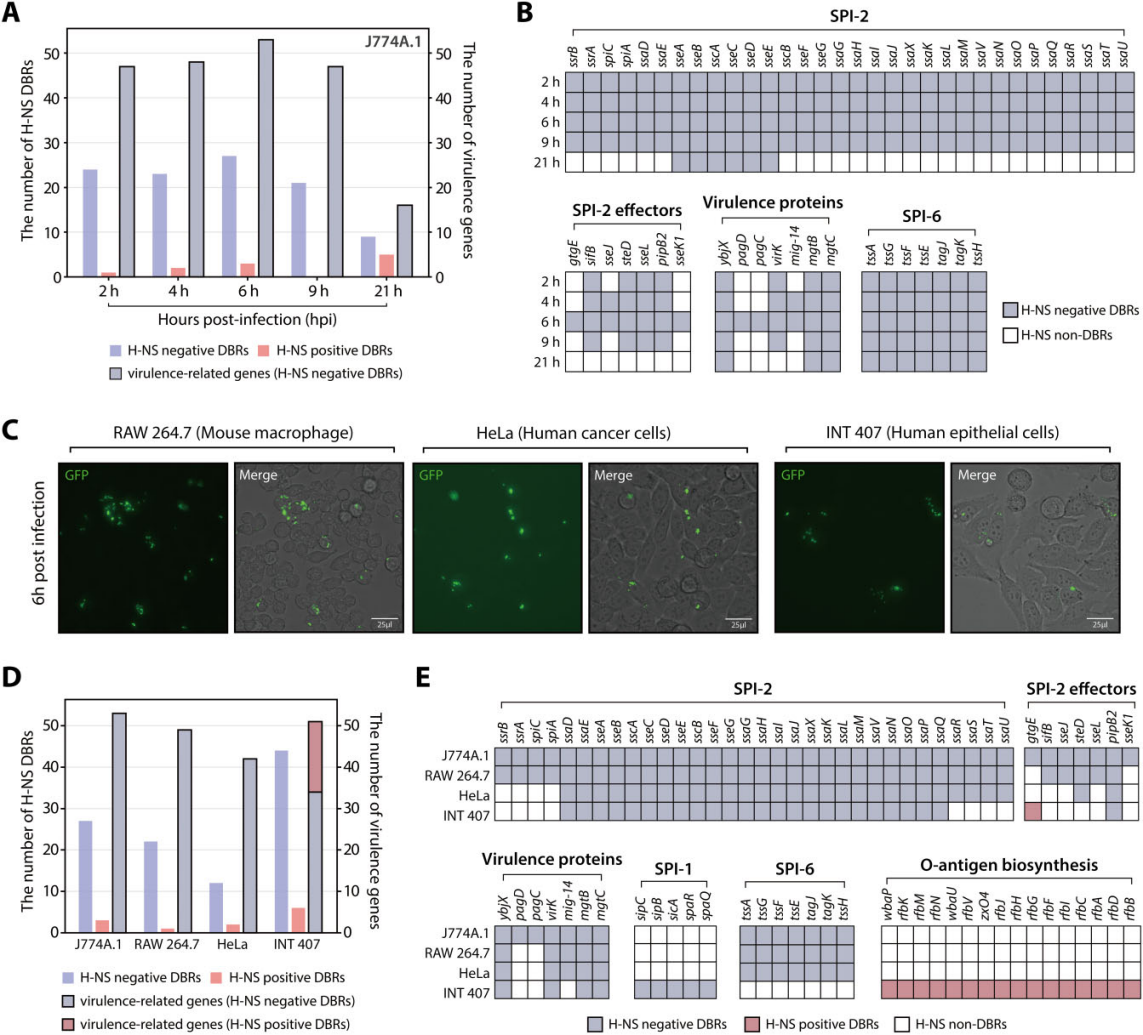
The ChIP-mini workflow enables the identification of changes in genome-wide H-NS binding patterns at various post-infection time points or in different host cells. (**A**) Bar chart illustrating the number of H-NS DBRs and DBR-associated virulence-related genes at different post-infection time points. Each time-point dataset was compared to extracellular datasets using the DiffExo pipeline for analysis. (**B**) Heat maps displaying H-NS DBRs for virulence-related genes across various post-infection time points. Blue boxes indicate negative DBRs, while white boxes denote non-DBRs. (**C**) Three types of host cells were infected with *S*. Typhimurium carrying the pFPV25.1 vector, which constitutively expresses GFP. PBS was used as a control. At 6 h post-infection (hpi), the infected samples were fixed, and *S*. Typhimurium presence was visualized through GFP fluorescence (green). (**D**) Bar chart displaying the number of H-NS DBRs and DBR-associated virulence-related genes in four types of host cells. (**E**) Heat maps showing H-NS DBRs for virulence-related genes in four types of host cells after infection. Blue boxes represent negative DBRs, red boxes indicate positive DBRs, and white boxes represent non-DBRs.

To further investigate the dynamic nature of H-NS-DNA interactions during infection, we conducted additional intracellular ChIP-mini at four post-infection time points (2, 4, 9 and 21 h). From these intracellular ChIP-mini datasets, a total of 642 H-NS binding regions were identified under four-time points, consistent with the results at 6 h. To determine the DBRs of H-NS at each time point, we performed a comparative analysis utilizing datasets from extracellular and four different infection time points. Overall, 25, 25, 21 and 14 DBRs, along with 47, 48, 47 and 16 virulence-related genes, were identified at the four respective time points (Figure 7A and Supplementary Table S6). In SPI-2, H-NS binding intensity significantly decreased under intracellular conditions at all time points except 21 h (Figure 7B). This reduction was more pronounced during the early infection phase (2–6 h) and was nearly abolished at the late infection phase (21 h) (Supplementary Figure S20). Moreover, H-NS binding associated with SPI-2 effectors and virulence protein genes showed a marked reduction at 6 h, while no SPI-2 effector genes were identified as DBR target genes at 21 h. These dynamic changes in H-NS binding indicate that H-NS undergoes the most active alterations at 6 h post-infection, with H-NS binding gradually recovering in the hours following. Thus, these results suggest that ChIP-mini is capable of capturing both static snapshots and the dynamic patterns of DNA-binding proteins across different post-infection time points.

Furthermore, whether ChIP-mini can work in different types of host cells, we conducted extra- and intracellular ChIP-mini using three host cell lines (mouse macrophage: RAW 264.7, human epithelial cells: HeLa, INT 407). To assess the infection capability of *S*. Typhimurium in different types of host cells, we performed infection experiments using *S*. Typhimurium harboring the pFPV25.1 vector. Following infection, it was confirmed that *S*. Typhimurium successfully invades three types of host cells, demonstrating that they can be used to validate intracellular ChIP-mini (Figure 7C). Using these host cells, we generated 12 extra- and intracellular ChIP-mini datasets, including biological duplicates. From these ChIP-mini datasets, a total of 642 H-NS binding regions were also identified across the three host cell types, consistent with findings in J774A.1 cells. As a result of the comparative analysis between extra- and intracellular conditions, a total of 23, 14 and 50 DBRs, along with 49, 42 and 51 virulence-related genes, were identified in RAW 264.7, HeLa and INT 407 cells, respectively (Figure 7D and Supplementary Table S7). Changes in H-NS binding in RAW 264.7 cells showed similar patterns to those in J774A.1, while the two types of epithelial cells demonstrated distinctly different H-NS binding changes (Figure 7E and Supplementary Figure S21). First, in SPI-2 and SPI-2 effector-associated H-NS bindings, a relatively small number of DBRs were identified within epithelial cells compared to macrophages (Figure 7E). Second, decreased H-NS binding in the SPI-1 was exclusively identified within INT 407 (Supplementary Figure S21). Third, virulence genes associated with positive DBRs were also exclusively observed under INT 407 intracellular conditions, encompassing those in the O-antigen biosynthesis cluster and the SPI-2 effector *gtgE*. (Supplementary Figure S21). Fourth, the number of DBRs and their target genes was significantly higher in INT 407 cells compared to the other three host cell types (Supplementary Table S7).

These results raise the question of which genes are associated with significant changes in H-NS binding during infection of INT 407. Therefore, functional analysis using COGs categories was conducted to reveal the functional enrichment of DBR target genes. It was found that the majority of genes were enriched in cell wall/membrane/envelope biogenesis (M), cell motility (N) and intracellular trafficking/secretion vesicular transport (U) (Supplementary Figure S22). This enrichment suggests that H-NS may play a role not only in the regulation of virulence genes but also in the control of genes involved in cellular structure and motility functions within INT 407 cells. Hence, these results indicate the possibility of different H-NS binding mechanisms depending on host cell type, as well as the potential for ChIP-mini workflow to be applied across various host cell types.

## Discussion

In this work, we optimized the traditional ChIP-exo method to overcome previous challenges in identifying binding profiles of DNA-binding proteins with a near single-base pair resolution for intracellular bacteria. ChIP-mini demonstrates considerable improvements over other ChIP-based methods in identifying binding profiles at genome-wide levels, even when using a minimal initial quantity of bacterial cells (4.8 × 10^6^). These improvements are attributed to its enhanced DNA recovery efficiency through a one-tube enzymatic procedure, along with the optimized use of sonication and reagents. Furthermore, ChIP-mini demonstrates nearly complete conservation and resolution of RpoD binding profiles utilizing 4.8 × 10^6^*E. coli* cells (>83%), which the traditional ChIP-exo method did not maintain.

Elucidating the complex TRNs of host-infected pathogens is not only crucial for understanding the transcriptional regulation of virulence genes, but also has the potential to lead to the development of new antibiotics or anti-virulence therapeutics for intracellular pathogens (60). Recent studies have extensively investigated the transcript expression profiling of *S*. Typhimurium under host intracellular and infection-relevant *in vitro* growth conditions (49,61,62). However, obtaining high-resolution binding profiles to investigate the genome-wide binding dynamics of DNA-binding proteins in intracellular pathogens remains challenging, due to the inherently limited quantity of intracellular bacteria recoverable from infection experiments. This limitation necessitated the use of optimized ChIP-mini to directly measure the impact of the environmental shifts on the binding of DNA-binding proteins with improved resolution.

For *S*. Typhimurium, a ubiquitous and clinically significant intestinal pathogen, we utilized the ChIP-mini workflow to reveal the crucial interaction between H-NS and RpoD concerning virulence genes during macrophage infection. The reduction in H-NS binding in intergenic regions exposes the promoter, leading to (1) novel bindings of RpoD to the TSS of SPI-2, SPI-2 effector, SPI-3 and virulence protein genes and (2) an increase in RpoD binding to SPI-2 effectors and virulence protein genes (Figure 6D). Remarkably, these findings are consistent with significantly upregulated expression levels of those genes, highlighting a crucial regulatory mechanism whereby diminished H-NS binding enables enhanced RpoD binding to induce transcription initiation of key virulence genes after infection. By doing so, *S*. Typhimurium ensures the effective activation of transcription initiation of virulence genes for proliferation and replication when it is inside macrophages. In addition to virulence-related genes, ChIP-mini data also identified RpoD target genes exhibiting increased binding inside macrophages, which were implicated in various metabolic processes (Figure 4D). Thus, our integrated ChIP-mini data and transcription profiles not only provide genome-wide binding information of H-NS and RpoD but also offer a valuable resource for future studies to elucidate complex metabolic perturbations in *S*. Typhimurium during infection.

In *S*. Typhimurium, SPI-1 and SPI-2 are crucial for the invasion of eukaryotic cells and for facilitating replication to cause systemic disease within host cells, respectively (63). However, the T3SS proteins of SPI-1 are recognized by macrophages, triggering a caspase-1-mediated form of inflammatory cell death known as pyroptosis (64). Therefore, the repression of SPI-1 is crucial under both macrophage extracellular and intracellular conditions, while SPI-2 activation is specifically essential for the replication and proliferation of *S*. Typhimurium within macrophages (63). Consistent with previous studies, our H-NS intracellular ChIP-mini demonstrated notable reductions in SPI-2 and seven SPI-2 effectors but not SPI-1. Moreover, six out of seven SPI-2 effector genes have been reported to play a vital role in *Salmonella* virulence within host cells. SseK1 inhibits TNFα-induced NF-kB signaling of innate immune signaling (65). SseJ and PipB2 contribute to the formation and elongation of *Salmonella*-induced tubular (SITs), which are tubular extensions of the *Salmonella*-containing vacuole (SCV) (59,66–68). GtgE is a cysteine protease that prevents the accumulation of Rab29, Rab32 and Rab38 on SCV and SITs (69,70). In addition, SteD is a transmembrane effector required for the depletion of surface mature major histocompatibility class II (mMHCII), thereby inhibiting antigen presentation and T-cell proliferation within dendritic cells (71). Presumably, SseL functions as a deubiquitinase, preventing the accumulation of lipid droplets and inhibiting the clearance of cytosolic aggregates, leading to late macrophage cell death (72–74). The remaining effector, SifB, is a core effector always found in intestinal serovars, suggesting its significance in *Salmonella* virulence, although its precise function within macrophages remains elusive (59).

Furthermore, changes in RpoD binding during infection revealed differential preferences among SPI-2 effectors within the same category (Supplementary Figure S16). This result suggests the possible existence of a subgroup of SPI-2 effector genes that may play a critical role in adapting to macrophage intracellular conditions. Additionally, we also found a key finding from a transcriptional regulation standpoint. Under macrophage intracellular conditions, novel and increased RpoD binding was observed upstream of transcriptional regulatory cascade genes governing *Salmonella* virulence. This cascade includes three positive DBP genes (*phoPQ* and *slyA*) and five intracellular unique target genes (*ssrAB*, *pmrAB* and *pmrD*), which have been reported to activate the expression of SPI-2 effectors and LPS modification genes to enhance survival and virulence (Supplementary Figure S23) (58,75–79).

In the previous study, the number of H-NS molecules per cell decreases by Lon protease when *S*. Typhimurium enters to macrophages (41). However, ChIP-based methods have difficulty detecting this overall degradation pattern of DNA-binding proteins, as these techniques are generally more suited for identifying specific changes in protein-DNA interactions rather than capturing global proteolysis events. This difficulty arises from the ChIP and library amplification (PCR enrichment) steps in ChIP-based methods. In the case where H-NS affects all loci (Supplementary Figure S24A), (1) total H-NS in *S*. Typhimurium is degraded by proteolysis under intracellular conditions, leading to a reduction in the overall levels of H-NS-DNA complexes, and (2) this reduction in H-NS-DNA complexes affects the ChIP step, resulting in a lower recovery of IP-DNA. However, this IP-DNA may exhibit a similar binding pattern to that observed under extracellular conditions due to non-specific proteolysis of total H-NS proteins. Therefore, this overall degradation pattern is compensated for PCR enrichment during library amplification and normalization in the DiffExo pipeline, resulting in the categorization of non-DBRs. In the case where H-NS affects specific loci (Supplementary Figure S24A), 1) antisilencing proteins (such as PhoP, SlyA, HilD and SsrB) and RpoD occupy the H-NS binding regions, resulting in a reduction of H-NS binding in these specific regions, and (2) this localized reduction of H-NS binding is more significant than the overall reduction caused by proteolysis and can be easily detected as a differential pattern using the ChIP method. Moreover, PCR amplification and normalization steps cannot fully compensate for the localized reduction in H-NS binding, resulting in the categorization of negative DBRs.

Additionally, we identified an antisilencing protein, PhoP, with increased RpoD binding and transcript expression within macrophages. This regulator is known to alleviate H-NS silencing and recruit RNA polymerase to horizontally acquired genes (80). Notably, activated PhoP binding sites were mainly found upstream of the RpoD binding sites in macrophage intracellular *S*. Typhimurium, acting as transcriptional activators (Supplementary Figure S24B). Moreover, the majority of PhoP regulon genes were also identified among intracellular RpoD target genes (Supplementary Figure S24C). This observation suggests a complex regulatory network involving RpoD and antisilencing proteins, which proactively interact with H-NS binding regions within macrophages (45,80,81). Thus, future efforts to reveal genome-wide interactions of these transcriptional regulators with H-NS and RpoD using ChIP-mini applications would greatly expand our understanding of the comprehensive regulatory networks responding to macrophage intracellular conditions.

Using ChIP-mini, we are able to identify the binding dynamics of H-NS and RpoD with a near-base pair resolution during the transition from macrophage extracellular and intracellular conditions, highlighting the crucial interaction between H-NS and RpoD at transcriptional initiation standpoint. Incidentally, we confirmed the feasibility of adapting ChIP-mini applications to other three host cells (RAW 264.7, HeLa and INT 407), successfully identifying changes in genome-wide binding of H-NS during infection. Hence, the ChIP-mini workflow will be a powerful tool to expedite the study of TRN reconstruction in a wide range of intracellular pathogens, requiring only minor modifications to the experimental process described herein.

However, limitations regarding host DNA contamination and the large number of enzymatic steps involved in the experimental process remain challenges when applying ChIP-mini to intracellular pathogens. Notably, host DNA contamination caused by the co-crosslinking step is thought to increase experimental costs by necessitating a large number of sequencing reads for the analysis of intracellular ChIP-mini libraries. To address this limitation, immunomagnetic separation (IMS) has been evaluated to isolate intracellular pathogens from host cell lysates (36,82) (Supplementary Figure S25A and Supplementary methods). (1) An antibody with specificity for *Salmonella* LPS was utilized to prepare an antibody-magnetic bead complex. (2) Intracellular *Salmonella* was isolated from the lysate of co-crosslinked macrophages using magnetic beads. (3) The *Salmonella*-captured beads were immediately subjected to sonication, followed by magnetic separation to remove the beads. Using these sonicated samples, we generated two ChIP-mini libraries for *S*. Typhimurium-infected macrophages (J774A.1) at the 2-h post-infection time point. Interestingly, ChIP-mini combined with IMS showed a 1.57-fold increase in sequencing reads mapped to *S*. Typhimurium, indicating an effective reduction of host DNA contamination (Supplementary Figure S25B). In this study, we developed ChIP-mini applications based on traditional ChIP-exo for bacteria (ChIP-exo 1.0), which is the most widely used method for bacterial TRN studies. However, this method involves numerous processes, comprising 13 enzymatic steps, to generate sequencing libraries. To mitigate this limitation, we have undertaken the optimization of ChIP-exo 5.0, a recently developed method designed to significantly reduce the number of enzymatic steps (10). In future studies, optimizing a simplified ChIP-exo method combined with IMS could enable the generation of high-quality libraries that minimize host DNA contamination and streamline the experimental process.

In addition, another limitation of our ChIP-mini method is that its applicability to intracellular DNA-binding proteins with low abundance or few binding sites has not yet been determined. Although this study did not test intracellular ChIP-mini on these DNA-binding proteins, we have applied ChIP-mini to RpoH, a sigma factor with few binding sites, using *E. coli* (9.6 × 10^7^ cells) under *in vitro* conditions. These ChIP-mini libraries exhibited ChIP-exo characteristics in sequencing libraries (Supplementary Figure S26A) and demonstrated high Pearson correlation coefficients in aligned sequencing files (10 bp bins) compared to traditional ChIP-exo and ChIP-mini datasets (Supplementary Figure S26B). Notably, ChIP-mini also conserved 96.8% of RpoH binding profiles, indicating its applicability to DNA-binding proteins with a low number of binding sites (Supplementary Figure S26C). However, these DNA-binding proteins may recover lower concentrations of IP-DNA compared to abundant DNA-binding proteins, leading to increased susceptibility to host DNA contamination. Therefore, optimizing the ChIP-mini method to minimize host DNA contamination, as mentioned above, is anticipated to be a key factor in identifying binding profiles of DNA-binding proteins with low abundance or few binding sites.

Finally, microbial cultures are often heterogeneous in gene expression and phenotypic changes (49,83). In this study, we optimized traditional ChIP-exo protocols using exponential-phase *E. coli* cultures, which approximate a homogeneous population for protocol development. Using this established protocol, we demonstrated that we could map overall binding patterns of H-NS during *Salmonella* infection into host epithelial or macrophage cell lines. However, the natural variability in physiological states of *S*. Typhimurium inside macrophages could arise from bacterial killing, efficiency of intracellular replication, and heterogamous responses to host cellular physiology; these conditions may introduce additional heterogeneity. Further refinement through the combination of single-cell-level sequencing techniques will need to be addressed to resolve this issue in future research (84).

## Supplementary Material

gkaf009_Supplemental_Files

## Data Availability

The whole dataset of ChIP-mini and RNA-seq has been deposited to GEO with the accession number of GSE252525 and GSE270061, respectively. DiffExo pipeline is freely available at the public GitHub repository (https://github.com/SBML-Kimlab/DiffExo) and Zenodo at https://doi.org/10.5281/zenodo.14552245.
