# Supplementary material for: ChIP-mini: a low-input ChIP-exo protocol for elucidating DNA-binding protein dynamics in intracellular pathogens
Source: Nucleic Acids Res. 2025 Jan 27;53(3):gkaf009. doi: 10.1093/nar/gkaf009 (PMC11770342; doi:10.1093/nar/gkaf009)
Supplement: gkaf009_Supplemental_Files [file gkaf009_supplemental_files.zip › Supplementary Information.docx]

**Supplementary Information**

**Supplementary Text S1**

To generate a ChIP-exo library from a low number of bacterial cells, ChIP-mini was optimized based on the traditional ChIP-exo method (1-4). Traditional ChIP-exo and ChIP-mini share the same procedures consisting of four parts as follows: 1) DNA fragmentation, 2) exonuclease digestion of antibody-TF complex (Stage 1), 3) reverse crosslinking, and 4) construction of sequencing library (Stage 2) (Figure S1A).

Incomplete fragmentation causes high background in sequencing data due to long DNA fragments or results in low-quality libraries for sequencing from short DNA fragments. Therefore, optimization of the sonication procedure is the first step in reducing the initial bacterial cell number for traditional ChIP-exo. For the sonication procedure in the ChIP-mini, we adopted the indirect sonication method, which homogeneously transmits ultrasonic energy through water to multiple samples. This method is especially effective for small sample volumes, avoiding sample loss and eliminating potential sources of contamination. To make the indirect sonication method compatible with a low number of initial bacterial cells, we optimized both the volume of the fragmentation buffer and the sonication duration for different numbers of crosslinked *Escherichia coli* K-12 MG1655 cells. Given that traditional ChIP-exo for bacteria typically utilizes DNA fragments with starting lengths ranging from 200 to 600 bp, this optimization process aimed to produce a consistent size distribution even when using reduced numbers of initial bacterial cells. The 3.0x10^9^ and 7.68x10^8^ samples were fragmented by 25 minutes of sonication (50s on and 10s off intervals and amplitude 50%), utilizing 272.75 µl and 218.2 µl of fragmentation buffer, respectively. For the remaining four samples, 40 minutes of sonication (50s on and 10s off intervals and amplitude 50%) with 218.2 µl of fragmentation buffer was used. It was observed that the efficiency of sonication decreased when less than 180 µl of the total buffer volume was used. This modification enabled the generation of targeted fragment distribution even with small numbers of bacterial cells (Figure S1B).

Following the optimization of the sonication process, antibodies and reagents for the subsequent steps in ChIP-mini were also modified to maintain traditional ChIP-exo traits and avoid non-specific binding of antibodies. Compared to the traditional ChIP-exo method, a 75% reduction in the volume of the 1^st^ antibody and Dynabeads was found to be the optimal concentration for generating adequate sequencing libraries with low initial bacterial cell numbers (Figure S1C). Additionally, a 90% reduction in the subsequent reagents required for Stage 1, including lambda exonuclease digestion and primary adapter ligation, was also found to be optimal. For the 3.0x10^9^ sample, the volumes of 1^st^ antibody, Dynabeads, and Stage 1 reagents were reduced to 50%, 50%, and 25%, respectively compared to traditional ChIP-exo.

In the case of ChIP-seq, the reverse crosslinking step involving DNA purification is known to be one of the key steps resulting in immunoprecipitated-DNA (IP-DNA) loss (5). ChIP-mini is more susceptible to IP-DNA loss caused by reverse crosslinking because it uses a minimal number of bacterial cells. Furthermore, this loss of IP-DNA can further hinder not only Stage 2 procedures but also generate amplification bias due to the low concentration of IP-DNA. Thus, it was considered a crucial point to improve the efficiency of IP-DNA purification when using a low number of initial bacterial cells. According to traditional ChIP-exo, Phenol:Chloroform:Isoamyl Alcohol (PCIA) and ethanol precipitation methods are used to purify the IP-DNA after the reverse crosslinking step. These experimental procedures present challenges in purifying low-concentration IP-DNA from reverse crosslinking solutions and often result in non-selective isolation, yielding inappropriately sized IP-DNA fragments unsuitable for sequencing library amplification. Moreover, these methods are time-intensive, typically requiring over two hours for IP-DNA isolation. To address these limitations, we adopted a bead-based purification method that simultaneously minimizes IP-DNA loss, provides size selectivity, and significantly reduces processing time to less than 20 minutes.

Finally, to retain more IP-DNA and optimize the experimental materials for Stage 2 steps when constructing the sequencing library, we developed a one-tube procedure for DNA purification to minimize the loss of IP-DNA samples by using a bead-based purification. This procedure utilizes an enzyme mixture from each step as a bead-elution buffer, introduces the beads only once, and adjusts the polyethylene glycol (PEG) concentration to purify IP-DNA of the appropriate size generated at each step. Additionally, the bead cleanup procedure between the dA-tailing and the 2^nd^ adapter ligation was omitted by using enzyme heat inactivation (6). This simplified method enables an average reduction of 52% in the volume of reagents required compared to traditional ChIP-exo and minimizes IP-DNA loss to multiple column-based purifications. Consequently, the number of amplification cycles needed for library amplification showed no significant difference between the 9.6x10^7^ ChIP-mini sample and the traditional ChIP-exo sample using 2.4x10^10^ bacterial cells.

**Supplementary Figures**

**
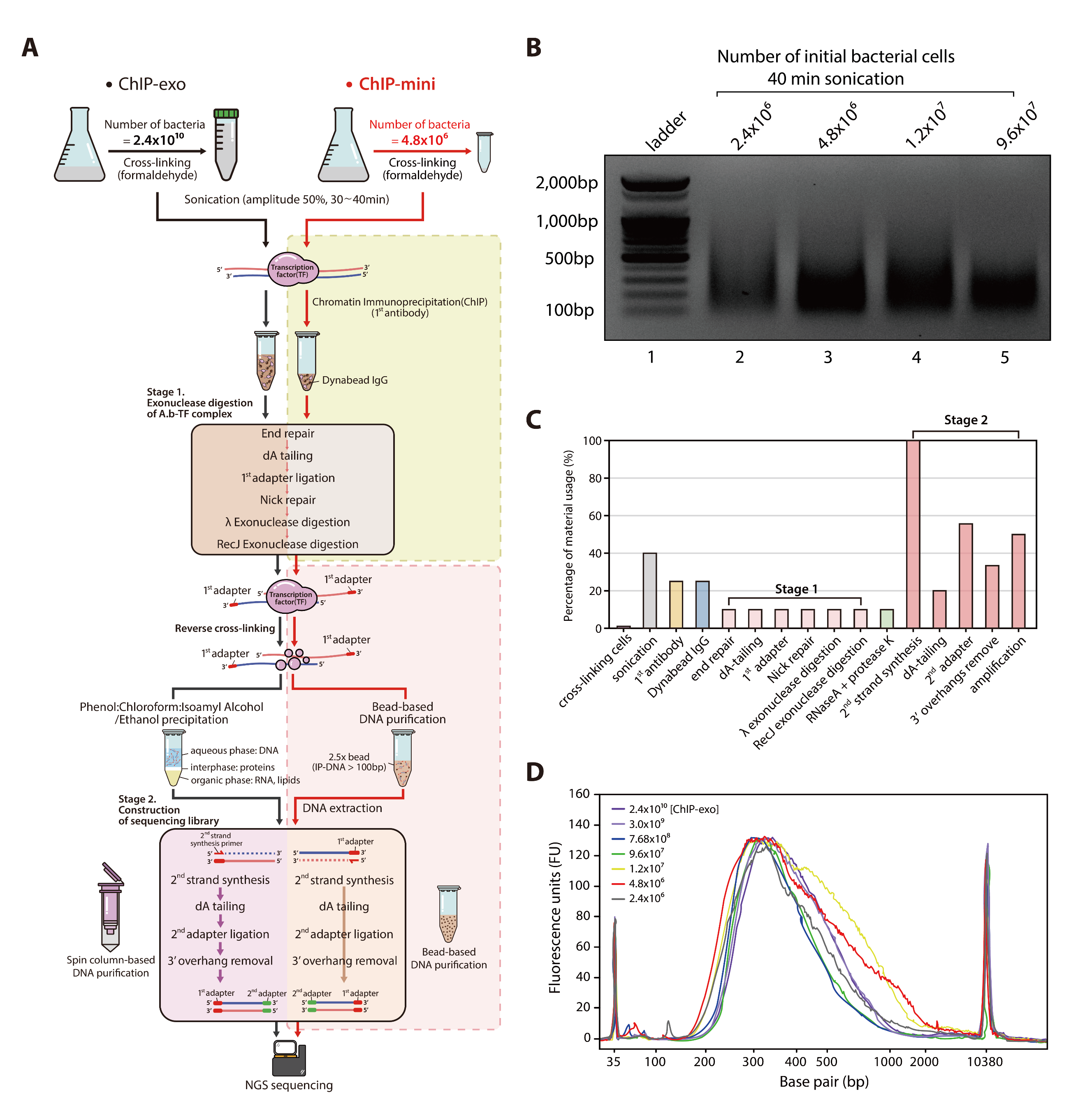
**

**Figure S1. Comparison of the traditional ChIP-exo and optimized ChIP-mini processes.** (A) Comparison of steps to generate ChIP-exo and ChIP-mini libraries. Different numbers of initial bacterial cells are lysed and sonicated to perform ChIP. The optimized volume of the ChIP process enables minimization of reagents for 1^st^ adapter ligation and exonuclease digestion of IP-DNA including antibody-TF complex (Stage 1). After reverse crosslinking, ChIP-mini uses 2.5x magnetic beads to extract IP-DNA over 100 bp. Additionally, ChIP-mini utilizes a bead-based DNA purification rather than a spin column-based one, allowing for reduced reagent volumes during sequencing library construction (Stage 2). Yellow box: steps optimized to reduce non-specific binding via antibody and subsequent reagents; orange box: steps optimized to minimize IP-DNA loss. (B) The distribution of DNA fragment sizes based on a low number of bacterial cells in 1% agarose gel after 40 minutes of sonication (< 9.6x10^7^). Sonicated DNA was fragmented to the proper size to proceed with ChIP-mini (200~600 bp). (C) Detailed experimental material usage for the minimum number of crosslinked cells in ChIP-mini. (D) Distribution of ChIP-exo and ChIP-mini libraries for *E. coli* RpoD after PCR amplification. The size distribution of ChIP-mini libraries was similar to that of the libraries generated with the original ChIP-exo method.

**
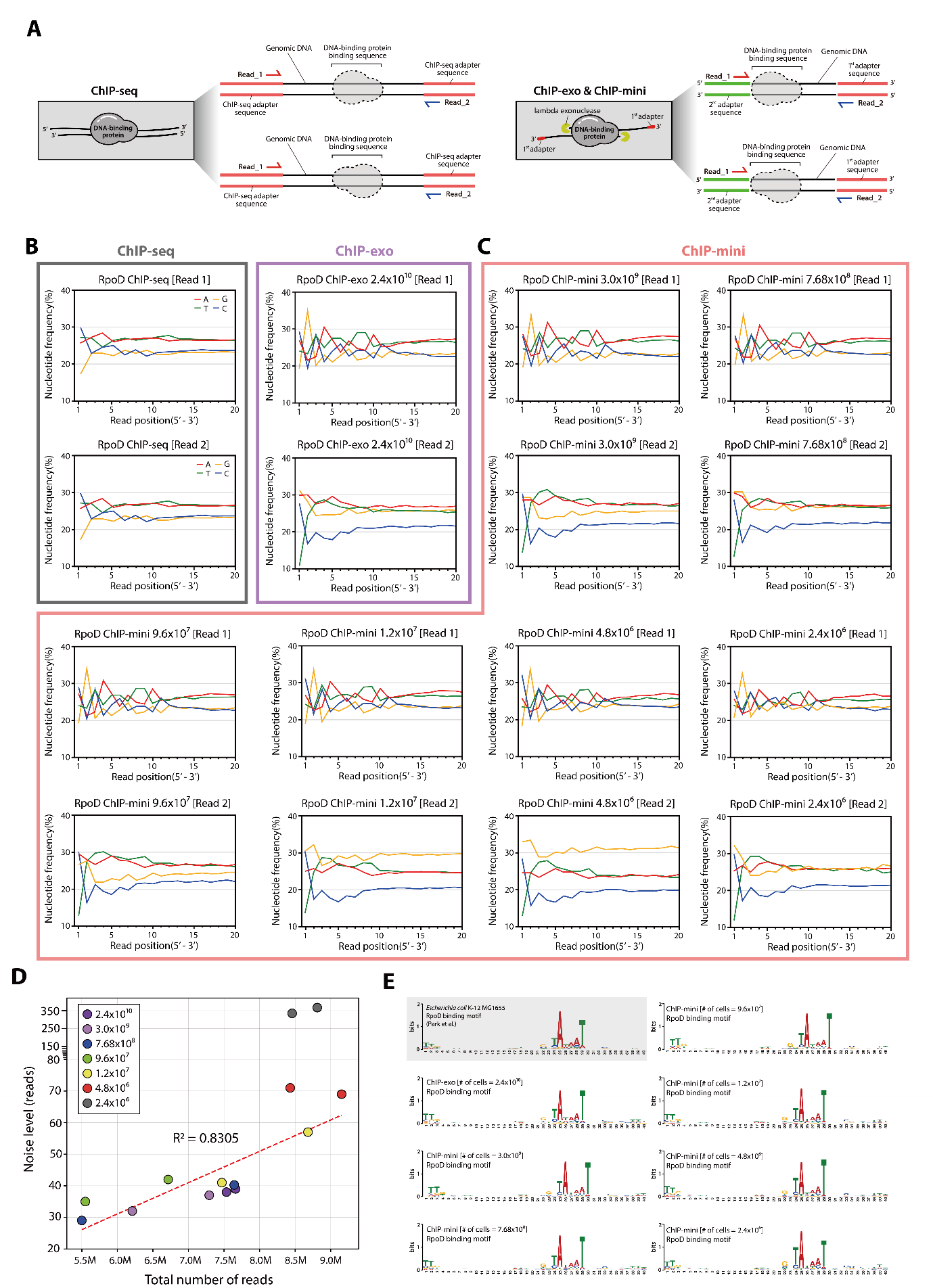
**

**Figure S2. Comparison of *E. coli* RpoD ChIP-mini datasets according to different number of initial cells.** (A) Schematic diagram illustrating the different nucleotide frequencies at the 5' ends of ChIP-seq and ChIP-exo datasets. (B) Nucleotide frequency at the 5’ end of the paired-end sequencing reads for ChIP-seq and ChIP-exo libraries. In the ChIP-seq library, read_1 and read_2 exhibit adapter ligation after dA-tailing of sonicated ends, while ChIP-exo library show the product of the exonuclease-digested 5’ end in Read_1 file. (C) Nucleotide frequency at the 5’ end of the paired-end sequencing reads for ChIP-mini libraries. Read_1 files of ChIP-mini libraries show the product of the exonuclease-digested 5’ end, which is a trait of a ChIP-exo library. (D) Determination of noise level in ChIP-exo and ChIP-mini sequencing reads. Noise level was correlated with the total number of reads until reaching 4.8x10^6^ initial bacterial cells. (E) Motif analysis of RpoD binding sites was performed on traditional ChIP-exo and each ChIP-mini dataset, resulting in identical sequence motifs.


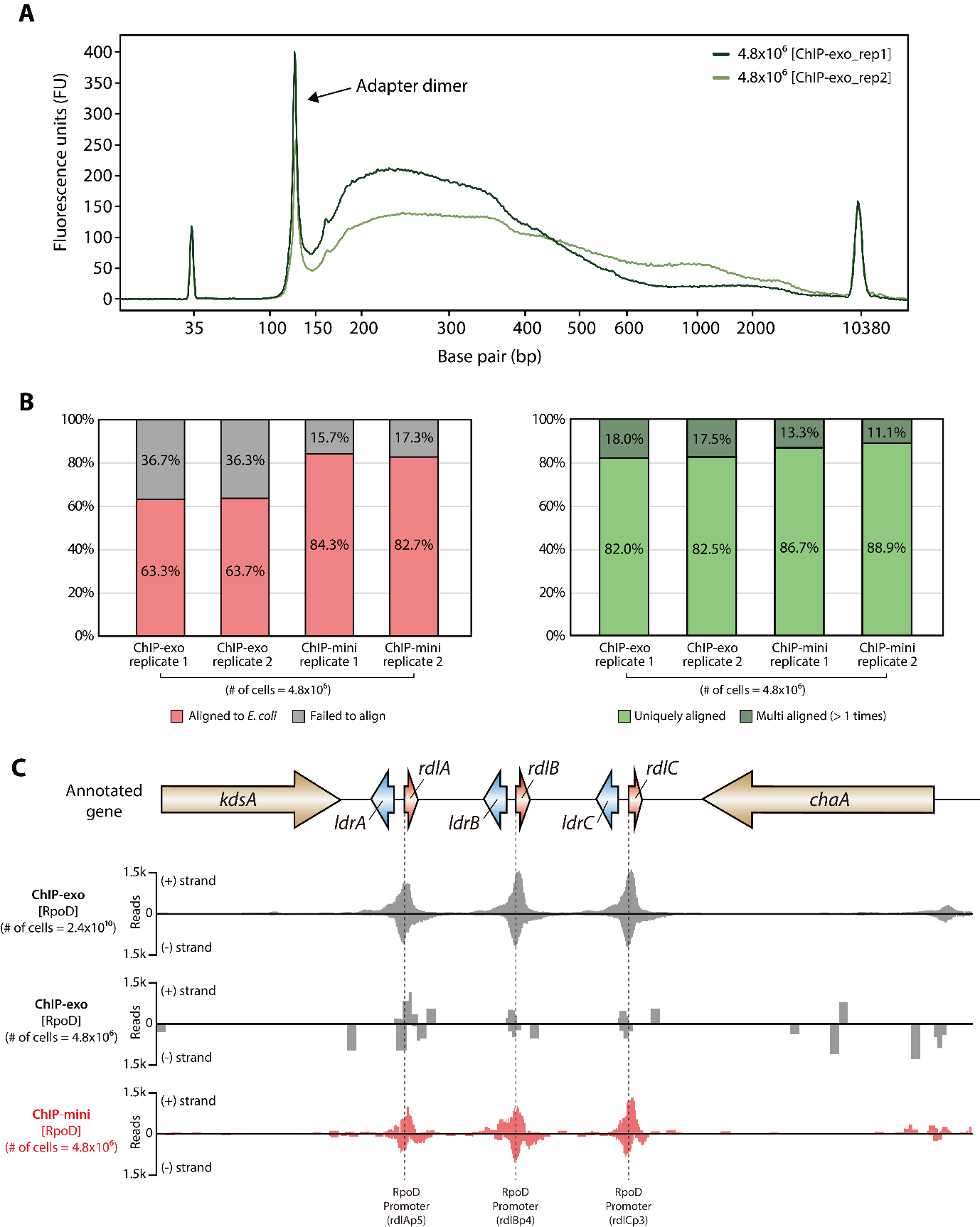


**Figure S3. Comparison of traditional ChIP-exo and ChIP-mini libraries, when using 4.8x10^6^ bacterial cells.** (A) Fragment distribution of traditional ChIP-exo libraries using 4.8x10^6^ *E. coli* cells after PCR amplification. (B) Percentage of aligned and failed-aligned reads on *E. coli* genome of traditional ChIP-exo and ChIP-mini RpoD libraries constructed from 4.8x10^6^ of initial bacterial cells. Uniquely mapping reads were also confirmed to assess library complexity, showing that traditional ChIP-exo libraries exhibit lower complexity than ChIP-mini libraries. (C) Evaluation ChIP-mini against traditional ChIP-exo for delineating RpoD binding profiles. Unlike ChIP-mini, ChIP-exo did not achieve sufficient resolution for identification of RpoD binding profiles at genome-wide level when utilizing a minimal initial quantity of bacterial cells.


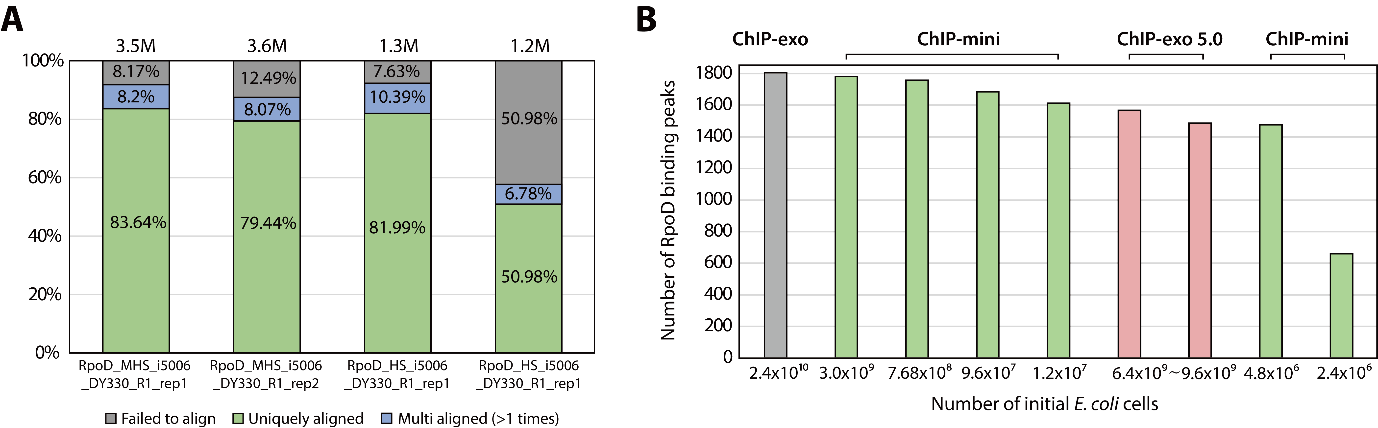


**Figure S4. Comparison of the ChIP-mini and simplified ChIP-5.0 methods.** (A) Percentage of aligned reads on the genome of *E. coli* in RpoD ChIP-exo 5.0 libraries. (B) The number of RpoD binding peaks identified by traditional ChIP-exo, ChIP-mini, and ChIP-exo 5.0.


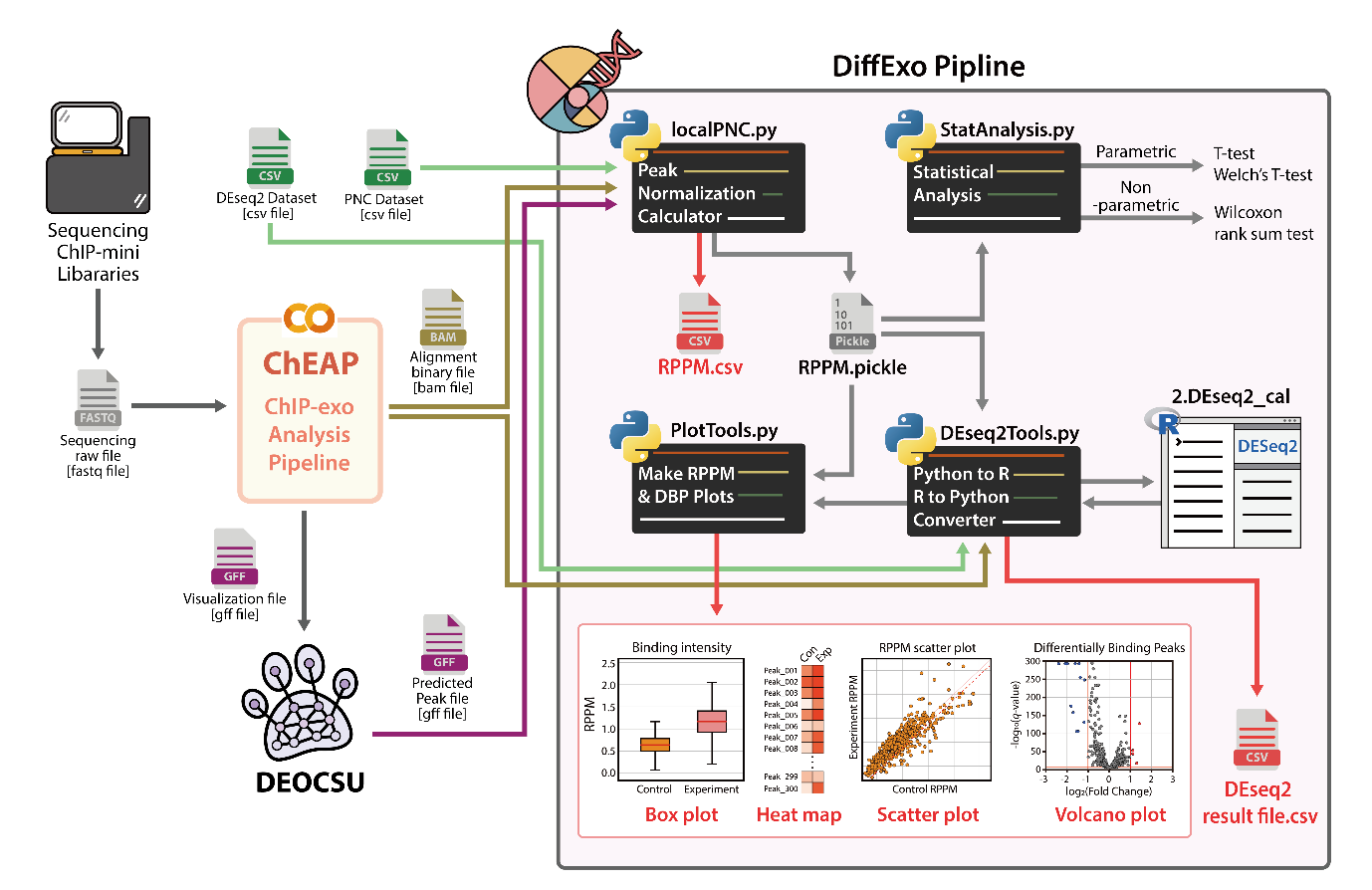


**Figure S5. Schematic of the DiffExo pipeline.** Two dataset files (CSV), including information on the data directories and statistical groups (control or experiment), are formatted before starting the DiffExo pipeline. For preprocessing of ChIP-mini data for the DiffExo pipeline, the sequencing raw files (FASTQ) are converted into alignment files (BAM) and visualization files (GFF) using the cloud-based ChIP-exo analysis pipeline, ChEAP (7). Followed by visualization, a Deep-learning optimized ChIP-exo peak calling suite (DEOCSU) is utilized to predict binding peaks of target DNA-binding protein using GFF files (8). Since all the read count data from each ChIP-mini sequencing library are not normally distributed as they are in an RNA-seq library, a negative binomial distribution-based algorithm was adopted to estimate differentially binding sites (9). In the DiffExo pipeline, 1) localPNC.py: calculate the normalized binding intensity of DNA-binding protein using an RPPM unit and generate a pickle file to convey the normalized data to the next process. 2) StatAnalysis.py: proceed statistical test (parametric or non-parametric test) using the RPPM.pickle file. 3) DEseq2Tools.py: convert overlapping binding sites into reference file for DEseq2, and differential binding sites are calculated in R script (DEseq2_cal). In addition, the raw result file from DEseq2 merges with RPPM and information on binding sites to generate the final Deseq2 result file (CSV) using DEseq2Tools. 4) PlotTools.py: generate four types of plots: box plots, heat maps, scatter plots for visualizing binding intensity, and volcano plots for visualizing DEseq2 results.

**
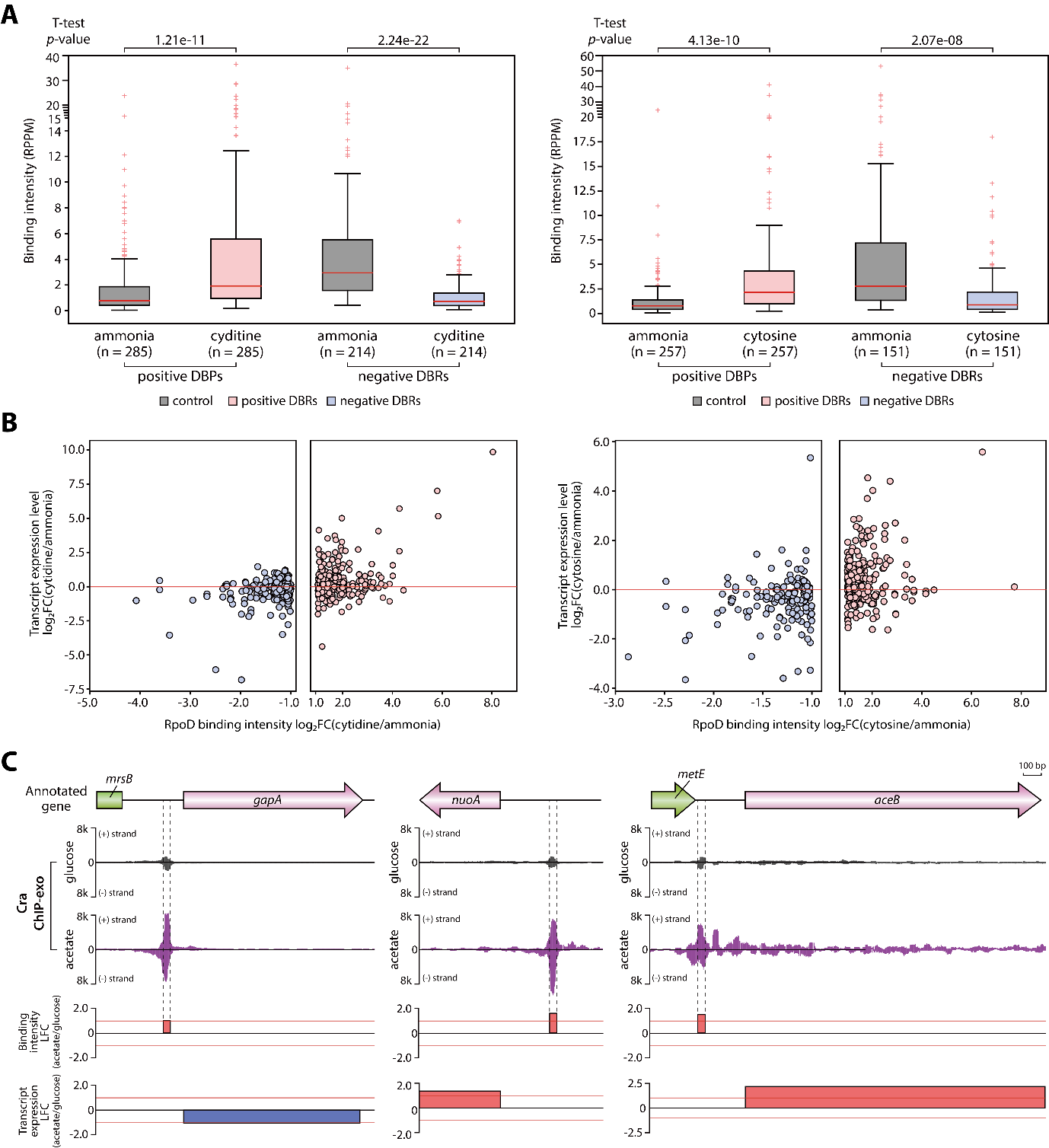
**

**Figure S6. Validation of the DiffExo pipeline using *E. coli* ChIP-exo datasets from Park *et al*. and Kim *et al*.** (A) Boxplot of binding intensity in differentially binding peaks (DBPs) from *E. coli* RpoD ChIP-exo datasets generated under unfavorable nitrogen source conditions (cytidine and cytosine), with ammonia conditions used as a control. (B) The scatter plot illustrates the relationships between binding intensity and transcript expression levels related to RpoD under cytidine or cytosine conditions. The change in binding intensity of RpoD showed similar trends under both conditions. When the binding intensity of RpoD increased, the expression level tended to be up-regulated, whereas when the binding intensity decreased, the expression level tended to be down-regulated. (C) DBPs in Cra binding profiles upstream of their regulon genes. Acetate was used as the sole carbon source, and glucose was used as a control.

**
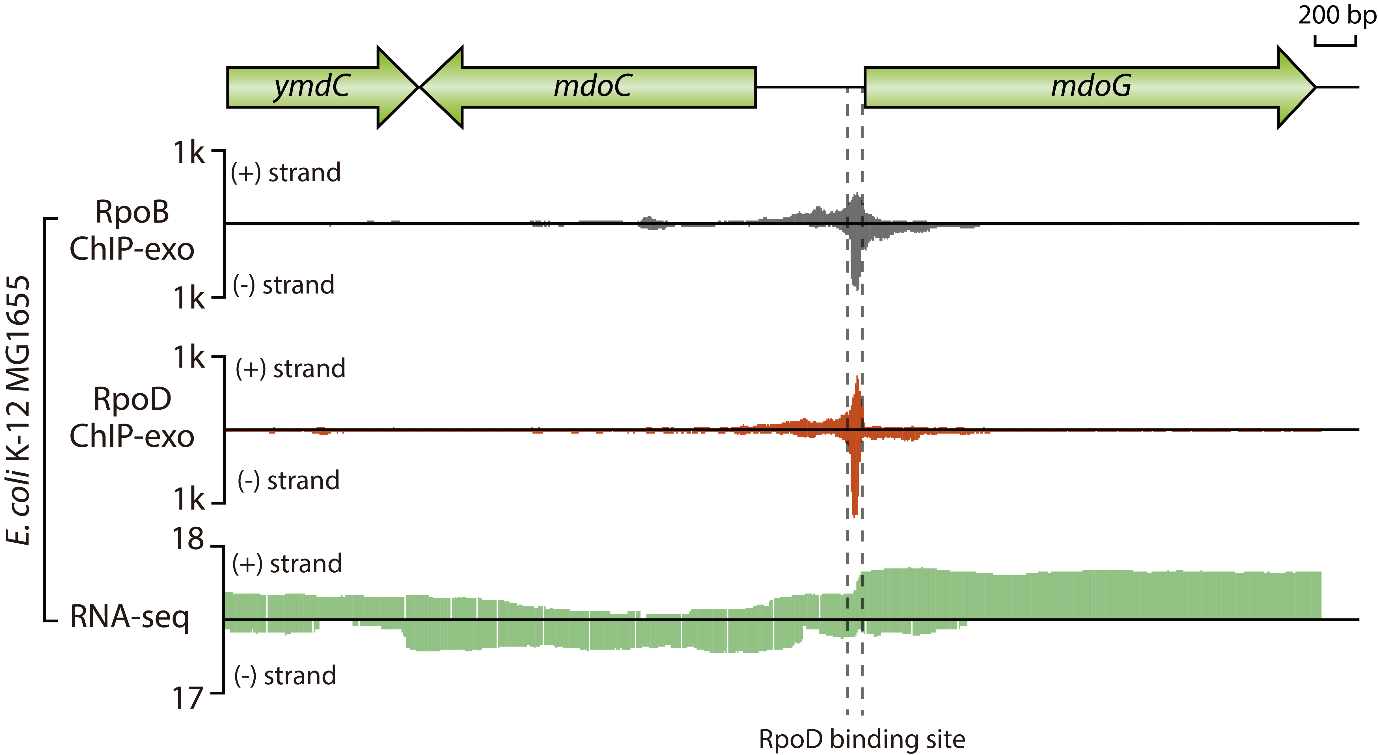
**

**Figure S7. Comparison of RpoD and RpoB binding peaks in ChIP-exo datasets based on Park *et al* (10).**


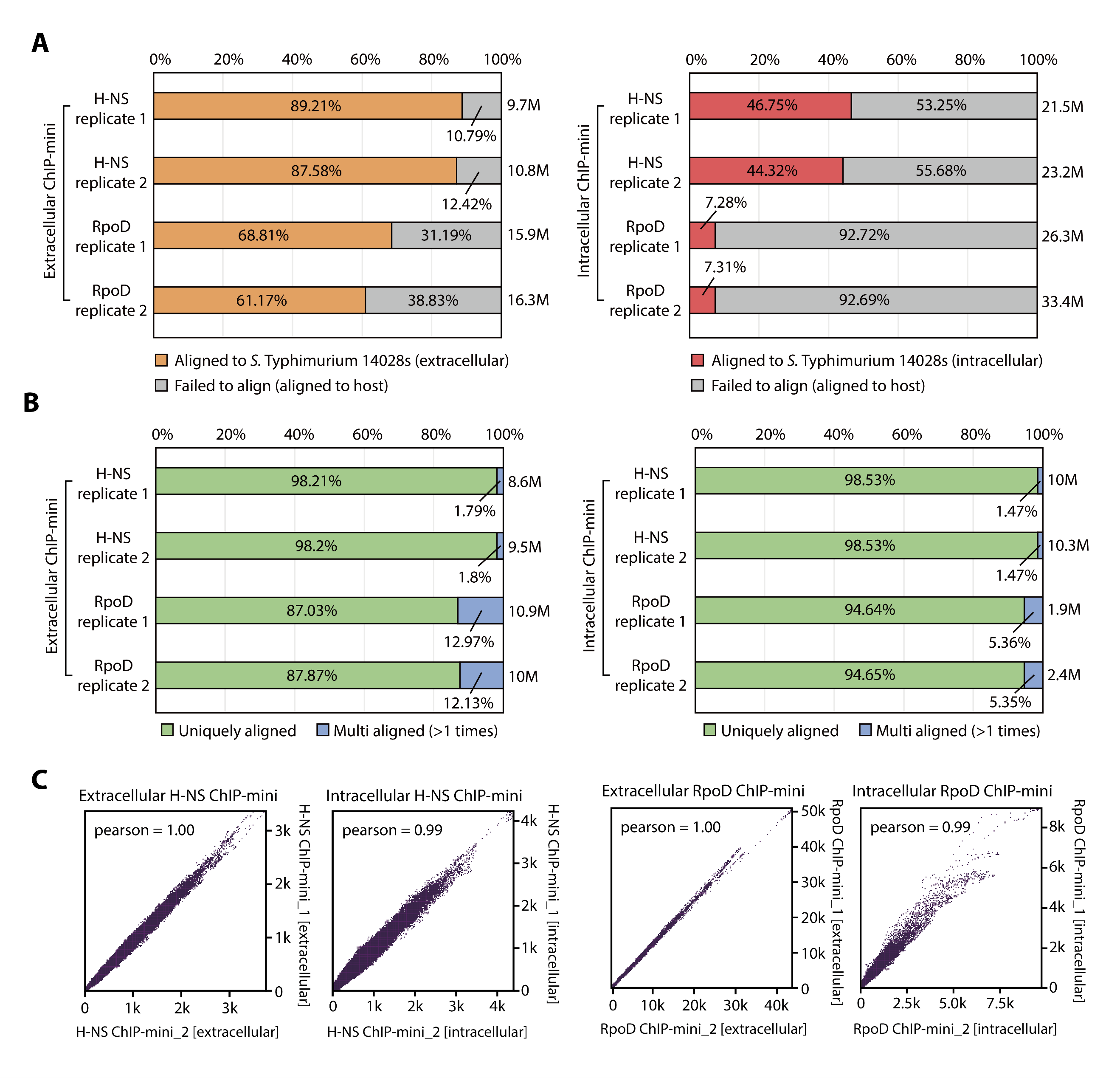
**Figure S8. Detail analysis of sequencing libraries of H-NS and RpoD using extra- and intracellular ChIP-mini methods.** (A) Percentage of aligned and failed-aligned reads on the genome of *S.* Typhimurium in H-NS and RpoD libraries. Failed-aligned reads were confirmed to align the host genome (*Mus musculus*). (B) Percentage of uniquely mapping reads and multi aligned reads on the genome of *S.* Typhimurium in H-NS and RpoD libraries. (C) Correlation between replicates of ChIP-mini libraries for H-NS and RpoD was measured by Pearson coefficient. Read counts of each library were split into 10 bp-bins across the *S.* Typhimurium genome, and each dot in the scatter plots represents one genome region.

**
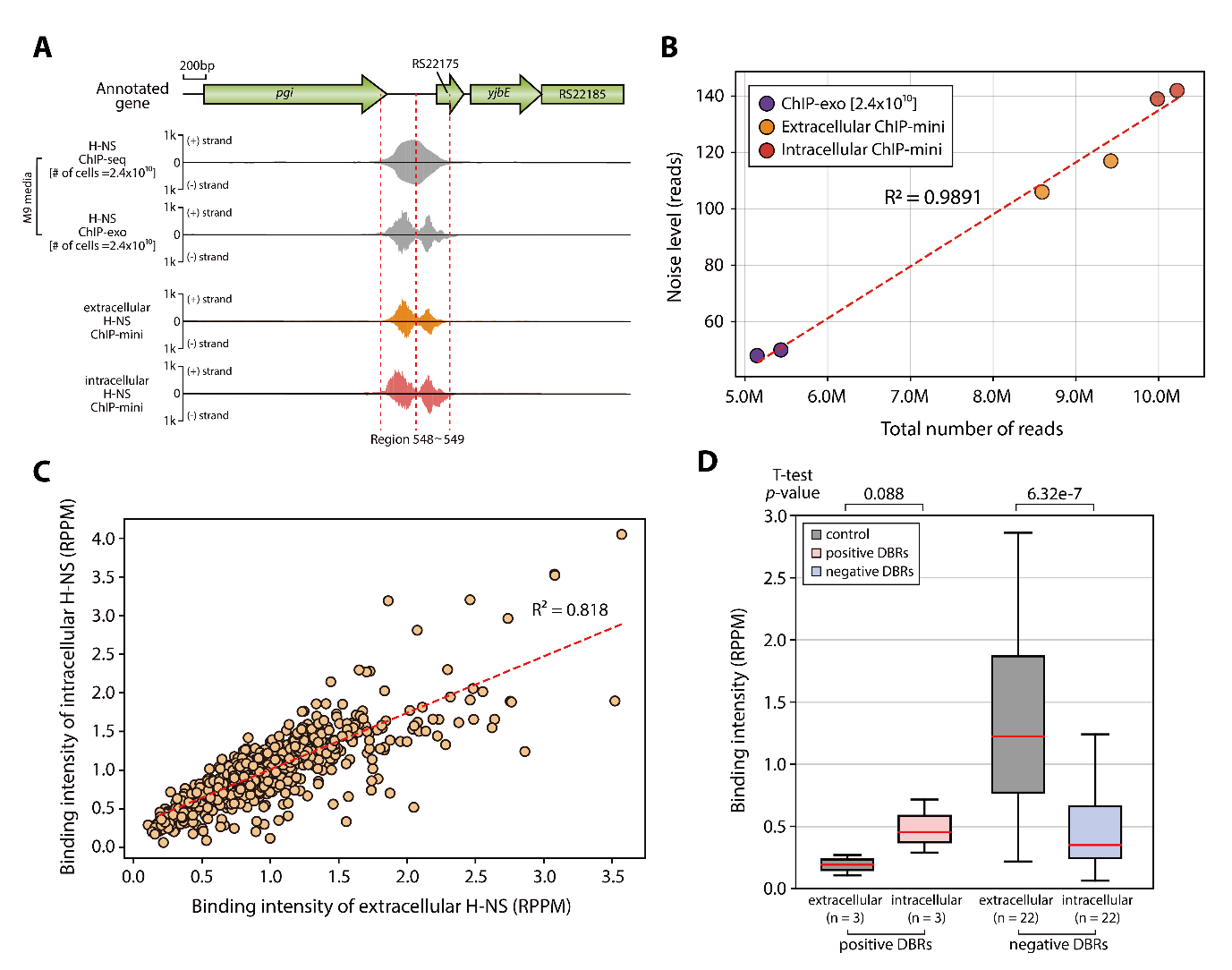
Figure S9. Detail analysis of H-NS binding regions using ChIP-mini applications.** (A) Comparison of H-NS binding regions from ChIP-seq, ChIP-exo, and ChIP-mini. ChIP-mini also provides better resolution compared to ChIP-seq to dissect binding region, even for the H-NS dataset. ChIP-seq and ChIP-exo experiments for H-NS of *S.* Typhimurium were conducted under M9 minimal media. (B) Determination of noise level in H-NS ChIP-exo and ChIP-mini sequencing reads. (C) Pearson correlation coefficient of normalized H-NS binding intensities was calculated from the extra- and intracellular ChIP-mini datasets. (D) A boxplot of binding intensity in differentially binding regions (DBRs) from extra- and intracellular ChIP-mini datasets. The extracellular ChIP-mini dataset was used as a control.

**
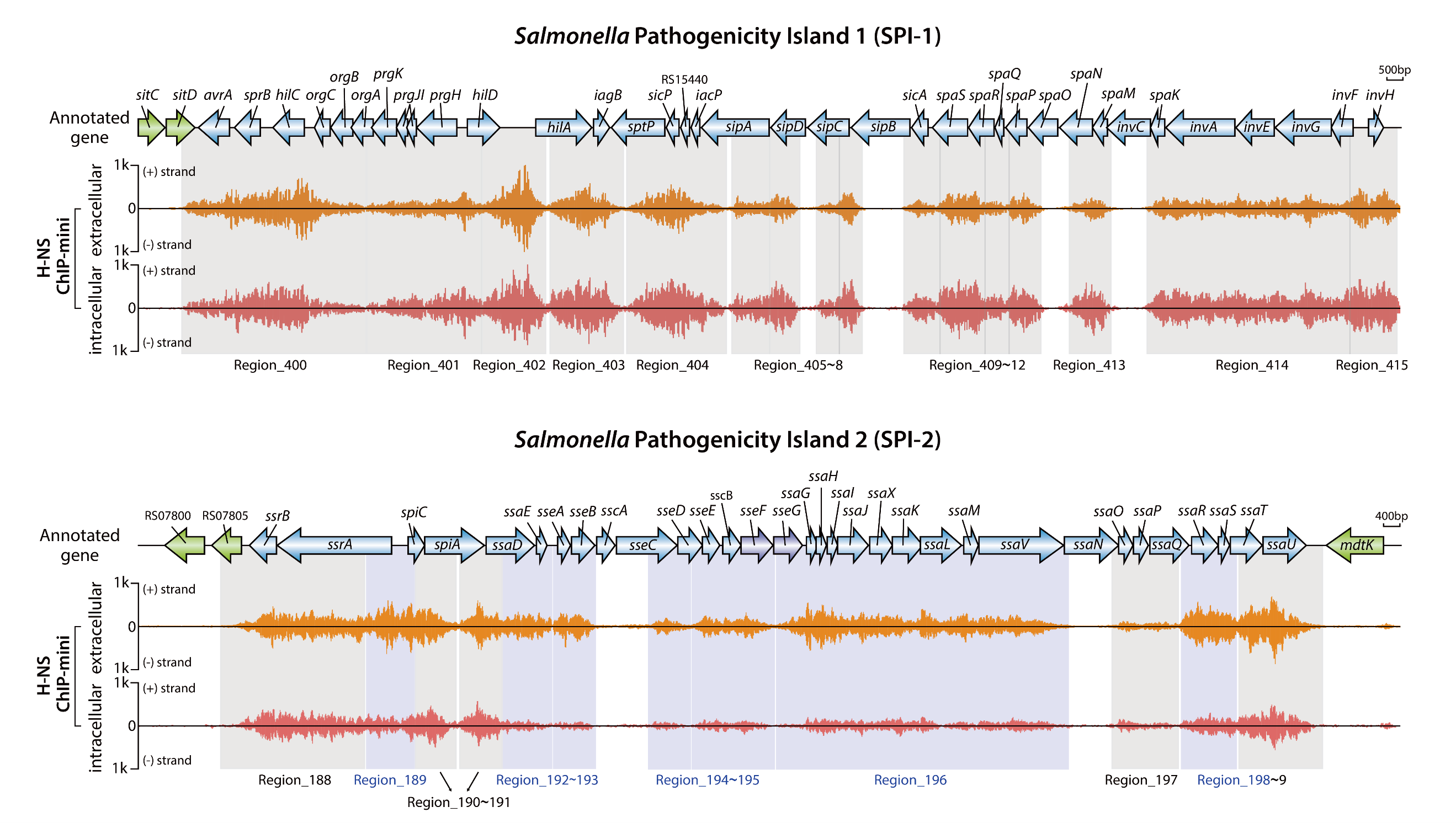
Figure S10. Overview of H-NS binding profiles in SPI-1 and SPI-2 of *S.* Typhimurium within macrophages.** Blue boxes denote negative DBRs, while grey boxes indicate non-DBRs. (+) and (−) strands in ChIP-mini data indicate reads mapped on forward and reverse strands, respectively.


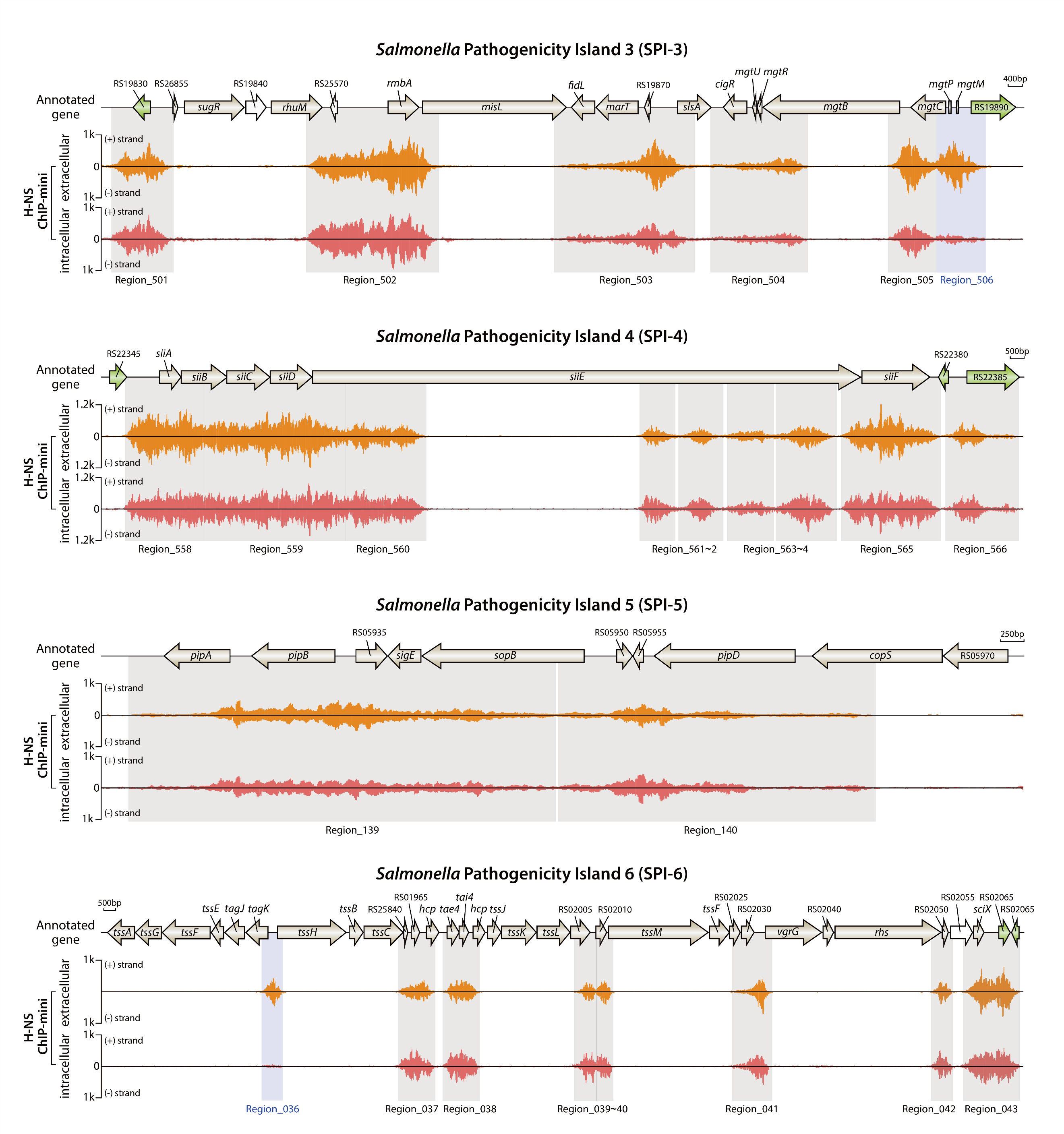
**Figure S11. Overview of H-NS binding profiles in SPI-3~6 of *S.* Typhimurium within macrophages.** Blue boxes denote negative DBRs, while grey boxes indicate non-DBRs. (+) and (−) strands in ChIP-mini data indicate reads mapped on forward and reverse strands, respectively.


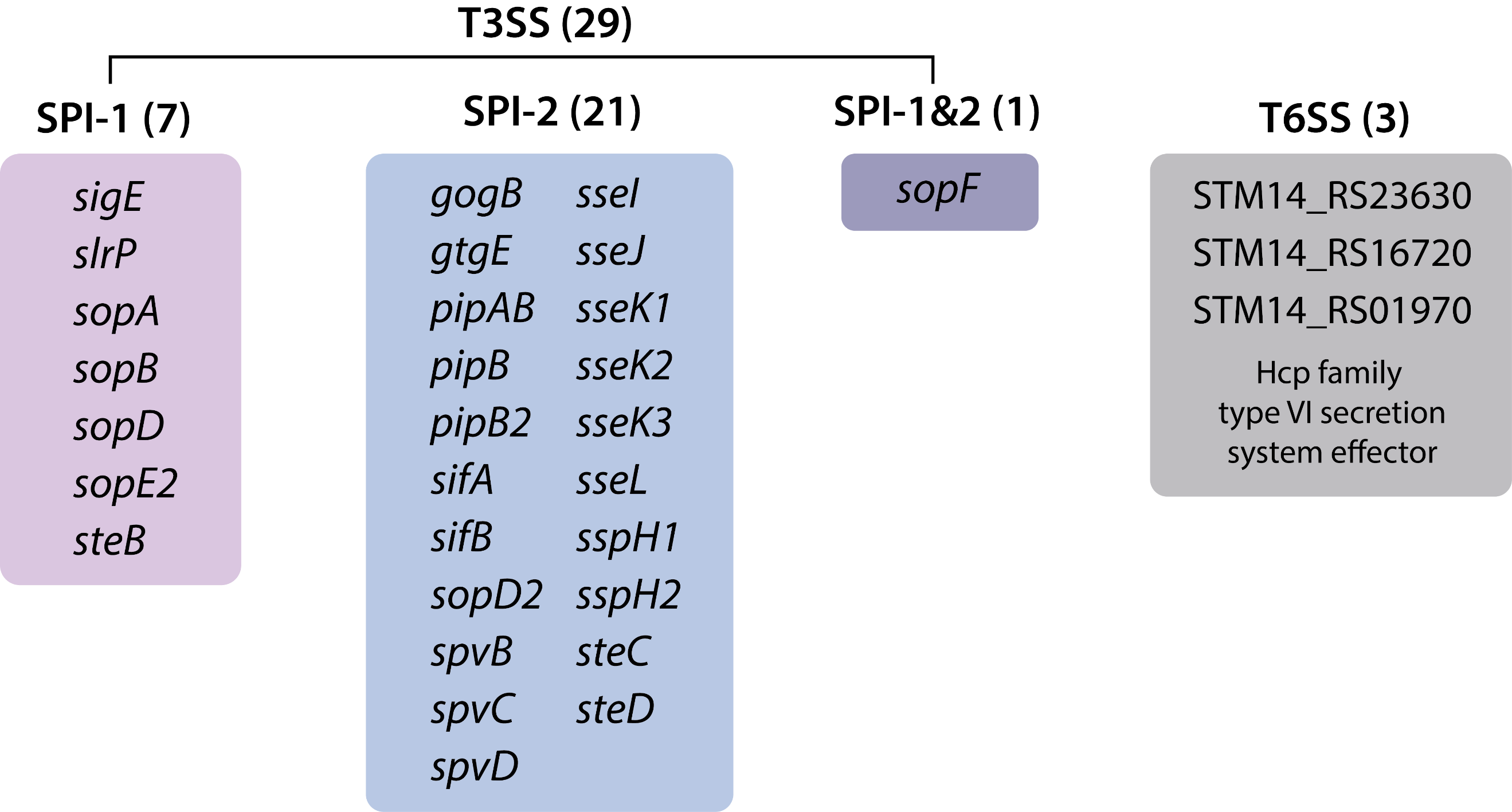


**Figure S12. T3SS and T6SS effector genes associated with H-NS binding regions.** Seven effector genes are related to the SPI-1 T3SS, and 21 effector genes are related to the SPI-2 T3SS. Additionally, *sopF* is associated with both T3SS systems. Three effector genes are associated with the T6SS, encoding the Hcp family effector.

**
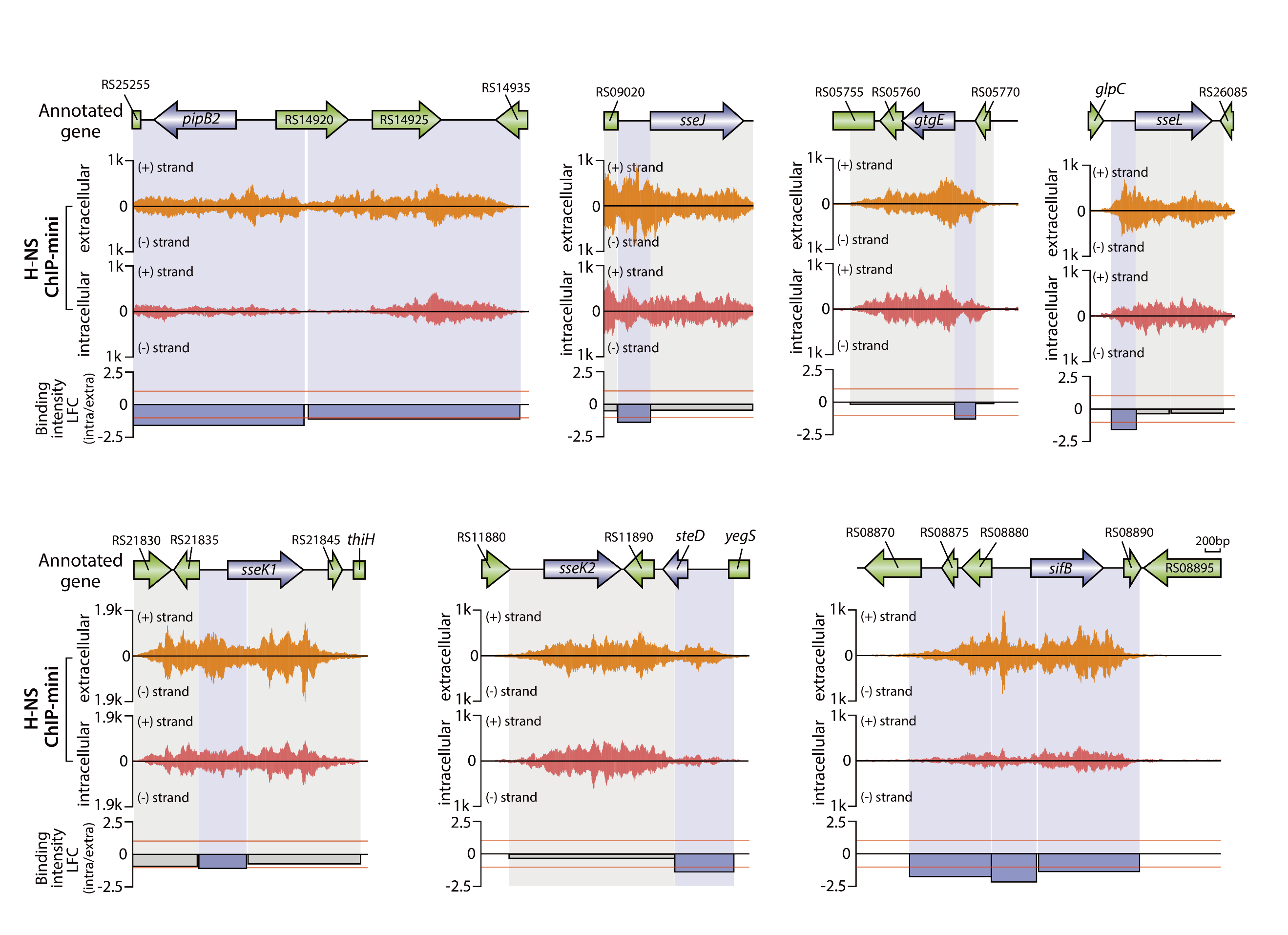
Figure S13. Negative H-NS DBRs on seven SPI-2 effector genes of *S.* Typhimurium.** Under macrophage intracellular conditions, H-NS binding intensity showed a more significant reduction in the intergenic regions of SPI-2 effector genes. The red lines in binding intensity LFC represent the threshold for DBRs, indicated by -1 and 1. Blue boxes denote negative DBRs, while grey boxes indicate non-DBRs. (+) and (−) strands in ChIP-mini data indicate reads mapped on forward and reverse strands, respectively.

**
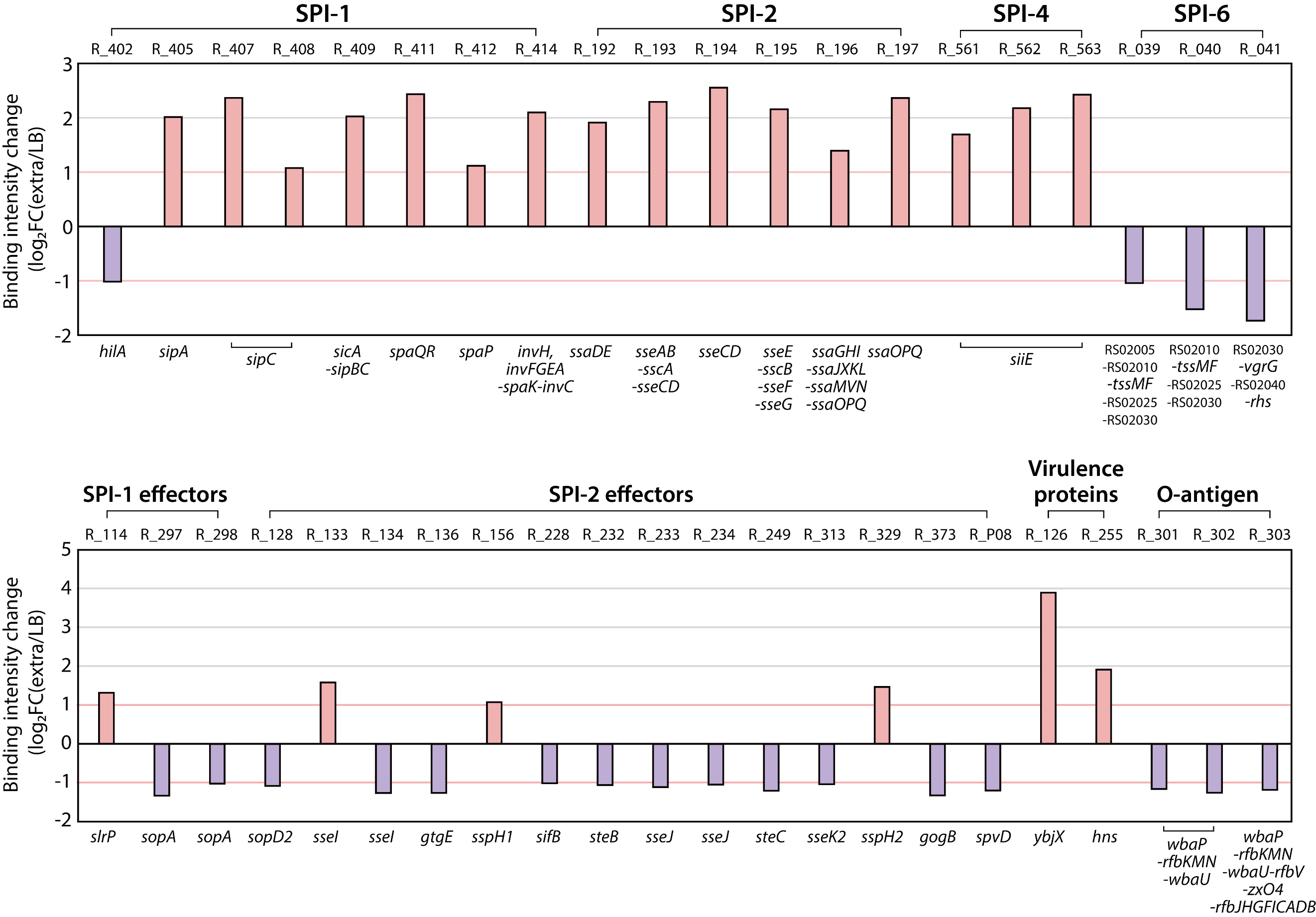
Figure S14. Comparison of H-NS binding intensity changes associated with virulence-related genes between LB stationary and extracellular conditions.**

**
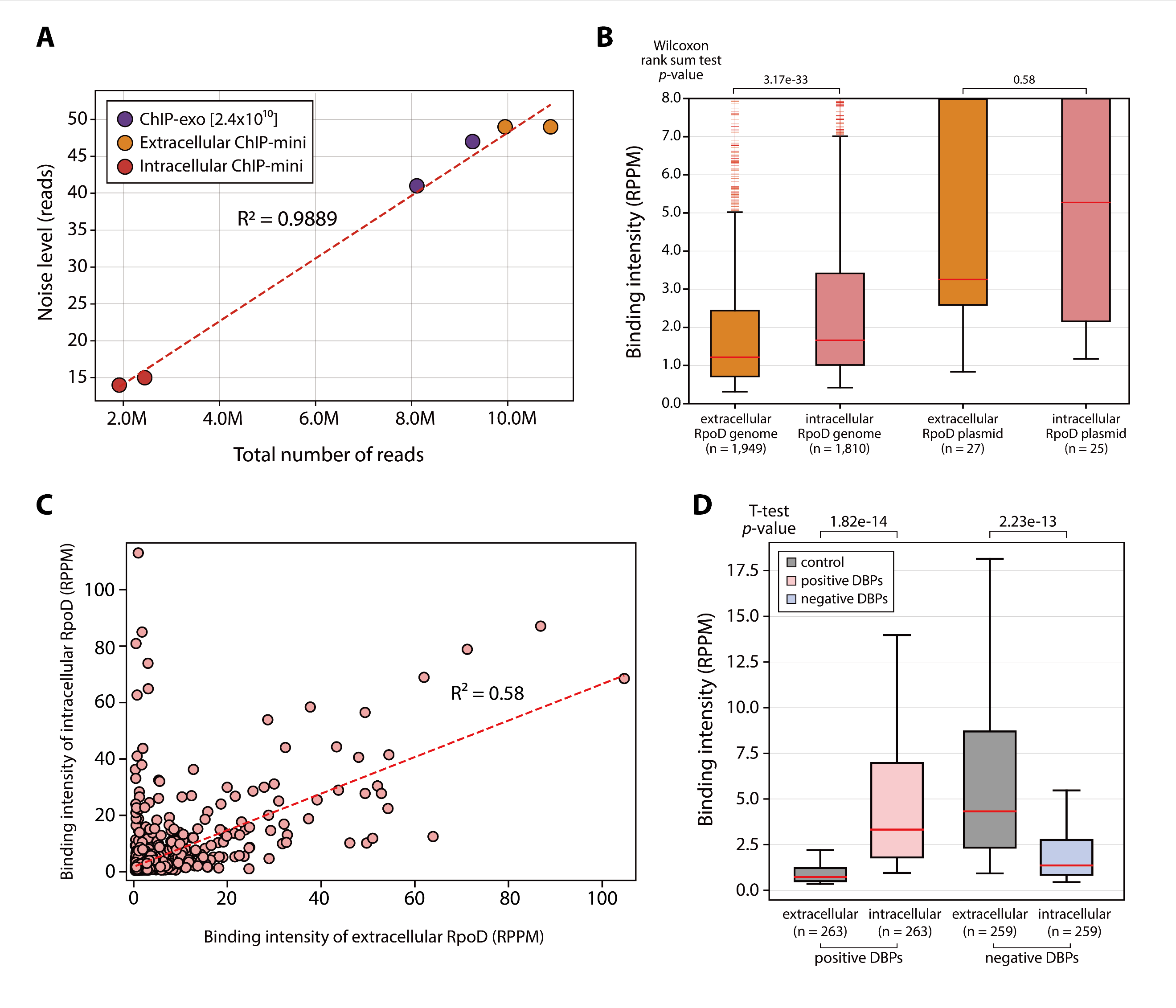
**

**Figure S15. Detail analysis of RpoD binding sites using ChIP-mini applications.** (A) Determination of noise level in RpoD ChIP-exo and ChIP-mini sequencing reads. (B) Normalized binding intensities of total binding sites were calculated using extra- and intracellular RpoD ChIP-mini datasets. (C) Pearson correlation coefficients of normalized RpoD binding intensities were calculated from the overlapping binding sites. (D) Boxplot of binding intensity in differentially binding peaks (DBPs) extra- and intracellular ChIP-mini datasets. The extracellular ChIP-mini dataset was used as a control.

**
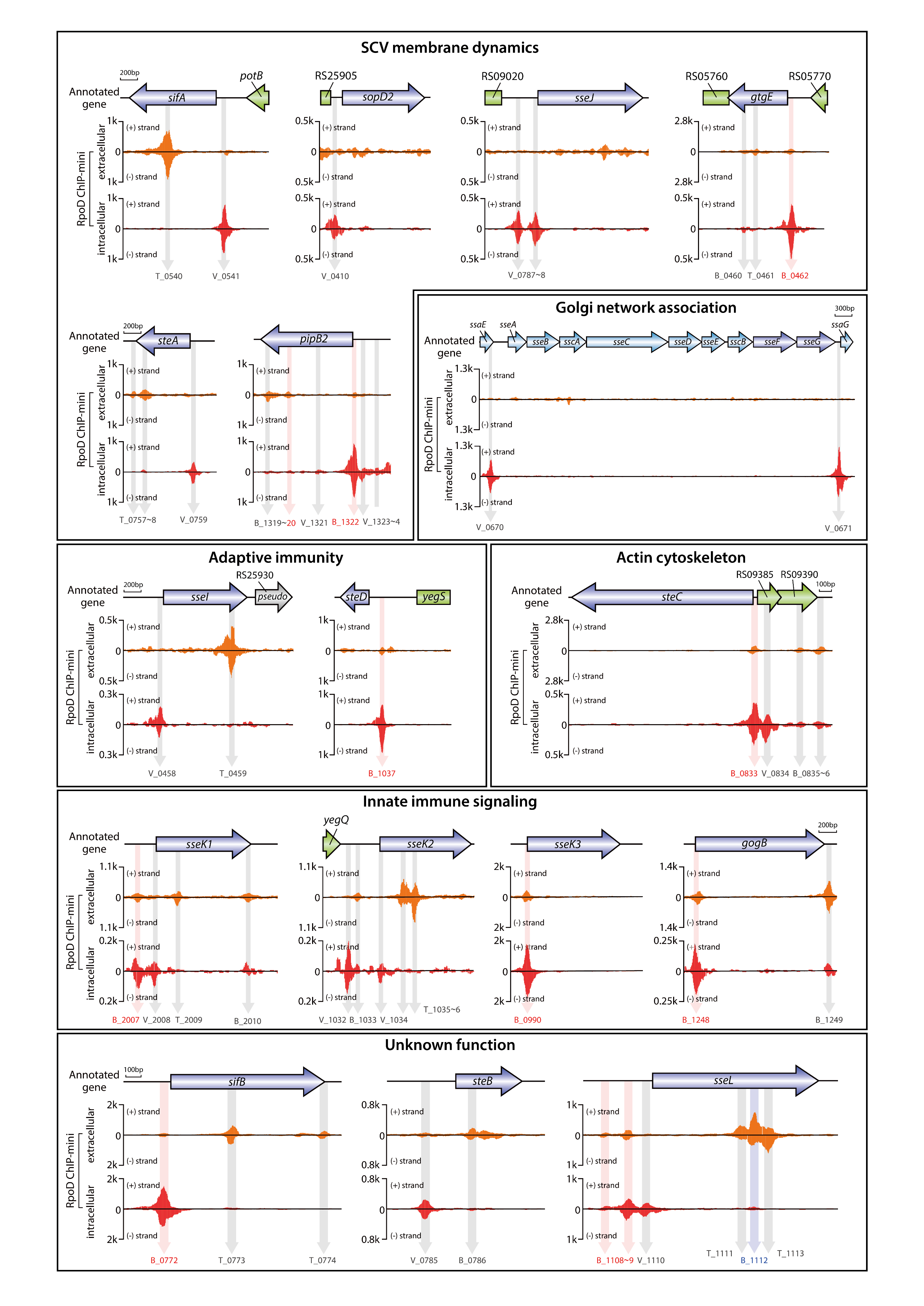
**

**Figure S16. Overview of *S.* Typhimurium SPI-2 effector genes with RpoD binding profiles under macrophage intracellular conditions.** SPI-2 effector genes were classified based on Jennings *et al* (11).


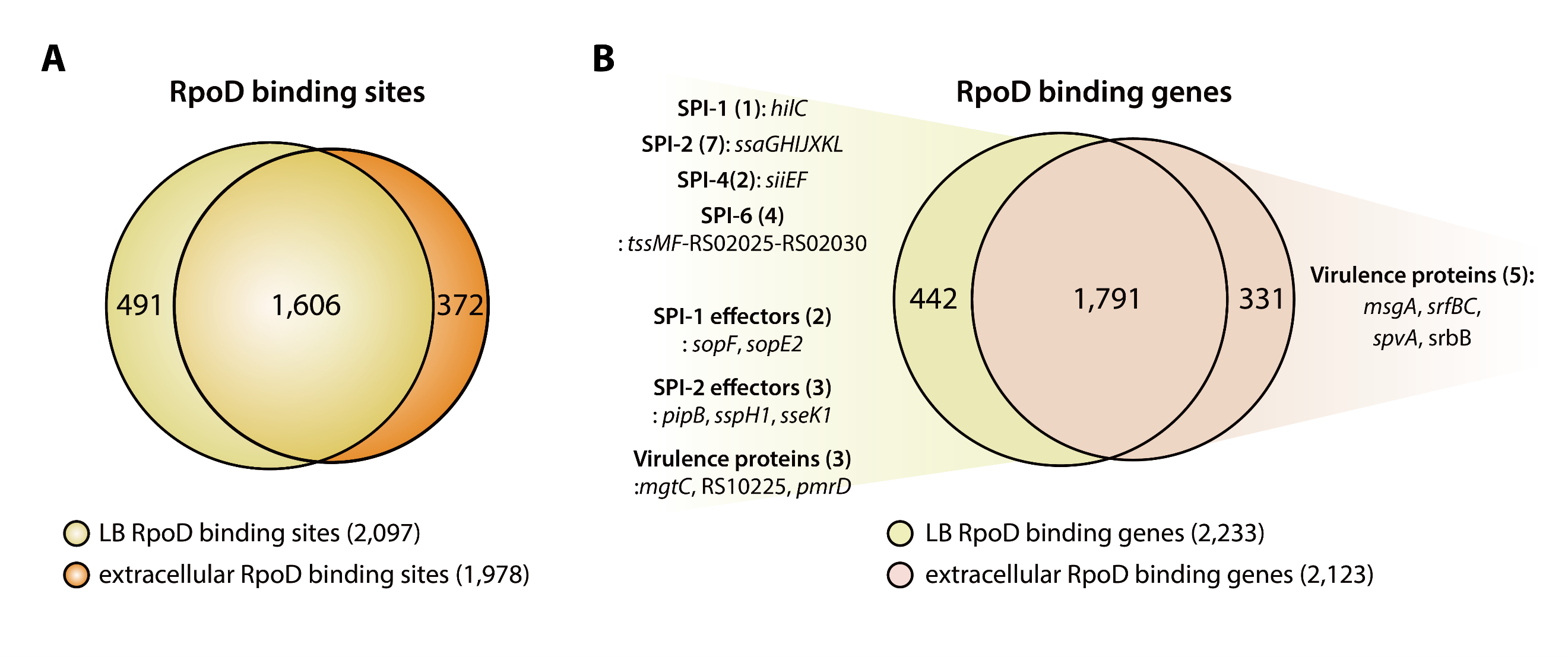


**Figure S17. Comparison of RpoD binding sites between LB stationary and extracellular conditions.** (A) Venn diagram showing the overlap of RpoD binding sites under LB stationary and extracellular conditions. (B) Venn diagram illustrating RpoD target genes shared between both conditions, as well as virulence-related genes exclusively found in each condition.

**
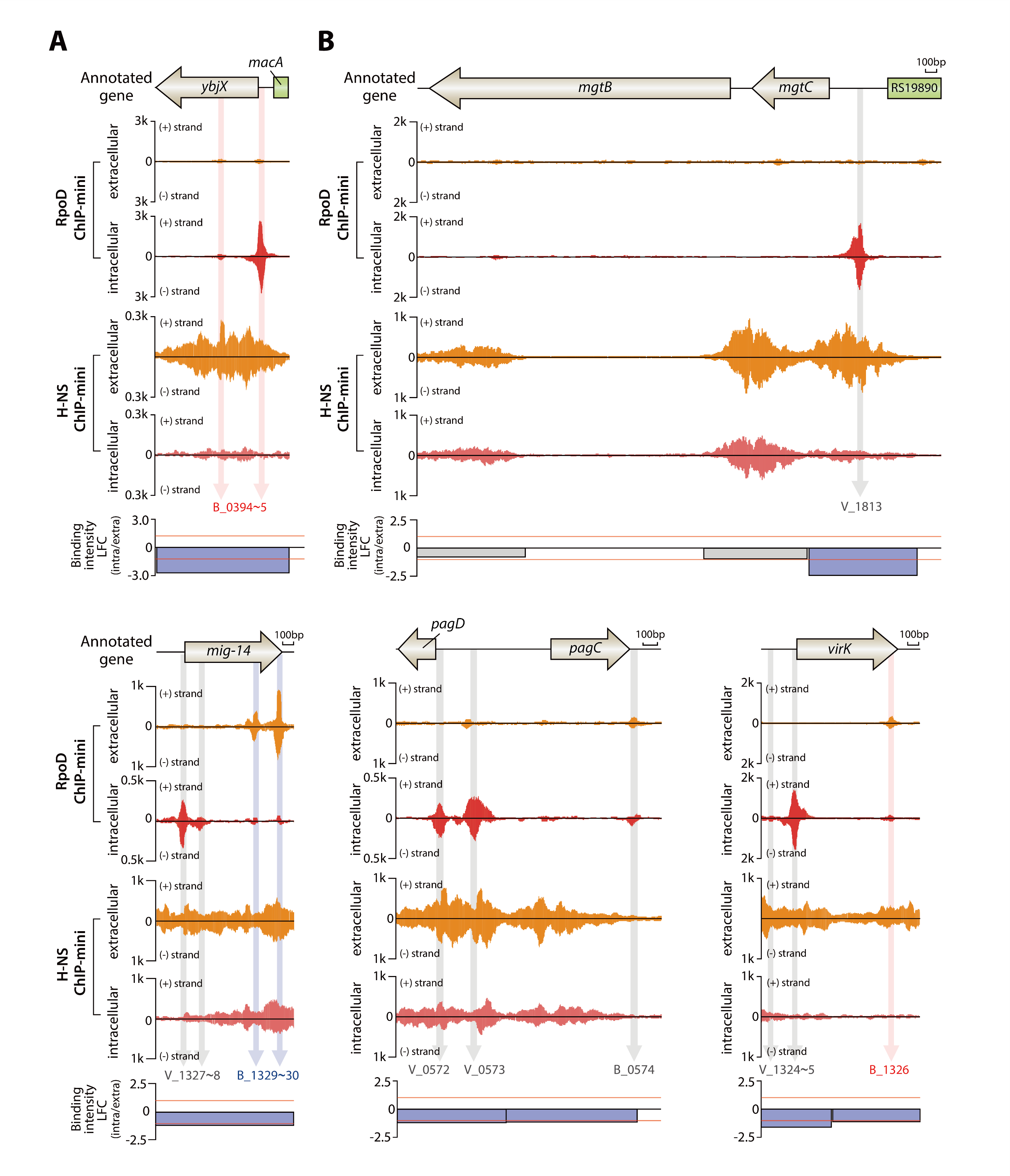
Figure S18. Dynamic changes in RpoD binding sites on other virulence genes under the influence of H-NS negative DBRs.** (A) *ybjX* was identified as overlapping virulence genes between RpoD positive DBP genes and H-NS negative DBR genes. (B) Five overlapping virulence genes between intracellular unique RpoD genes and H-NS negative DBR genes.

**
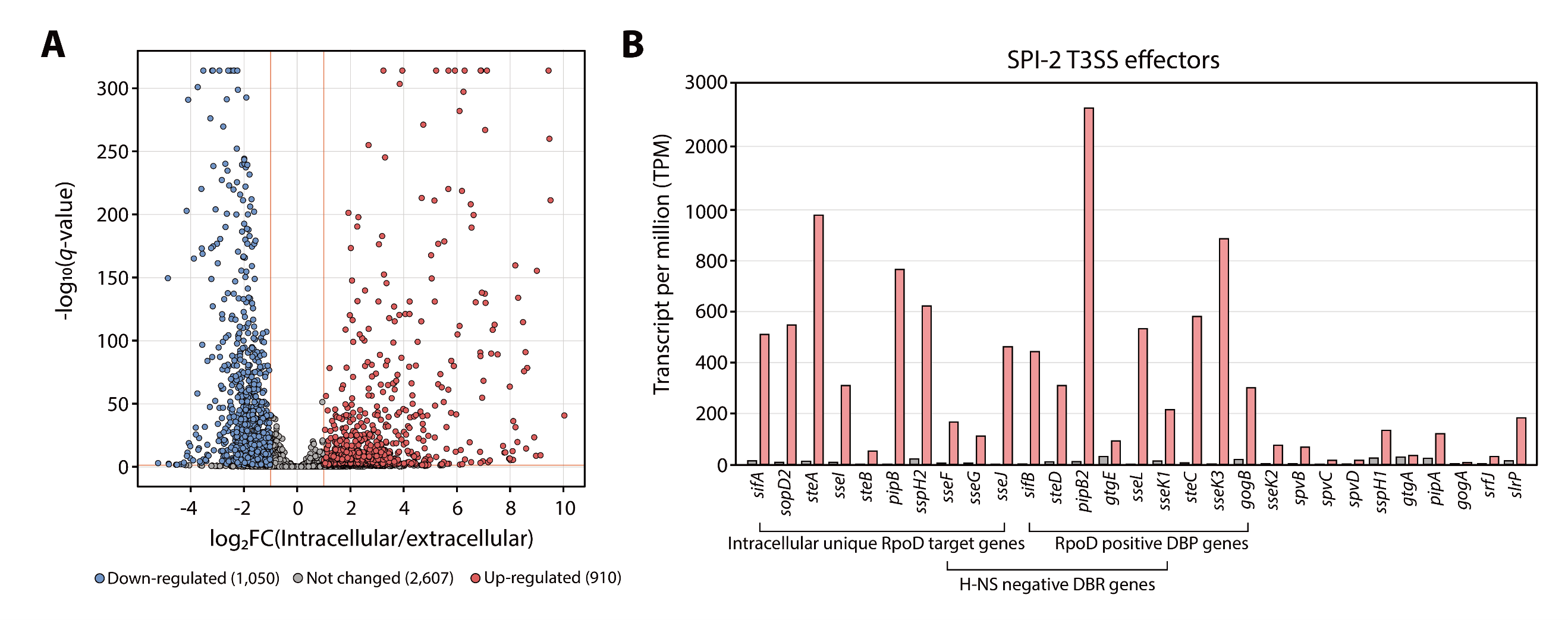
Figure S19. Comparison of transcript expression levels between macrophage extracellular and intracellular conditions.** (A) Volcano plot displaying differentially expressed genes (DEGs) as response to environmental changes from macrophage extracellular and intracellular conditions (absolute value of log_2_ fold change ≥ 1, and false discovery rate < 0.05). (B) The mRNA expression level of SPI-2 effector genes under both conditions.

**
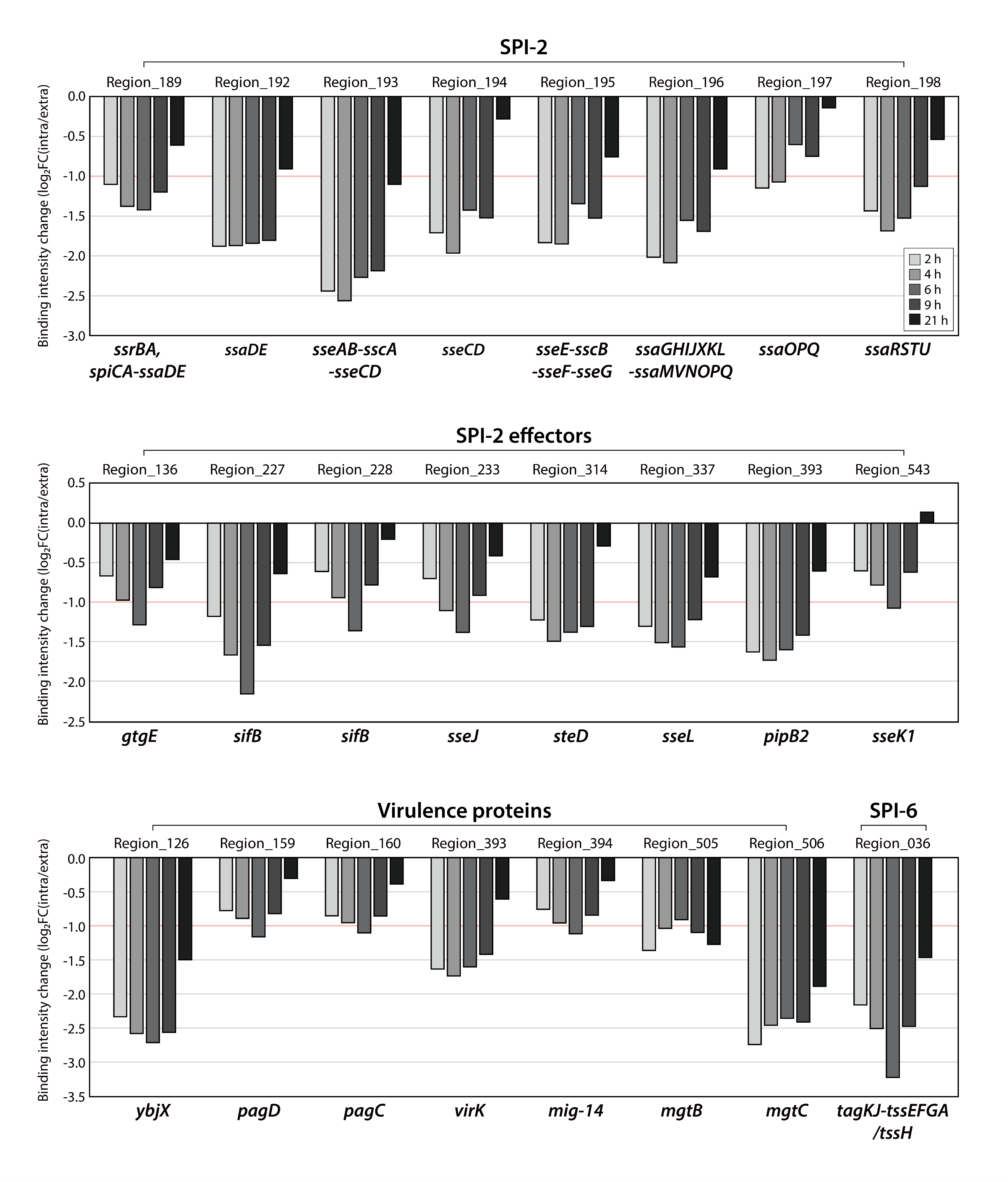
**

**Figure S20. Comparison of H-NS binding intensity changes associated with virulence-related genes across different post-infection time points.**


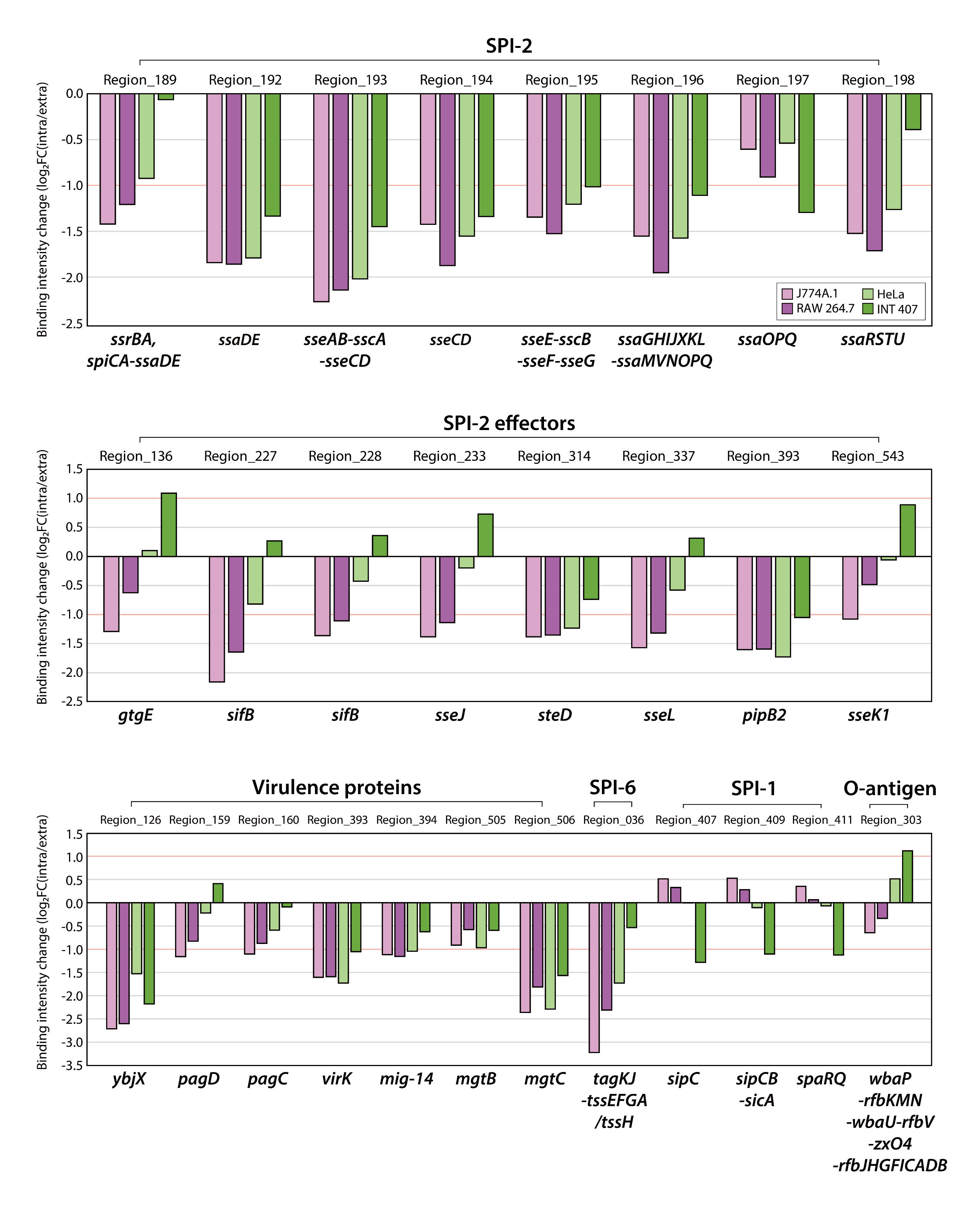


**Figure S21. Comparison of H-NS binding intensity changes associated with virulence-related genes in different types of host cells.**

**
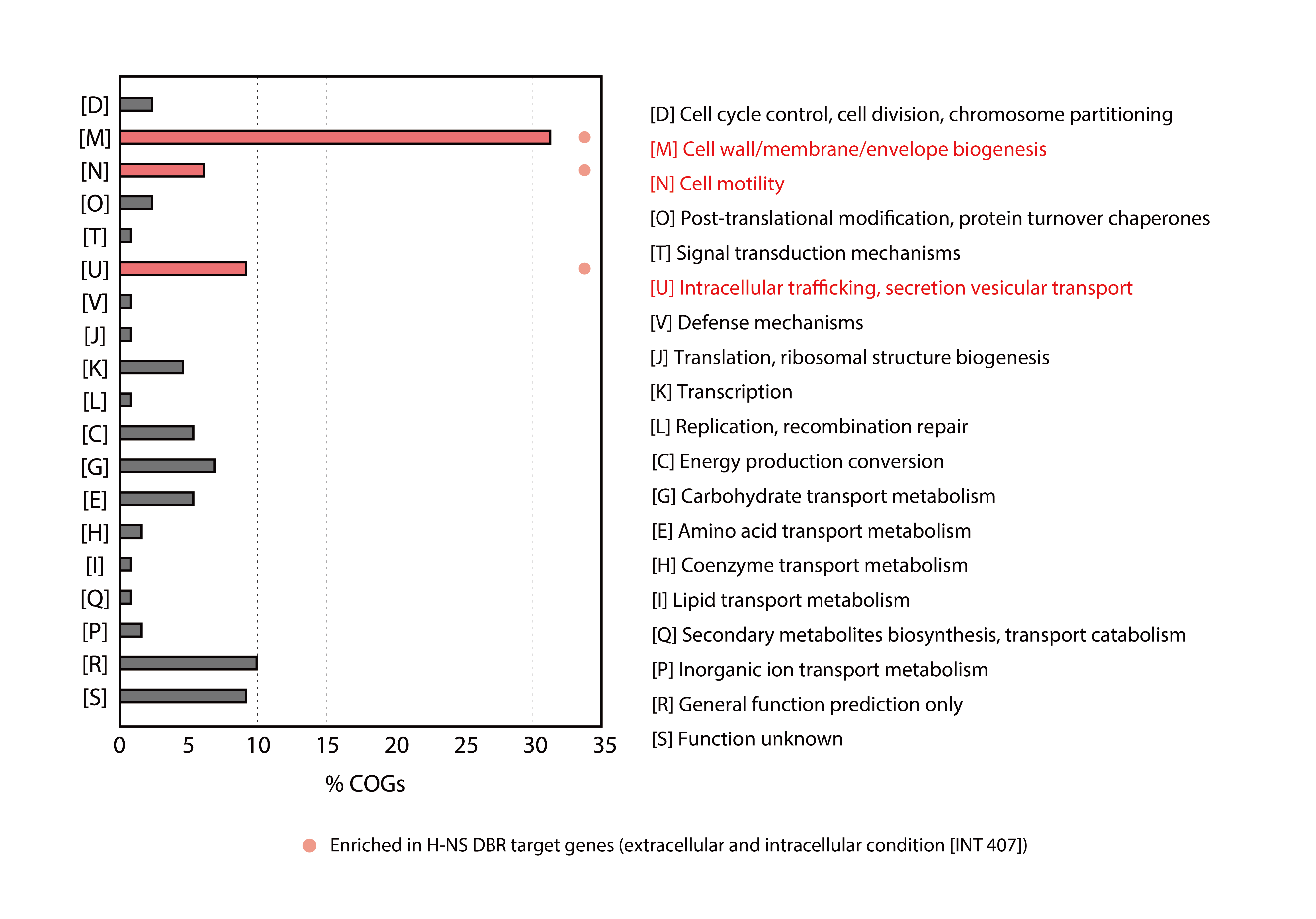
**

**Figure S22.** **Clusters of orthologous groups (COG) analysis on H-NS DBRs in INT 407 cells.** H-NS DBRs in INT 407 cells have functions enriched in “Cell wall/membrane/envelope biogenesis”, “Cell motility”, and “Intracellular trafficking/secretion vesicular transport” categories (Hypergeometric test *p*-value < 0.05).

**
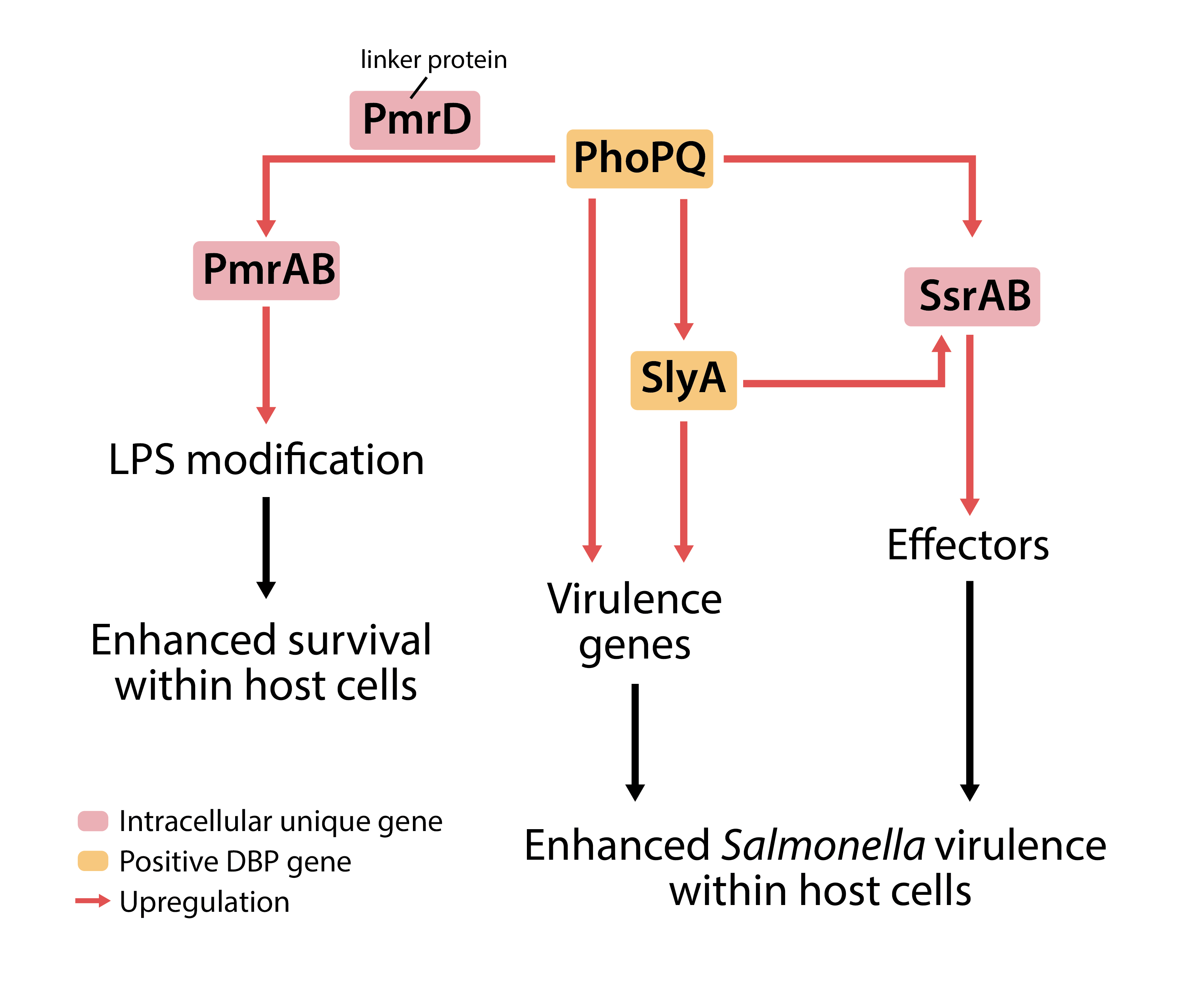
**

**Figure S23. Regulatory cascades activating the expression of SPI-2 effectors and LPS modification genes.** Pink boxes denote intracellular unique RpoD binding gene, and orange box indicates RpoD positive DBP gene.

**
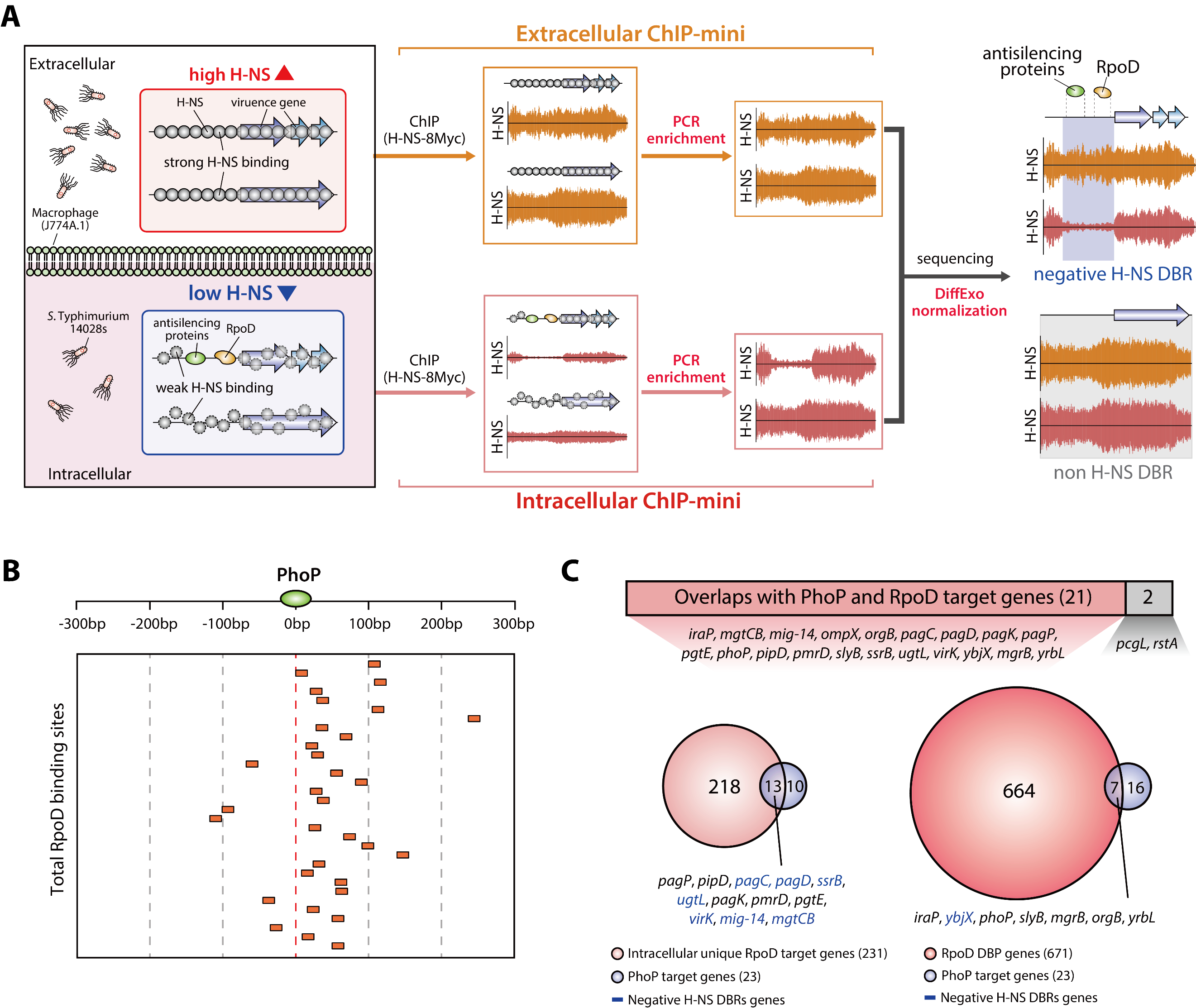
**

**Figure S24. Comparison of antisilencing protein binding sites with RpoD and H-NS binding sites.** (A) Overview of how the depletion of H-NS impacts ChIP-mini datasets. Depletion of H-NS due to proteolysis leads to an overall reduction in total IP-DNA, which is compensated for PCR enrichment and normalization in the DiffExo pipeline. As a result, this overall reduction is categorized as non-DBRs. In contrast, localized depletion of H-NS caused by antisilencing proteins and RpoD results in a specific reduction in total IP-DNA, which cannot be compensated by PCR enrichment and normalization in the DiffExo pipeline. Consequently, this specific reduction is categorized as negative DBRs. (B) The majority of PhoP binding sites were found in upstream of RpoD. C) Analysis of the overlapping target genes between PhoP and RpoD under macropahge intracellualr conditions. 21 out of 23 PhoP target genes were identified among intracellular unique RpoD target genes and RpoD DBP genes. The gene *ompX* was found in non-DBP genes under both conditions. Genes denoted in blue font are associated with H-NS negative DBRs.

**
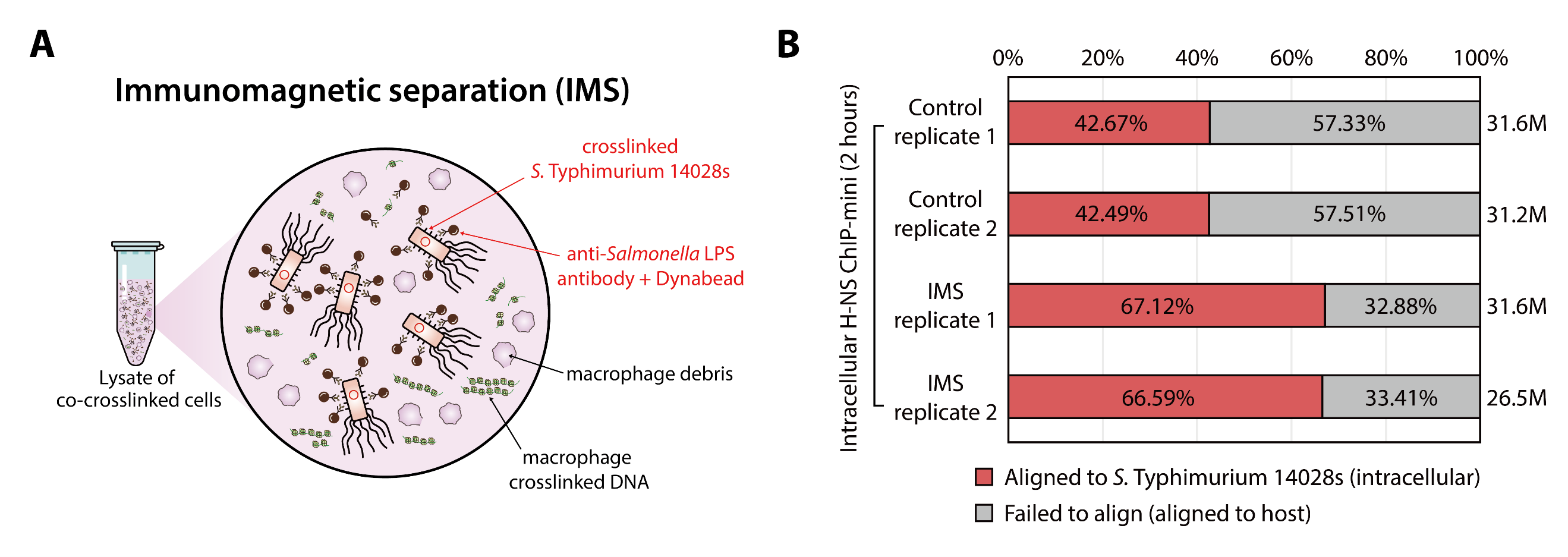
Figure S25. Overview of immunomagnetic separation (IMS) to reduce host DNA contamination in intracellular ChIP-mini libraries.** (A) Method for isolating *Salmonella* from the lysate of co-crosslinked macrophages using IMS. An antibody specific to *Salmonella* LPS was employed to prepare the antibody-magnetic bead complex. The antibody-magnetic beads capture *Salmonella* in the lysate of co-crosslinked macrophages for subsequent intracellular ChIP-mini. (B) Percentage of aligned and non-aligned reads on the *S.* Typhimurium genome in intracellular H-NS libraries (J774A.1 cells at 2 hours post-infection). Failed-aligned reads were confirmed to align the host genome (*Mus musculus*).


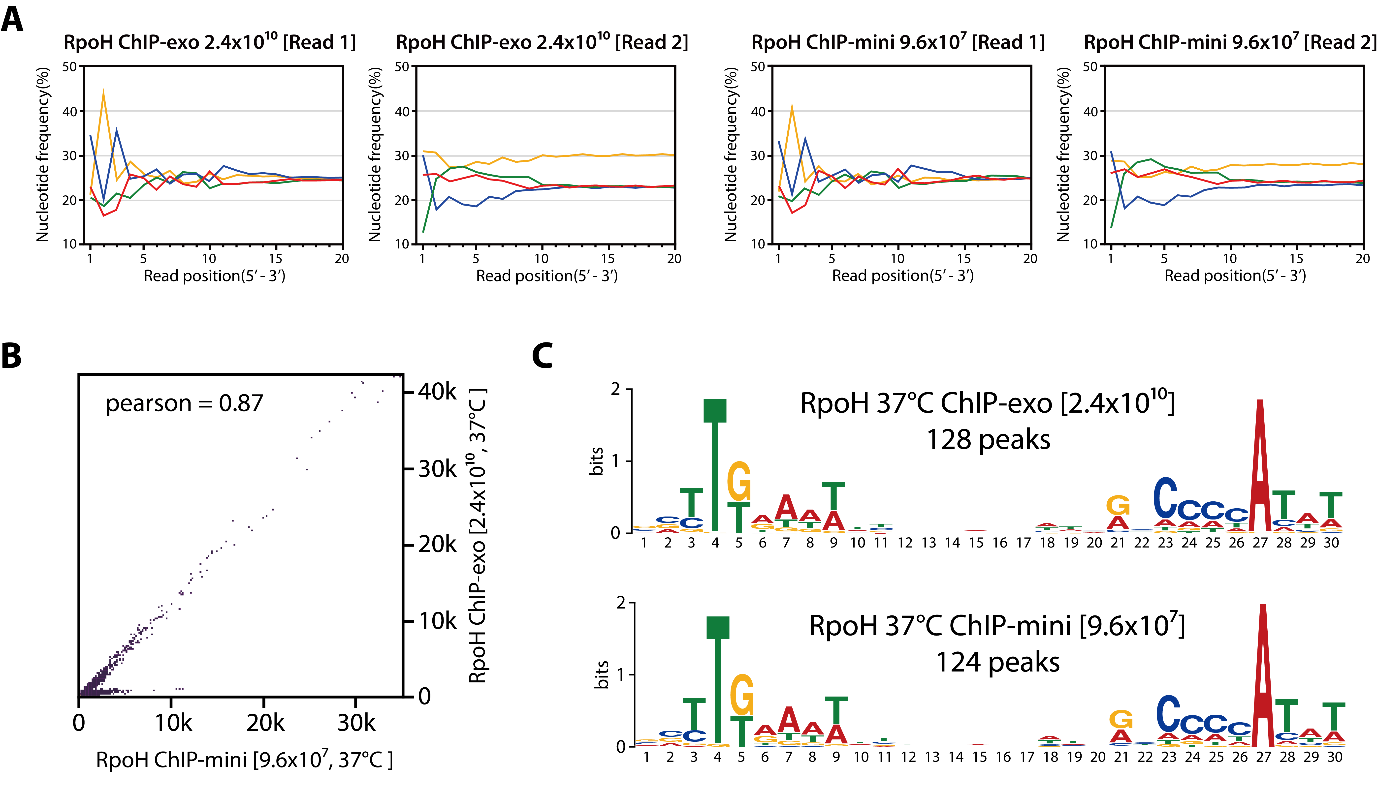


**Figure S26. Comparison of *E. coli* RpoH traditional ChIP-exo and ChIP-mini datasets.** (A) Nucleotide frequency at the 5’ end of the paired-end sequencing reads for traditional ChIP-exo and ChIP-mini libraries for RpoH. (B) Scatter plot depicting the comparative analysis of the sequence alignment BAM files from traditional ChIP-exo and ChIP-mini results. The read counts from each library were segmented into 10-bp bins across the *E. coli* K-12 MG1655 genome, with each dot in the plot symbolizing a specific genomic region. Abbreviation: Pearson’s correlation coefficient: pearson (C) Motif analysis of RpoH binding sites was performed on traditional ChIP-exo and ChIP-mini datasets, resulting in identical sequence motifs

**Supplementary Methods**

**▶ ChIP-mini (ChIP-exo minimization for bacterial cells)**

- **Oligonucleotides and adapters**

| **Oligonucleotides / adapters** | | **Sequences** |
| --- | --- | --- |
| First adapter sense | 5′ **[Phosphate]**-GTGACTGGAGTTCAGACGTGTGCTCTTCC  GATCT 3′ | |
| First adapter anti-sense | 5′ GATCGGAAGAGCACACGTCTGAACTCCAGTCACTT 3′ | |
| Second adapter sense | 5′ AATGATACGGCGACCACCGAGATCTACACTCTTTCC  CTACACGACGCTCTTCCGATCT 3′ | |
| Second Adapter antisense | 5′ GATCGGAAGAGCGTCGTGTAGGGAAAGAGTGTAGA  TCTCGGTGGTCGCCGTATCATTCC 3′ | |
| Second strand synthesis primer | 5′ GTGACTGGAGTTCAGACGTGTGCT 3′ | |
| Illumina non-indexed primer | 5′ AATGATACGGCGACCACCGAGAT 3′ | |
| Illumina indexed primer | 5′ CAAGCAGAAGACGGCATACGAGAT[XXXXXX]GTGA  CTGGAGTTCAGACGTGTGCTCTTCCGATCT 3′ | |

**1. Crosslinking Preparation**

- Prepare crosslinking mix as below:

| **Components** | **3.0x10^9^** | **7.68x10^8^** | **≤ 9.6x10^7^** |
| --- | --- | --- | --- |
| Formaldehyde (37% w/w) | 175 µl | 44 µl | 6 µl |
| Washing TBS (pH 7.4) | 6.5 ml | 1.6 ml | 200 µl |

*Incubate for 25 min at RT, spin down and wash 3 time with ice-cold TBS.*

For samples with a culture volume of less than 200 μl (# of bacterial cells < 9.6x10^7^), crosslinking cells were prepared through serial dilution, starting with a 200 μl sample.

**2. Fragmentation and Preparation of Antibody-TF complex**

- Prepare lysis buffer as below:

| **Components** | **Volume** |
| --- | --- |
| 1 M Tris-HCl (pH 7.5) | 5 mL |
| 5 M NaCl | 10 mL |
| 0.5 M EDTA | 1 mL |
| Nuclease-free TDW | 484 mL |
| **Total Volume** | **500** **mL** |

*Note: The final concentration of lysis buffer is 10 mM Tris-HCl (pH 7.5), 100 mM NaCl and 1 mM EDTA.*

- Prepare protease inhibitor cocktail (PIC) mix as blow:

| **Components** | **Volume** |
| --- | --- |
| 1 M Tris-HCl (pH 7.5) | 5 mg |
| DMSO | 25 µl |
| Nuclease-free TDW | 75 µl |
| **Total Volume** | **100** µl |

- Prepare IP buffer as below:

| **Components** | **Volume** |
| --- | --- |
| 1 M Tris-HCl (pH 7.5) | 50 mL |
| 5 M NaCl | 20 mL |
| 0.5 M EDTA | 1 mL |
| Triton X-100 | 10 mL |
| Nuclease-free TDW | 419 mL |
| **Total Volume** | **500 mL** |

*Note: The final concentration of IP buffer is 100 mM Tris-HCl (pH 7.5), 200 mM NaCl, 2% Triton X-100 and 1 mM EDTA.*

- Prepare **washing buffers** as below:

① Washing buffer 1: 50 mM Tris-HCl (pH 7.5), 140 mM NaCl, 1% Triton X-100 and 1mM EDTA

② Washing buffer 2: 50 mM Tris-HCl (pH 7.5), 500 mM NaCl, 1% Triton X-100 and 1mM EDTA

③ Washing buffer 3: 10 mM Tris-HCl (pH 8.0), 250 mM LiCl, 1% Triton X-100 and 1mM EDTA

④ Washing buffer 4 (TE buffer): 10 mM Tris-HCl (pH 8.0), 1mM EDTA

- Prepare fragmentation buffer as blow:

| **Components** | **3.0x10^9^** | **≤ 7.68x10^8^** |
| --- | --- | --- |
| lysis buffer | 125 µl | 100 µl |
| PIC mix | 10 µl | 8 µl |
| Lysozyme (10 mg/ml) | 0.25 µl | 0.2 µl |
| IP buffer | 137.5 µl | 110 µl |

2-3) Resuspend the cell pellet in lysis buffer.

2-4) Add PIC mix and lysozyme.

2-5) Incubate for 30 min at 37 °C on a rotator.

2-6) Add IP buffer and incubate 30 min at 4 °C.

2-7) Shear the lysate by sonication for 25 minutes (3.0x10^9^ and 7.68x10^8^) or 40 minutes (≤ 9.6x10^7^), amplitude 50%, 50” on/10” off, cooling at 4 °C.

2-8) Add 75 µl (3.0x10^9^) or 60 µl (≤ 7.68x10^8^) of wash buffer 1 in fragmented samples.

- Add 1^st^ antibody to the chromatin solution as blow:

| **Components** | **3.0x10^9^** | **≤ 7.68x10^8^** |
| --- | --- | --- |
| 1^st^ Antibody  (RpoD antibody) | 3 µl | 1.5 µl |
| 1^st^ Antibody  (c-Myc antibody) | 7.5 µl | 3.75 µl |

*Continue to incubate 6~8 hours at 4 °C with rotating.*

**3. Antibody-TF complex Binding to Dynabeads**

**-** Prepare elution buffer as below:

| **Components** | **Volume** |
| --- | --- |
| 1 M Tris-HCl (pH 7.5) | 2.5 ml |
| SDS | 0.5 g |
| 0.5 M EDTA | 0.1 ml |
| Nuclease-free TDW | 47.4 ml |
| **Total Volume** | **50 ml** |

*Note: The final concentration of elution buffer is 50 mM Tris-HCl (pH 8.0), 1% SDS and 1 mM EDTA.*

- Prepare bead washing solution as blow:

| **Components** | **Volume** |
| --- | --- |
| BSA powder | 250 mg |
| Ice-cold PBS | 50 ml |
| **Total Volume** | **50 ml** |

- Prepare Dynabeads Pan mouse IgG as blow:

|  | **3.0x10^9^** | **≤ 7.68x10^8^** |
| --- | --- | --- |
| Dynabeads Pan mouse IgG | 30 µl | 15 µl |

*Note: Pull down the beads with the MPC magnet for 30 seconds after washing.*

3-1) Wash with 0.5 ml of bead washing solution 3 times.

3-2) Add Dynabeads Pan mouse IgG to the chromatin solution and incubate overnight at 4 °C with rotating.

**4. STAGE 1: Exonuclease Digestion of Antibody-TF complex**

- Wash the beads with wash buffers as blow:

| **Components** | **Volume** |
| --- | --- |
| Wash buffer 1 (2 times) | 500 µl |
| Wash buffer 2 | 500 µl |
| Wash buffer 3 | 500 µl |
| Wash buffer 4 | 500 µl |

**1) End Repair**

| **Components** | **3.0x10^9^** | **≤ 7.68x10^8^** |
| --- | --- | --- |
| End Repair Buffer (10X) | 2.5 µl | 1 µl |
| End Repair Enzyme Mix | 1.25 µl | 0.5 µl |
| Water | 21.25 µl | 8.5 µl |
| **Total Volume** | **25 µl** | **10 µl** |

*Incubate in a thermal cycler for 30 min at 20 °C and wash the beads with wash buffers.*

**2) dA-Tailing**

| **Components** | **3.0x10^9^** | **≤ 7.68x10^8^** |
| --- | --- | --- |
| Water | 10.5 µl | 4.2 µl |
| dA-Tailing Buffer (10X) | 1.25 µl | 0.5 µl |
| Klenow Fragment (exo^-^) | 0.75 µl | 0.3 µl |
| **Total Volume** | **12.5 µl** | **5 µl** |

*Incubate in a thermal cycler for 30 min at 37 °C and wash the beads with wash buffers.*

**3) Ligation of First Adapter**

| **Components** | **3.0x10^9^** | **≤ 7.68x10^8^** |
| --- | --- | --- |
| Quick Ligation Buffer (2X) | 6.25 µl | 2.5 µl |
| First Adapter (15 µM) | 1.25 µl | 0.5 µl |
| Quick T4 DNA Ligase | 0.25 µl | 0.1 µl |
| Water | 4.75 µl | 1.9 µl |
| **Total Volume** | **12.5 µl** | **5 µl** |

*Incubate in a thermal cycler for 15 min at 20 °C and wash the beads with wash buffers.*

**4) Nick Repair with NEB PreCR Repair Mix**

| **Components** | **3.0x10^9^** | **≤ 7.68x10^8^** |
| --- | --- | --- |
| Water | 10.75 µl | 4.3 µl |
| ThermoPol Buffer (10X) | 1.25 µl | 0.5 µl |
| 10 mM dNTPs | 0.125 µl | 0.05 µl |
| NAD+ (100X) | 0.125 µl | 0.05 µl |
| PreCR Mix | 0.25 µl | 0.1 µl |
| **Total Volume** | **12.5 µl** | **5 µl** |

*Incubate the repair reaction at 37 °C for 15-20 min* *and wash the beads with wash buffers.*

**5) λ Exonuclease Treatment**

| **Components** | **3.0x10^9^** | **≤ 7.68x10^8^** |
| --- | --- | --- |
| Water | 10.75 µl | 4.3 µl |
| λ Exonuclease Buffer (10X) | 1.25 µl | 0.5 µl |
| λ Exonuclease (5,000 U/mL) | 0.5 µl | 0.2 µl |
| **Total Volume** | **12.5 µl** | **5 µl** |

*Incubate at 37 °C for 30 min and wash the beads with wash buffers.*

**6) RecJ Exonuclease Treatment**

| **Components** | **3.0x10^9^** | **≤ 7.68x10^8^** |
| --- | --- | --- |
| Water | 11 µl | 4.4 µl |
| NEBuffer 2 (10X) | 1.25 µl | 0.5 µl |
| RecJ Exonuclease  (30,000 U/mL) | 0.25 µl | 0.1 µl |
| **Total Volume** | **12.5 µl** | **5 µl** |

*Incubate at 37 °C for 30 min and wash the beads with wash buffers.*

**7) Elution of Dynabeads**

| **Components** | **3.0x10^9^** | **≤ 7.68x10^8^** |
| --- | --- | --- |
| Elution buffer | 50 µl | 20 µl |

*Continue to incubate overnight at 65 °C.*

**5. Reverse Crosslinking and DNA Purification**

- Pull down the beads with the MPC magnet for 1 min and save the supernatant.

**1) RNA Removal**

- Prepare RNA removal solution as blow:

| **Components** | **3.0x10^9^** | **≤ 7.68x10^8^** |
| --- | --- | --- |
| RNaseA solution  (100 mg/ml RNaseA) | 0.25 µl | 0.1 µl |
| Washing buffer 4 | 0.75 µl | 0.9 µl |
| **Total Volume** | **1 µl** | **1 µl** |

*Add 1 μl of RNA removal solution and incubate at 37 °C for 2 hours.*

**2) Reverse Crosslinking**

- Prepare protein removal solution as blow:

| **Components** | **3.0x10^9^** | **≤ 7.68x10^8^** |
| --- | --- | --- |
| Protease K (20 mg/ml) | 1 µl | 0.4 µl |
| Washing buffer 4 | 3 µl | 3.6 µl |
| **Total Volume** | **4 µl** | **4 µl** |

*Add 4 μl of protein removal solution and incubate at 55 °C for 2 hours.*

**3) IP-DNA Purification**

- Add 2.5x DNA purification beads (AMPure beads or AccuBead) to the IP-DNA solution as blow:

| **Components** | **3.0x10^9^** | **≤ 7.68x10^8^** |
| --- | --- | --- |
| IP-DNA solution | 55 µl | 22 µl |
| DNA purification beads | 137.5 µl | 55 µl |
| **Total Volume** | **192.5 µl** | **77 µl** |

*Note: Pull down the beads with the MPC magnet for 2 min.*

3-1) Incubate at RT for 10 min and wash the beads with 200 µl of 80% ethanol twice.

3-2) Ensure dried-beads are completely rehydrated and resuspended using 12 µl of nuclease-free water and collect 11 µl of exonuclease treated IP-DNA.

**6. STAGE 2: Construction of Sequencing Library**

**1) Second Strand Synthesis Using Specific Primer and dNTPs**

- Prepare the following reaction mix:

| **Components** | **Volume** |
| --- | --- |
| Exonuclease Treated IP DNA | **11 µl** |
| phi29 DNA Polymerase Buffer (10X) | 2 µl |
| BSA (1 μg/ml) | 4 µl |
| dNTPs (10 mM) | 1 µl |
| Second Strand Synthesis Primer (20 μM) | 1 µl |
| **Total Volume** | **19 µl** |

1-1) Incubate in a thermal cycler for 10 min at 95 °C followed by 5 min at 58 °C.

1-2) Allow to cool to RT by 2 min (primer annealing).

1-3) Add 1 µl phi29 DNA polymerase (10 U/ µl) and incubate for 20 min at 30 °C (primer extension) followed by 10 min at 65 °C (heat inactivation).

**2) dA-Tailing**

2-1) Add 2.5x DNA purification beads (50 µl) in PCR product (20 µl).

2-2) Incubate at RT for 10 min and wash the beads with 200 µl of 80% ethanol twice.

2-3) Ensure dried-beads are completely rehydrated and resuspended using 10 µl of dA-tailing buffer as blow:

| **Components** | **Volume** |
| --- | --- |
| Water | 8.4 µl |
| dA-Tailing Buffer (10X) | 1 µl |
| Klenow Fragment (exo^-^) | 0.6 µl |
| **Total Volume** | **10 µl** |

*Incubate at 37 °C for 30 min followed by 30 min enzyme inactivation at 60°C.*

**3) Ligation of Second Adapter**

3-1) Add 15 µl of second adapter ligation buffer after enzyme inactivation.

- Prepare the following reaction mix:

| **Components** | **Volume** |
| --- | --- |
| Beads with dA-tailed IP- DNA | 10 µl |
| Quick ligation buffer (2X) | 12.5 µl |
| Second Adapter (15 µM) | 0.625 µl |
| Quick T4 DNA Ligase | 0.625 µl |
| Water | 1.25 µl |
| **Total Volume** | **25 µl** |

*Incubate in a thermal cycler for 15 min at 20 °C.*

**4) Remove 3’ overhang with T4 polymerase**

4-1) Add 1.0x PEG/NaCl solution (20% polyethylene glycol 8000/2.5 M NaCl) (25 µl) in beads with adapters-ligated IP-DNA (25 µl).

4-2) Incubate at RT for 10 min and wash the beads with 200 µl of 80% ethanol twice.

4-3) Ensure dried-beads are completely rehydrated and resuspended using 10 µl of T4 polymerase buffer as blow:

- Prepare the following reaction mix:

| **Components** | **Volume** |
| --- | --- |
| Water | 7.4 µl |
| NEBuffer 2.1 | 2 µl |
| dNTPs (10mM) | 0.3 µl |
| T4 DNA polymerase (3 U/ µl) | 0.3 µl |
| **Total Volume** | **10 µl** |

*Incubate in a thermal cycler for 20 min at 12 °C*

4-4) Add 1.0x PEG/NaCl solution (10 µl) in beads with adapters-ligated IP-DNA (10 µl).

4-5) Incubate at RT for 10 min and wash the beads with 200 µl of 80% ethanol twice.

4-6) Ensure dried-beads are completely rehydrated and resuspended using 21 µl of nuclease-free water and collect 20 µl for PCR amplification.

**5) PCR Enrichment**

| **Components** | **Volume** |
| --- | --- |
| IP-DNA with 2 adapter-ligated | 20 µl |
| KAPA Hifi ReadyMix | 25 µl |
| SYBR Green (10X) | 1 µl |
| Non-Indexed Primer (25 µM) | 1 µl |
| Indexed Primer (25 µM) | 1 µl |
| Water | 2 µl |
| **Total Volume** | **50 µl** |

5-1) Amplify using the following PCR protocol using the qPCR thermocycler:

- 98 °C for 2 min
- Amplification cycles of the following:
- 98 °C for 15 s
- 65 °C for 30 s
- 72 °C for 30 s
- 72 °C for 10 min
- Hold at 4 °C

5-2) Add 1.0x DNA purification beads (50 µl) in IP-DNA with PCR reaction mix (50 µl).

5-3) Incubate at RT for 10 min and wash the beads with 200 µl of 80% ethanol twice.

5-4) Ensure dried-beads are completely rehydrated and resuspended using 21 µl of nuclease-free water and collect 20 µl.

5-5) Check library concentration and fragment distribution using the Qubit dsDNA HS kit and Agilent High Sensitivity DNA Kit, respectively.

5-5) Store amplified libraries at -15~-20 °C before sequencing.

**▶ Extra- and intracellular ChIP-mini (ChIP-exo minimization for host-infected bacterial cells)**

**1. *Salmonella* Typhimurium Infection and Crosslinking Preparation**

- Prepare crosslinking mix as below:

| **Components** | **Extra- and intracellular ChIP-mini** |
| --- | --- |
| Formaldehyde (37% w/w) | 280 µl |
| Washing TBS (pH 7.4) | 10 ml |

- 1. Seed macrophage-like cells (J774A.1) in 75T flasks at a plating density of 6x10^6^ per flask under Dulbecco’s modified Eagle’s medium (DMEM) supplemented with 10% (v/v) fetal bovine serum (FBS) and antibiotic antimycotic at 37 °C with 5% CO^2^ in a humidified incubator.
  2. Inoculate *S.* Typhimurium 14028s from glycerol stock to LB broth and incubate at 37 ℃ overnight with constant agitation.
  3. Dilute overnight culture into fresh LB broth and culture at 37˚C overnight with agitation in a shaking incubator before infection.

1-4) Add overnight-grown bacteria to the macrophages (macrophage cell number ≈ 8 x 10^6^) at a multiplicity of infection (MOI) of 10, and centrifuge at 500 xg for 5  minutes at RT and incubate for an additional 30  min.

1-5) Transfer 10ml of DMEM including extracellular bacteria in 75T flask and crosslink adding formaldehyde at RT for 25 min (extracellular Chip-mini).

1-5-1) Centrifuge crosslinked cells at 4,000 xg and wash 3 time with ice-cold TBS.

1-6) Wash infected-macrophage cells in 75T flask three times with PBS.

1-7) Add 10ml DMEM supplemented with 10% FBS and 150 μg ml^−1^ gentamycin and incubate 37 ℃ for 1 hour.

1-8) Replace 10ml DMEM containing 10% FBS with 15 μg mL^−1^ gentamicin and incubate at 37 °C for 6 hours.

1-9) Add formaldehyde to infected-macrophage cells in 75T flask for co-crosslinking at RT for 25 min (intracellular ChIP-mini).

1-9-1) Centrifuge at 500 xg co-crosslinked cells and wash 3 time with ice-cold TBS.

1-10) Remove supernatant and transfer co-crosslinked cells to 1.75ml tube.

*Note: The number of intracellular bacteria in the host cells is less than 4.8x10^6^, the efficiency of intracellular ChIP-mini can be significantly reduced.*

**2. Fragmentation and Preparation of Antibody-TF complex**

=> This step removes cytoplasmic proteins from macrophages that disrupt immunoprecipitation of ChIP-mini.

- Prepare host lysis mix as below:

| **Components** | **Intracellular ChIP-mini** |
| --- | --- |
| Triton X-100 | 10 µl |
| PBS (pH 7.4) | 990 µl |
| **Total Volume** | **1 ml** |

2-1) Resuspend co-crosslinked cells in 1 ml of host lysis buffer using pipetting and incubate RT at 20 min.

2-2) Centrifuge at 20,000 xg and remove all supernatant in 1.75ml tube (*Do not disturb the cell pellet*).

- Prepare fragmentation buffer as blow:

| **Components** | **Extra- and intracellular ChIP-mini** |
| --- | --- |
| lysis buffer | 100 µl |
| PIC mix | 8 µl |
| Lysozyme  (10 mg/ml) | 0.2 µl |
| IP buffer | 110 µl |

2-3) Resuspend the cell pellet in 100 µl of lysis buffer.

2-4) Add 8 µl of PIC mix and 0.2 µl of lysozyme.

2-5) Incubate for 30 min at 37 °C on a rotator.

2-6) Add 110 µl of IP buffer and incubate 30 min at 4 °C.

2-7) Shear the lysate by sonication for 40 minutes, amplitude 50%, 50” on/10” off, cooling at 4 °C.

2-8) Add 60 µl of wash buffer 1 in fragmented samples.

- Add 1^st^ antibody to the chromatin solution as blow:

| **Components** | **Extra- and intracellular ChIP-mini** |
| --- | --- |
| 1^st^ Antibody  (RpoD antibody) | 1.5 µl |
| 1^st^ Antibody  (c-Myc antibody) | 3.75 µl |

*Continue to incubate 6~8 hours at 4 °C with rotating.*

**3. Antibody-TF complex Binding to Dynabeads**

- Prepare bead washing solution as blow:

| **Components** | **Volume** |
| --- | --- |
| BSA powder | 250 mg |
| Ice-cold PBS | 50 ml |
| **Total Volume** | **50 ml** |

- Prepare Dynabeads Pan mouse IgG as blow:

|  | **Extra- and intracellular ChIP-mini** |
| --- | --- |
| Dynabeads Pan mouse IgG | 15 µl |

*Note: Pull down the beads with the MPC magnet for 30 seconds after washing.*

3-1) Wash with 0.5 ml of bead washing solution 3 times.

3-2) Add Dynabeads Pan mouse IgG to the chromatin solution and incubate overnight at 4 °C with rotating.

**4. STAGE 1: Exonuclease Digestion of Antibody-TF complex**

- Wash the beads with wash buffers as blow:

|  | **Extra- and intracellular ChIP-mini** |
| --- | --- |
| Wash buffer 1 (2 times) | 500 µl |
| Wash buffer 2 | 500 µl |
| Wash buffer 3 | 500 µl |
| Wash buffer 4 | 500 µl |

**1) End Repair**

| **Components** | | **Extracellular**  **ChIP-mini** | **Intracellular**  **ChIP-mini** |
| --- | --- | --- | --- |
| End Repair Buffer (10X) | 1 µl | | 2 µl |
| End Repair Enzyme Mix | | 0.5 µl | 1 µl |
| Water | | 8.5 µl | 17 µl |
| **Total Volume** | | **10 µl** | **20 µl** |

*Incubate in a thermal cycler for 30 min at 20 °C and wash the beads with wash buffers.*

**2) dA-Tailing**

| **Components** | **Extracellular**  **ChIP-mini** | **Intracellular**  **ChIP-mini** |
| --- | --- | --- |
| Water | 4.2 µl | 8.4 µl |
| dA-Tailing Buffer (10X) | 0.5 µl | 1 µl |
| Klenow Fragment (exo^-^) | 0.3 µl | 0.6 µl |
| **Total Volume** | **5** **µl** | **10 µl** |

*Incubate in a thermal cycler for 30 min at 37 °C and wash the beads with wash buffers.*

**3) Ligation of First Adapter**

| **Components** | **Extracellular**  **ChIP-mini** | **Intracellular**  **ChIP-mini** |
| --- | --- | --- |
| Quick Ligation Buffer (2X) | 2.5 µl | 5 µl |
| First Adapter (15 µM) | 0.5 µl | 1 µl |
| Quick T4 DNA Ligase | 0.1 µl | 0.2 µl |
| Water | 1.9 µl | 3.8 µl |
| **Total Volume** | **5 µl** | **10 µl** |

*Incubate in a thermal cycler for 15 min at 20 °C and wash the beads with wash buffers.*

**4) Nick Repair with NEB PreCR Repair Mix**

| **Components** | **Extracellular**  **ChIP-mini** | **Intracellular**  **ChIP-mini** |
| --- | --- | --- |
| Water | 4.3 µl | 8.6 µl |
| ThermoPol Buffer (10X) | 0.5 µl | 1.0 µl |
| 10 mM dNTPs | 0.05 µl | 0.1 µl |
| NAD+ (100X) | 0.05 µl | 0.1 µl |
| PreCR Mix | 0.1 µl | 0.2 µl |
| **Total Volume** | **5 µl** | **10 µl** |

*Incubate the repair reaction at 37 °C for 15-20 min* *and wash the beads with wash buffers.*

**5) λ Exonuclease Treatment**

| **Components** | **Extracellular**  **ChIP-mini** | **Intracellular**  **ChIP-mini** |
| --- | --- | --- |
| Water | 4.3 µl | 8.6 µl |
| λ Exonuclease Buffer (10X) | 0.5 µl | 1 µl |
| λ Exonuclease (5,000 U/mL) | 0.2 µl | 0.4 µl |
| **Total Volume** | **5 µl** | **10 µl** |

*Incubate at 37 °C for 30 min and wash the beads with wash buffers.*

**6) RecJ Exonuclease Treatment**

| **Components** | **Extracellular**  **ChIP-mini** | **Intracellular**  **ChIP-mini** |
| --- | --- | --- |
| Water | 4.4 µl | 8.8 µl |
| NEBuffer 2 (10X) | 0.5 µl | 1 µl |
| RecJ Exonuclease  (30,000 U/mL) | 0.1 µl | 0.2 µl |
| **Total Volume** | **5 µl** | **10 µl** |

*Incubate at 37 °C for 30 min and wash the beads with wash buffers.*

**7) Elution of Dynabeads**

| **Components** | **Extra- and intracellular**  **ChIP-mini** |
| --- | --- |
| Elution buffer | 20 µl |

*Continue to incubate overnight at 65 °C.*

**5. Reverse Crosslinking and DNA Purification**

- Pull down the beads with the MPC magnet and save supernatant.

**1) RNA Removal**

- Prepare RNA removal solution as blow:

| **Components** | **Extra- and intracellular *ChIP-mini*** |
| --- | --- |
| RNaseA solution  (100 mg/ml RNaseA) | 0.1 µl |
| Washing buffer 4 | 0.9 µl |
| **Total Volume** | **1 µl** |

*Add 1 μl of RNA removal solution and incubate at 37 °C for 2 hours.*

**2) Reverse Crosslinking**

- Prepare protein removal solution as blow:

| **Components** | ***Extra- and intracellular ChIP-mini*** |
| --- | --- |
| Protease K (20 mg/ml) | 0.4 µl |
| Washing buffer 4 | 3.6 µl |
| **Total Volume** | **4 µl** |

*Add 4 μl of protein removal solution and incubate at 55 °C for 2 hours.*

**3) IP-DNA Purification**

- Add 2.5x DNA purification beads (AMPure beads or AccuBead) to the IP-DNA solution as blow:

| **Components** | **Extra- and intracellular ChIP-mini** |
| --- | --- |
| IP-DNA solution | 22 µl |
| DNA purification beads | 55 µl |
| **Total Volume** | **77 µl** |

*Note: Pull down the beads with the MPC magnet for 2 min.*

3-1) Incubate at RT for 10 min and wash the beads with 200 µl of 80% ethanol twice.

3-2) Ensure dried-beads are completely rehydrated and resuspended using 12 µl of nuclease-free water and collect 11 µl of exonuclease treated IP-DNA.

**6. STAGE 2: Construction of Sequencing Library**

**1) Second Strand Synthesis Using Specific Primer and dNTPs**

- Prepare the following reaction mix:

| **Components** | **Volume** |
| --- | --- |
| Exonuclease Treated IP DNA | **11 µl** |
| phi29 DNA Polymerase Buffer (10X) | 2 µl |
| BSA (1 μg/ml) | 4 µl |
| dNTPs (10 mM) | 1 µl |
| Second Strand Synthesis Primer (20 μM) | 1 µl |
| **Total Volume** | **19 µl** |

1-1) Incubate in a thermal cycler for 10 min at 95 °C followed by 5 min at 58 °C.

1-2) Allow to cool to RT by 2 min (primer annealing).

1-3) Add 1 µl phi29 DNA polymerase (10 U/ µl) and incubate for 20 min at 30 °C (primer extension) followed by 10 min at 65 °C (heat inactivation).

**2) dA-Tailing**

2-1) Add 2.5x DNA purification beads (50 µl) in PCR product (20 µl).

2-2) Incubate at RT for 10 min and wash the beads with 200 µl of 80% ethanol twice.

2-3) Ensure dried-beads are completely rehydrated and resuspended using 10 µl of dA-tailing buffer as blow:

| **Components** | **Volume** |
| --- | --- |
| Water | 8.4 µl |
| dA-Tailing Buffer (10X) | 1 µl |
| Klenow Fragment (exo^-^) | 0.6 µl |
| **Total Volume** | **10 µl** |

*Incubate at 37 °C for 30 min followed by 30 min enzyme inactivation.*

**3) Ligation of Second Adapter**

3-1) Add 15 µl of second adapter ligation buffer after enzyme inactivation.

- Prepare the following reaction mix:

| **Components** | **Volume** |
| --- | --- |
| Beads with dA-tailed IP- DNA | 10 µl |
| Quick ligation buffer (2X) | 12.5 µl |
| Second Adapter (15 µM) | 0.625 µl |
| Quick T4 DNA Ligase | 0.625 µl |
| Water | 1.25 µl |
| **Total Volume** | **25 µl** |

*Incubate in a thermal cycler for 15 min at 20 °C.*

**4) Remove 3’ overhang with T4 polymerase**

4-1) Add 1.0x PEG/NaCl solution (25 µl) in beads with adapters-ligated IP-DNA (25 µl).

4-2) Incubate at RT for 10 min and wash the beads with 200 µl of 80% ethanol twice.

4-3) Ensure dried-beads are completely rehydrated and resuspended using 10 µl of T4 polymerase buffer as blow:

- Prepare the following reaction mix:

| **Components** | **Volume** |
| --- | --- |
| Water | 7.4 µl |
| NEBuffer 2.1 | 2 µl |
| dNTPs (10mM) | 0.3 µl |
| T4 DNA polymerase (3 U/ µl) | 0.3 µl |
| **Total Volume** | **10 µl** |

*Incubate in a thermal cycler for 20 min at 12 °C.*

4-4) Add 1.0x PEG/NaCl solution (10 µl) in beads with adapters-ligated IP-DNA (10 µl).

4-5) Incubate at RT for 10 min and wash the beads with 200 µl of 80% ethanol twice.

4-6) Ensure dried-beads are completely rehydrated and resuspended using 21 µl of nuclease-free water and collect 20 µl for PCR amplification.

**5) PCR Enrichment**

| **Components** | **Volume** |
| --- | --- |
| IP-DNA with 2 adapter-ligated | 20 µl |
| KAPA Hifi ReadyMix | 25 µl |
| SYBR Green (10X) | 1 µl |
| Non-Indexed Primer (25 µM) | 1 µl |
| Indexed Primer (25 µM) | 1 µl |
| Water | 2 µl |
| **Total Volume** | **50 µl** |

5-1) Amplify using the following PCR protocol using the qPCR thermocycler:

- 98 °C for 2 min
- Amplification cycles of the following:
- 98 °C for 15 s
- 65 °C for 30 s
- 72 °C for 30 s
- 72 °C for 10 min
- Hold at 4 °C

5-2) Add 1.0x DNA purification beads (50 µl) in IP-DNA with PCR reaction mix (50 µl).

5-3) Incubate at RT for 10 min and wash the beads with 200 µl of 80% ethanol twice.

5-4) Ensure dried-beads are completely rehydrated and resuspended using 21 µl of nuclease-free water and collect 20 µl.

5-5) Check library concentration and fragment distribution using the Qubit dsDNA HS kit and Agilent High Sensitivity DNA Kit, respectively.

5-5) Store amplified libraries at -15~-20 °C before sequencing.

**▶ Immunomagnetic separation (IMS)**

To reduce the host DNA contamination in intracellular ChIP-mini libraries, immunomagnetic separation (IMS) was employed (12,13). A rabbit anti-*Salmonella* antibody (ab35156, Abcam) that specifically recognizes *Salmonella* LPS was used for the 1^st^ antibody. To prepare the antibody-magnetic bead complex, 50 μl of Dynabeads M-280 Sheep Anti-Rabbit IgG magnetic beads (Invitrogen) were stringently washed three times with bead washing solution (5 mg/ml BSA) and resuspended in 400 μl of PBS. Next, 15 μl of the 1^st^ antibody was added and incubated with rotation at RT for 1 hour. Following incubation, the antibody-magnetic beads were kept rotating at 4 °C until the lysis of the co-crosslinked cells.

Co-crosslinked cells (*S.* Typhimurium-infected macrophages at 2 hours post-infection) were transferred to a 1.75 ml tube and lysed with 1% Triton X-100. The lysed macrophage cells were then centrifuged at 20,000 xg for 5 minutes at 4 °C, and the resulting cell pellet was resuspended in 400 μl of PBS. Prior to IMS, the supernatant of the antibody-magnetic beads was removed using an MPC magnet for 30 seconds. The resuspended cell solution was then added to the antibody-magnetic beads and incubated with rotation at RT for 1 hour. Following incubation, the *Salmonella*-captured beads were gently washed with PBS.

*Salmonella*-captured beads were resuspended in 108.2 μl of lysis buffer mix, protease inhibitor cocktail, and lysozyme. 110 μl of the IP buffer was added to lyse cells. DNA for H-NS samples were fragmented using sonication (40 minutes with 50s on and 10s off intervals and amplitude 50%). Antibody-magnetic beads in sonicated samples were removed using the MPC magnet at 30 seconds. ChIP was proceeded using 3.75 μl of c-Myc antibody (9E10, Biolegend). Next, 15 μl of Dynabeads Pan Mouse IgG magnetic beads (Invitrogen) was used, followed by stringent washings. The subsequent steps follow the same experimental procedure of intracellular ChIP-mini. ChIP-mini experiments combined with IMS were also performed in biological duplicates.

**References**

1. Rhee, H.S. and Pugh, B.F. (2011) Comprehensive genome-wide protein-DNA interactions detected at single-nucleotide resolution. *Cell*, **147**, 1408-1419.

2. Seo, S.W., Kim, D., Latif, H., O’Brien, E.J., Szubin, R. and Palsson, B.O.J.N.c. (2014) Deciphering Fur transcriptional regulatory network highlights its complex role beyond iron metabolism in Escherichia coli. **5**, 1-10.

3. Kim, D., Seo, S.W., Gao, Y., Nam, H., Guzman, G.I., Cho, B.-K. and Palsson, B.O. (2018) Systems assessment of transcriptional regulation on central carbon metabolism by Cra and CRP. *Nucleic Acids Res.*, **46**, 2901-2917.

4. Seo, S.W., Kim, D., O’Brien, E.J., Szubin, R. and Palsson, B.O.J.N.c. (2015) Decoding genome-wide GadEWX-transcriptional regulatory networks reveals multifaceted cellular responses to acid stress in Escherichia coli. **6**, 1-8.

5. Brind’Amour, J., Liu, S., Hudson, M., Chen, C., Karimi, M.M. and Lorincz, M.C.J.N.c. (2015) An ultra-low-input native ChIP-seq protocol for genome-wide profiling of rare cell populations. **6**, 1-8.

6. Wilkening, S., Tekkedil, M.M., Lin, G., Fritsch, E.S., Wei, W., Gagneur, J., Lazinski, D.W., Camilli, A. and Steinmetz, L.M.J.B.g. (2013) Genotyping 1000 yeast strains by next-generation sequencing. **14**, 1-10.

7. Bang, I., Nong, L.K., Park, J.Y., Le, H.T., Lee, S.-M., Kim, D.J.C. and Journal, S.B. (2022) ChEAP: ChIP-exo analysis pipeline and the investigation of Escherichia coli RpoN protein-DNA interactions.

8. Bang, I., Lee, S.-M., Park, S., Park, J.Y., Nong, L.K., Gao, Y., Palsson, B.O. and Kim, D.J.B.i.B. (2023) Deep-learning optimized DEOCSU suite provides an iterable pipeline for accurate ChIP-exo peak calling. **24**, bbad024.

9. Love, M.I., Huber, W. and Anders, S.J.G.b. (2014) Moderated estimation of fold change and dispersion for RNA-seq data with DESeq2. **15**, 1-21.

10. Park, J.Y., Lee, S.-M., Ebrahim, A., Scott-Nevros, Z.K., Kim, J., Yang, L., Sastry, A., Seo, S.W., Palsson, B.O. and Kim, D. (2023) Model-driven experimental design workflow expands understanding of regulatory role of Nac in Escherichia coli. *NAR genom. bioinform.*, **5**, lqad006.

11. Jennings, E., Thurston, T.L., Holden, D.W.J.C.H. and Microbe. (2017) Salmonella SPI-2 type III secretion system effectors: molecular mechanisms and physiological consequences. **22**, 217-231.

12. Chaudhari, N.M., Gupta, V.K. and Dutta, C. (2016) BPGA-an ultra-fast pan-genome analysis pipeline. *Scientific reports*, **6**, 24373.

13. Wang, Z., Cai, R., Gao, Z., Yuan, Y. and Yue, T. (2020) Immunomagnetic separation: An effective pretreatment technology for isolation and enrichment in food microorganisms detection. *Comprehensive Reviews in Food Science and Food Safety*, **19**, 3802-3824.
